# Reproductive outcomes of pregnancy after breast cancer: an updated systematic review and meta-analysis

**DOI:** 10.3389/fonc.2025.1569109

**Published:** 2025-09-26

**Authors:** Qingya Song, Heting Mei, Wenping Lu, Weijia Zhang, Jiaxin Liu, Xiyue Wang, Zhili Zhuo, Lei Chang

**Affiliations:** Department of Oncology, China Academy of Chinese Medical Sciences Guang’anmen Hospital, Beijing, China

**Keywords:** breast cancer, pregnancy, reproductive outcomes, meta, review

## Abstract

**Background:**

Reproductive outcomes following pregnancy in premenopausal women with breast cancer (BC) remain contentious, and few meta-analyses have adequately addressed these questions. This systematic review and meta-analysis aimed to provide the most up-to-date and comprehensive evidence on the subject.

**Methods:**

Ten electronic databases were searched in September 2024 using the terms “Breast Neoplasms” AND “Pregnancy OR Fertilization OR Parturition OR Fertility OR Obstetrics”. Key inclusion criteria focused on reproductive outcomes in premenopausal women with BC compared to healthy populations. Primary outcomes included pregnancy outcomes, obstetrical outcomes, fetal outcomes, and pregnancy complications. The review adhered to the Preferred Reporting Items for Systematic Reviews and Meta-Analyses (PRISMA) guidelines and the Meta - analysis of Observational Studies in Epidemiology (MOOSE) statement. Depending on the study type, dichotomous variables were analyzed using relative risk, odds ratio, hazard ratio, standardized birth ratio, and 95% confidence interval. To preserve the accuracy of findings, original effect measures were used, while other studies were addressed in the systematic review.

**Results:**

Out of 35,324 records identified, 26 studies met the inclusion criteria. The meta-analysis indicated that women with breast cancer had lower pregnancy prevalence, lower completed pregnancy rate, lower childbirth rate, lower birth trauma rate, and higher rates of cesarean delivery and preterm birth compared to healthy controls. Offspring of women with breast cancer had higher risks of very low birth weight, low birth weight, fetal abnormalities, and a lower live birth rate. The systematic review further showed increased risks of intrapartum hemorrhage, induced delivery, spontaneous delivery, failed induction of labor, prolonged labor, fetal stress, and delivery-related complications in this group, along with a lower rate of full-term delivery and reduced incidence of gestational hypertension.

**Conclusions:**

Pregnancy outcomes after breast cancer are often unsatisfactory. Patients and clinicians should approach pregnancy planning with care, ensuring thorough assessment and appropriate testing throughout the process.

**Systematic review registration:**

https://www.crd.york.ac.uk/PROSPERO/, identifier CRD42024499971.

## 1 Introduction

According to 2022 statistics, breast cancer (BC) ranked as the second most common malignant tumor and remained the leading cancer among women (1). With the increasing trend of BC diagnoses in younger individuals and the general delay in childbearing age, more women are now confronting the issue of pregnancy following a BC diagnosis (2). However, treatments for BC can result in irreversible ovarian dysfunction, premature menopause, and infertility, which are major concerns for affected women (3–6). The reproductive outcomes after a BC diagnosis remain controversial, raising persistent concerns among both patients and clinicians (7, 8).

This systematic review and meta-analysis aimed to present the most current and comprehensive evidence on the topic, offering evidence-based support for fertility counseling among patients with a history of breast cancer who later conceive, as well as their physicians. Compared to the latest high-quality meta-analysis in this field (9), our review applied a wider search strategy, incorporated more recent studies, and generated more detailed and comprehensive outcomes. Articles that could not be pooled in the meta-analysis were included in the qualitative synthesis.

## 2 Materials and methods

### 2.1 Search strategy

This study followed the Preferred Reporting Items for Systematic Reviews and Meta-Analyses (PRISMA) guidelines (10) and the MOOSE statement (11). The protocol was registered in PROSPERO. The experimental group consisted of premenopausal women who became pregnant after a BC diagnosis, while the control group comprised matched healthy individuals. No specific interventions were imposed.

The following databases were searched: PubMed, EMBASE, Cochrane Library, Science Direct, Web of Science, Scopus, CNKI, VIP, Wan Fang, and SinoMed. Searches included all records published up to September 2024, without language restrictions. Both subject headings and free-text terms were used. The search strategy included the terms “Breast Neoplasms” AND “Pregnancy OR Fertilization OR Parturition OR Fertility OR Obstetrics”. Full search details are provided in **Supplementary 1**.

### 2.2 Selection criteria

The inclusion criteria were as follows: 1) premenopausal women, 2) studies reporting on pregnancy following a primary BC diagnosis, 3) studies providing extractable or measurable data on at least one of the four predefined outcomes (pregnancy outcomes, obstetrical outcomes, fetal outcomes, and pregnancy complications), and 4) prospective and retrospective cohort studies, clinical trials, case–control studies, and case series.

The exclusion criteria included the following: 1) patients with other concurrent malignancies, 2) pregnancy-associated BC (diagnosed during pregnancy or within 1 year postpartum), 3) case series with fewer than 10 patients, and 4) ongoing studies with unpublished or unavailable data at the time of the search.

### 2.3 Literature screening and data extraction

Titles and abstracts were independently screened by Song and Mei, with disagreements resolved by Lu. Full-text review and data extraction were conducted independently by five reviewers. A separate pair of reviewers completed a pilot test prior to full data extraction. Extracted variables included first author, year of publication, country, study design, follow-up duration, tumor characteristics, pregnancy outcomes, obstetrical outcomes, fetal outcomes, and pregnancy complications.

### 2.4 Quality evaluation

The quality of included studies was independently assessed by Song and Mei using the Newcastle - Ottawa Scale (NOS) (12), with disagreements resolved by Lu. According to the NOS, studies scoring between 7 and 9 were considered high quality, those scoring 4 to 6 were classified as medium quality, and those with scores below 4 were rated as low quality (12, 13).

### 2.5 Statistical analysis

Meta-analysis was carried out using Stata 17.0 and R version 4.3.0. For dichotomous outcomes, relative risk (RR) or odds ratio (OR) with 95% CI was applied based on the type of study. Heterogeneity was assessed using the chi-square test. A *p*-value <0.05 or *I* ^2^ > 50% indicated statistical heterogeneity, prompting the use of a random-effects model. If these criteria were not met, a fixed-effects model was applied. Sensitivity analysis was conducted through one-by-one elimination. Publication bias was evaluated using funnel plots and Egger’s test, with a significance level of α = 0.05.

To reduce error, most results were reported using adjusted effect measures such as adjusted RR, adjusted OR, adjusted hazard ratio (HR), and standardized birth ratio (SBR). Original effect measures were retained to preserve accuracy. Identical effect sizes for the same outcome indicators were quantitatively combined. Studies that could not be included in the meta-analysis were addressed in the systematic review.

## 3 Results

**Figure 1:**
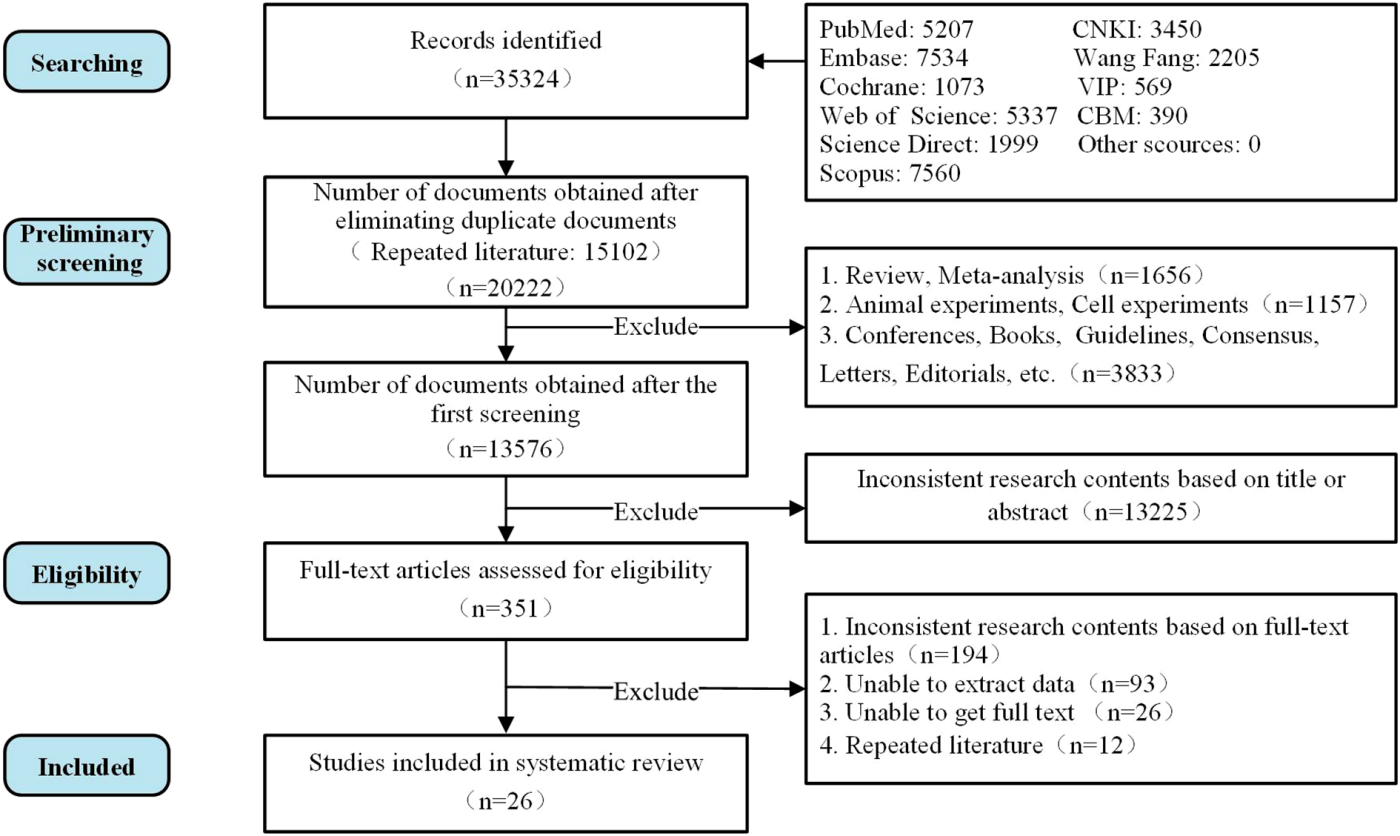
PRISMA 2020 flow diagram of study selection process.

Out of 35,324 records identified, 26 studies were included in the systematic review. The PRISMA flow diagram is presented in **Figure 1**.

The included studies were conducted across 10 countries, with the United States contributing the largest number. When grouped in 5-year intervals, the period from 2020 to 2024 accounted for the highest number of publications (nine articles). The basic characteristics of the included studies are summarized in **eTable 1** and **eTable 2** (**Supplementary 2**).

**Figure 2:**
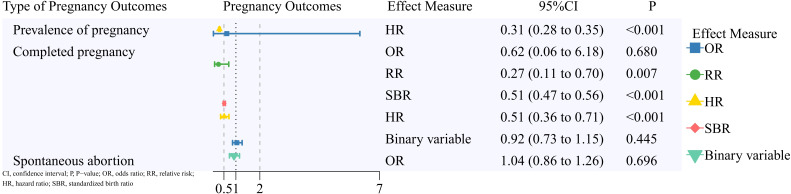
Pregnancy outcomes of patients with a pregnancy after breast cancer.

**Figure 3:**
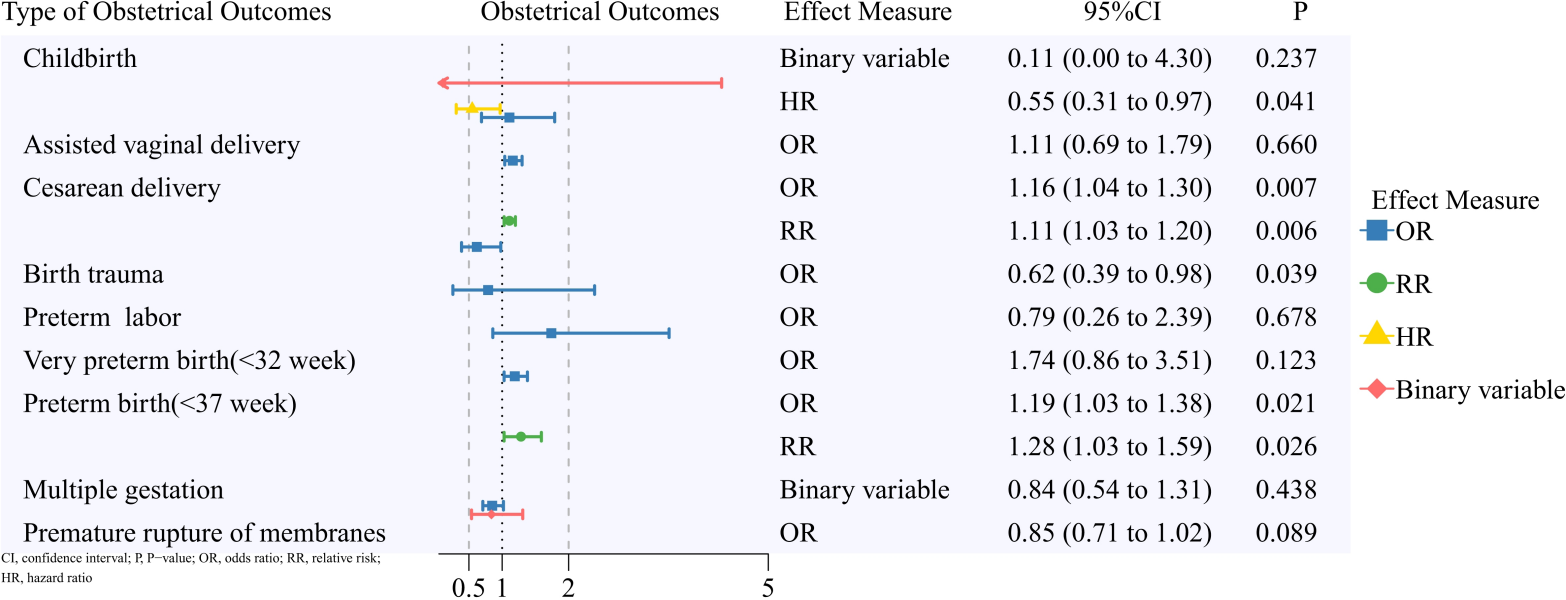
Obstetrical outcomes of patients with a pregnancy after breast cancer.

**Figure 4:**
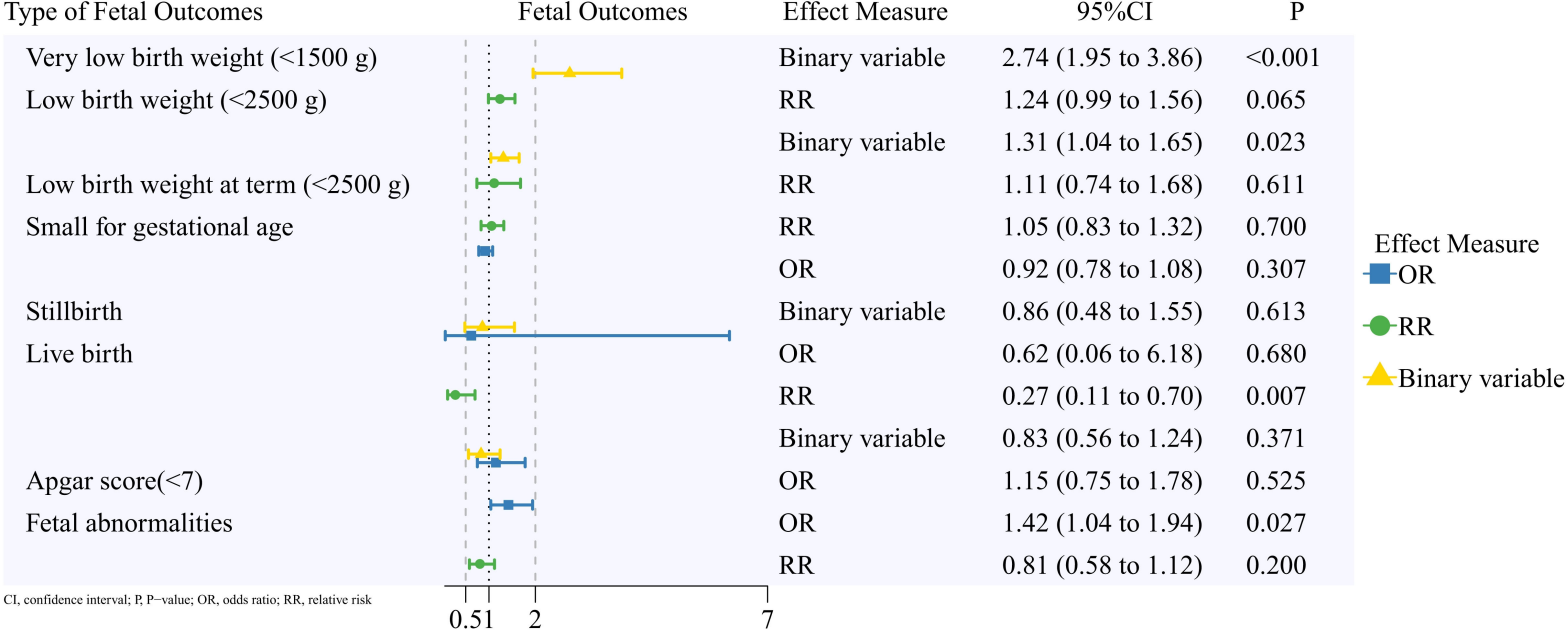
Fetal outcomes of patients with a pregnancy after breast cancer.

**Figure 5:**
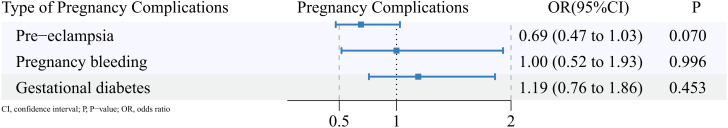
Pregnancy complications of patients with a pregnancy after breast cancer.

A summary of the pooled results on reproductive outcomes is displayed in **Figures 2**–**5**. Corresponding forest plots, publication bias assessments, and sensitivity analyses for the four outcome categories are provided in **eFigures 1** through 4 (**Supplementary 3**).

### 3.1 Study quality evaluation

Quality assessment results are shown in **eTable 3** (**Supplementary 2**). Among the cohort studies, 11 were classified as high quality, while 14 were medium quality. The single case–control study included was rated as medium quality.

### 3.2 Pregnancy outcomes

A total of 20 studies were included in the analysis of pregnancy outcomes.

#### 3.2.1 Prevalence of pregnancy

Two studies (14, 15) were included in the meta-analysis. Patients with BC had a lower pregnancy rate (HR, 0.31; 95% CI, 0.28 to 0.35).

One study (16) reported a lower pregnancy rate among patients with BC who used autologous oocytes compared to healthy individuals undergoing assisted reproductive technology (OR, 0.20; 95% CI, 0.13 to 0.32).

#### 3.2.2 Completed pregnancy

Thirteen studies were included in the meta-analysis. Effect measures included OR (two studies) (16, 17), RR (two studies) (18, 19), SBR (two studies) (20, 21), HR (three studies) (22–24), and RR computed from binary data (four studies) (25–28). Except for the non-significant results in a few studies, patients with BC showed a lower completed pregnancy rate (RR, 0.27; 95% CI, 0.11 to 0.70; SBR, 0.51; 95% CI, 0.47 to 0.56; HR, 0.51; 95% CI, 0.36 to 0.71).

#### 3.2.3 Spontaneous abortion

Two studies (17, 23) were included in the meta-analysis. No significant difference was found (OR, 1.04; 95% CI, 0.86 to 1.26).

However, one study (29) reported a higher risk of miscarriage before 20 weeks’ gestation in patients with BC (RR, 1.7; 95% CI, 1.1 to 2.8). Another study (27) reported miscarriage rates of 25% in the BC group and 13.99% in the healthy population.

#### 3.2.4 Induced abortion

One study (17) reported no significant difference in the rate of induced abortion (OR, 1.12; 95% CI, 0.44 to 2.83).

Another study (27) reported that the cumulative rate of induced abortion was 4.17% in patients with BC and 16.70% in healthy controls.

### 3.3 Obstetrical outcomes

A total of 16 studies were included in this analysis. No significant differences were observed for the following outcomes: assisted vaginal delivery (OR, 1.11; 95% CI, 0.69 to 1.79) (28, 30), abnormalities of the forces of labor (Standardized Incidence Ratio (SIR), 0.83; 95% CI, 0.64 to 1.09) (31), fetal malpresentation (SIR, 0.98; 95% CI, 0.82 to 1.16; OR, 0.77; 95% CI, 0.34 to 1.75) (17, 31), obstructed labor due to fetal malposition or malpresentation (SIR, 1.13; 95% CI, 0.88 to 1.45) (31), perineal laceration (SIR, 0.95; 95% CI, 0.88 to 1.02) (31), puerperal infections excluding sepsis (SIR, 1.43; 95% CI, 0.97 to 2.12) (31), retained placenta and membranes without hemorrhage (SIR, 1.16; 95% CI, 0.81 to 1.67) (31), preterm labor (OR, 0.79; 95% CI, 0.26 to 2.39) (17, 23), prolonged pregnancy beyond 42 weeks (OR, 0.87; 95% CI, 0.28 to 2.71; SIR, 1.09; 95% CI, 0.94 to 1.27) (28, 31), multiple gestation (RR, 0.84; 95% CI, 0.54 to 1.31) (23, 27), hydramnios or oligohydramnios (OR, 1.15; 95% CI, 0.83 to 1.58; SIR, 0.83; 95% CI, 0.51 to 1.36) (23, 31), and premature rupture of membranes (OR, 0.85; 95% CI, 0.71 to 1.02; SIR, 1.00; 95% CI, 0.87 to 1.16) (23, 30, 31).

#### 3.3.1 Obstetrical hemorrhage

One study (23) found no difference in obstetrical hemorrhage (OR, 1.00; 95% CI, 0.75 to 1.34). However, another study (31) reported an increased risk of intrapartum hemorrhage among patients with BC (SIR, 1.57; 95% CI, 1.03 to 2.41), while no significant difference was noted in postpartum hemorrhage (SIR, 1.11; 95% CI, 0.98 to 1.25).

#### 3.3.2 Childbirth

Four studies were included in this meta-analysis. Effect measures included RR from binary data (two studies) (21, 25) and HR (two studies) (23, 24). Patients with BC showed a lower childbirth rate (HR, 0.55; 95% CI, 0.31 to 0.97).

Additionally, one study (31) reported a reduced childbirth rate in patients with BC (observed-to-expected ratio, 0.49; 95% CI, 0.47 to 0.52).

#### 3.3.3 Spontaneous delivery

One study (17) found that patients with BC had a higher rate of spontaneous delivery compared to healthy controls (OR, 1.96; 95% CI, 1.26 to 3.05).

#### 3.3.4 Cesarean delivery

Six studies were included in this analysis. Effect measures consisted of OR (four studies) (17, 28, 30, 32) and RR (two studies) (20, 33). Overall, patients with BC had a higher rate of cesarean delivery (OR, 1.16; 95% CI, 1.04 to 1.30; RR, 1.11; 95% CI, 1.03 to 1.20).

One study (27) reported that the cesarean delivery rate was 37.5% in the BC group, compared to 26.68% in the healthy population.

#### 3.3.5 Induced delivery

One study (30) reported that patients with BC had a higher rate of induced delivery (OR, 1.27; 95% CI, 1.04 to 1.56). Additionally, another study (31) suggested that these patients may be at increased risk of unsuccessful induction of labor (SIR, 1.63; 95% CI, 1.21 to 2.20).

#### 3.3.6 Birth trauma

Two studies (28, 30) were included in this meta-analysis. Overall, patients with BC had a lower rate of birth trauma (OR, 0.62; 95% CI, 0.39 to 0.98).

#### 3.3.7 Long labor

A study (31) reported that patients with BC may face a higher risk of prolonged labor (SIR, 1.17; 95% CI, 1.03 to 1.31).

#### 3.3.8 Very preterm birth (32 weeks)

Three studies (28, 30, 32) were included in the meta-analysis. No significant difference was observed (OR, 1.74; 95% CI, 0.86 to 3.51). However, one study (33) suggested a higher risk of very preterm birth among BC patients (RR, 1.5; 95% CI, 1.0 to 2.5).

#### 3.3.9 Preterm birth (37 weeks)

Ten studies were included in this meta-analysis. Effect measures included OR (five studies) (17, 23, 28, 30, 32) and RR (five studies) (20, 33–36). Patients with BC had a higher rate of preterm birth overall (OR, 1.19; 95% CI, 1.03 to 1.38; RR, 1.28; 95% CI, 1.03 to 1.59).

One study (31) reported no significant difference between groups (SIR, 1.07; 95% CI, 0.91 to 1.27), while another (37) found preterm birth rates of 6.98% in BC patients and 4.13% in the healthy population.

#### 3.3.10 Full-term delivery

One study (23) reported that patients with BC had a lower rate of full-term delivery (OR, 0.78; 95% CI, 0.68 to 0.90).

#### 3.3.11 Delivery complications

A study (28) reported more frequent delivery complications among patients with BC (OR, 1.50; 95% CI, 1.20 to 1.90).

### 3.4 Fetal outcomes

A total of 19 studies were included in this analysis. No significant differences were observed for the following outcomes: low birth weight at term (<2,500 g) (RR, 1.11; 95% CI, 0.74 to 1.68) (33, 34), small for gestational age (RR, 1.05; 95% CI, 0.83 to 1.32; OR, 0.92; 95% CI, 0.78 to 1.08) (20, 30, 32, 33, 35, 38), stillbirth (RR, 0.86; 95% CI, 0.48 to 1.55) (17, 27, 28, 30, 34, 39), birth weight >4,500 g (OR, 1.10; 95% CI, 0.63 to 1.92) (28), placental disorders (SIR, 0.87; 95% CI, 0.58 to 1.33; OR, 0.79; 95% CI, 0.29 to 2.11) (30, 31), placenta previa (SIR, 0.98; 95% CI, 0.65 to 1.48) (31), and umbilical cord complications (SIR, 1.14; 95% CI, 0.90 to 1.45) (31).

#### 3.4.1 Very low birth weight (<1,500 g)

Two studies (28, 33) were included in this meta-analysis. Offspring of patients with BC had a higher risk of very low birth weight (RR, 2.74; 95% CI, 1.95 to 3.86).

#### 3.4.2 Low birth weight (<2,500 g)

Eight studies were included in this meta-analysis. Effect measures included RR (five studies) (20, 33–36) and RR calculated from binary data (three studies) (28, 30, 37). Overall, offspring of patients with BC had a higher risk of low birth weight (RR, 1.31; 95% CI, 1.04 to 1.65).

#### 3.4.3 Fetal stress

One study (31) reported that offspring of patients with BC may have a higher risk of fetal stress (SIR, 1.10; 95% CI, 1.01 to 1.20).

#### 3.4.4 Fetal demise

Three studies reported on fetal demise. One study (32) found no significant difference (OR, 0.75; 95% CI, 0.08 to 6.72). A second study (37) noted a perinatal mortality rate of 0/43 in the BC group and 0.98% in the healthy population. A third study (30) reported no difference in neonatal mortality within 27 days (OR, 1.36; 95% CI, 0.43 to 4.26).

#### 3.4.5 Live birth

Eight studies were included in the meta-analysis. Effect measures consisted of OR (two studies) (16, 17), RR (two studies) (18, 19), and RR based on binary data (four studies) (22, 26–28). With the exception of non-significant findings in a few reports, offspring of patients with BC showed a lower live birth rate (RR, 0.27; 95% CI, 0.11 to 0.70).

One study (20) reported a similar result (SBR, 0.43; 95% CI, 0.30 to 0.58).

#### 3.4.6 Apgar score

Two studies (28, 30) reported no statistical difference in Apgar scores below 7 (OR, 1.15; 95% CI, 0.75 to 1.78). However, one study (33) found that offspring born to patients with BC had a higher risk of low Apgar scores (RR, 1.7; 95% CI, 1.1 to 2.7).

#### 3.4.7 Fetal abnormalities

Eight studies were included in this meta-analysis. Effect measures included OR (five studies) (17, 28, 30, 32, 37) and RR (three studies) (34, 38, 40). Except for studies with non-significant findings, the overall risk of fetal abnormalities was higher among offspring of patients with BC (OR, 1.42; 95% CI, 1.04 to 1.94).

One study (33) found no difference in neonatal intensive care unit (NICU) admission (RR, 1.3; 95% CI, 0.8 to 2.2).

### 3.5 Pregnancy complications

A total of six studies were included in this analysis. No significant differences were found in the following complications: pre-eclampsia (OR, 0.69; 95% CI, 0.47 to 1.03 (17, 23, 30); SIR, 1.12; 95% CI, 0.85 to 1.47) (31), pregnancy-related bleeding (OR, 1.00; 95% CI, 0.52 to 1.93) (17, 28, 30), gestational diabetes (OR, 1.19; 95% CI, 0.76 to 1.86; SIR, 0.83; 95% CI, 0.66 to 1.04) (17, 30, 31), genitourinary infections (OR, 0.53; 95% CI, 0.19 to 1.46; SIR, 0.80; 95% CI, 0.45 to 1.40) (17, 31), known or suspected pelvic organ abnormalities (OR, 1.00; 95% CI, 0.41 to 2.47) (17), severe maternal morbidity based on Centers for Disease Control and Prevention (CDC) algorithm (OR, 1.61; 95% CI, 0.74 to 3.50) (32), pre-existing hypertension complicating pregnancy (SIR, 0.78; 95% CI, 0.43 to 1.41) (31), unspecified maternal hypertension (SIR, 0.98; 95% CI, 0.79 to 1.20) (31), gestational edema and proteinuria without hypertension (SIR, 0.81; 95% CI, 0.40 to 1.62) (31), and breast or lactation disorders related to childbirth (OR, 1.77; 95% CI, 0.68 to 4.62) (17).

One study (30) reported a lower rate of gestational hypertension in patients with BC (OR, 0.61; 95% CI, 0.42 to 0.90), while another (31) found no difference (SIR, 1.00; 95% CI, 0.77 to 1.30).

### 3.6 Subgroup analysis

Among the 26 included articles, four were incorporated into the subgroup analysis (**eFigure 5**, **Supplementary 4**).

#### 3.6.1 Cesarean delivery

Two articles (20, 33) were included in the subgroup analysis assessing cesarean delivery rates among patients with invasive BC. Compared with healthy populations, patients with invasive BC had a higher rate of cesarean delivery (RR, 1.17; 95% CI, 1.06 to 1.29).

One study (33) also reported that patients with ductal carcinoma *in situ* (DCIS) had a slightly increased risk of cesarean delivery (RR, 1.2; 95% CI, 1.0 to 1.4).

Additionally, two studies (20, 30) compared patients with BC who gave birth ≥5 years after diagnosis with healthy individuals. These patients showed a higher risk of cesarean delivery (RR, 1.20; 95% CI, 1.01 to 1.42; *p* = 0.037).

#### 3.6.2 Preterm birth

Two articles (20, 35) were included in the subgroup analysis to assess the effect of chemotherapy on preterm birth (PTB). No significant difference was observed in the risk of preterm birth between BC patients who received chemotherapy and healthy controls (RR, 1.59; 95% CI, 0.83 to 3.04). Similarly, no statistical difference was found between BC patients who did not receive chemotherapy and healthy populations (RR, 1.16; 95% CI, 0.90 to 1.49; *p* = 0.258).

Two other studies (20, 33) examined the association between invasive BC and preterm birth. Compared with healthy individuals, patients with invasive BC had a higher risk of preterm birth (RR, 1.30; 95% CI, 1.08 to 1.56). For patients with DCIS, there was no increased risk of preterm birth compared to healthy populations (RR, 0.9; 95% CI, 0.6 to 1.5) (33).

#### 3.6.3 Low birth weight at term (<2,500 g)

Two studies (20, 33) were included in the subgroup analysis assessing the risk of low birth weight at term in patients with invasive BC. Compared with healthy individuals, those with invasive BC had a higher risk (RR, 1.52; 95% CI, 1.26 to 1.83). In contrast, patients with DCIS showed no increased risk (RR, 1.2; 95% CI, 0.7 to 2.0) (33).

Two studies (20, 35) were also analyzed to examine the effect of chemotherapy. BC patients who underwent chemotherapy had a higher risk of low birth weight at term (RR, 1.62; 95% CI, 1.08 to 2.42), whereas those who did not receive chemotherapy showed no such risk (RR, 1.05; 95% CI, 0.77 to 1.43).

#### 3.6.4 Small for gestational age

Two studies (20, 35) explored the relationship between chemotherapy and small for gestational age (SGA). Compared with healthy populations, BC patients who had received chemotherapy had a higher risk of SGA (RR, 1.51; 95% CI, 1.22 to 1.88), while those who had not received chemotherapy did not show a higher risk (RR, 0.90; 95% CI, 0.64 to 1.25).

Two studies (20, 35) were also used to assess the impact of time between BC diagnosis and birth. BC patients who gave birth ≥5 years after diagnosis had a higher risk of SGA (RR, 1.66; 95% CI, 1.21 to 2.27), while those who gave birth within 5 years of diagnosis had no increased risk (RR, 0.90; 95% CI, 0.59 to 1.38) (20). In comparison to those who gave birth ≥5 years after diagnosis, patients who gave birth within 2 years of BC diagnosis had a higher risk of preterm birth (PR, 2.19; 95% CI, 1.31 to 3.67) (35).

## 4 Discussion

Our meta-analysis showed that patients with BC had lower pregnancy prevalence, lower completed pregnancy rate, lower childbirth rate, lower birth trauma rate, and higher rates of cesarean delivery and preterm birth (<37 weeks). Offspring born to patients with BC may face increased risks of very low birth weight (<1,500 g), low birth weight (<2,500 g), fetal abnormalities, and lower live birth rates. The systematic review further indicated that patients with BC may experience higher rates of intrapartum hemorrhage, induced delivery, spontaneous delivery, failed induction of labor, prolonged labor, fetal stress, and delivery complications, as well as a lower rate of full-term delivery and reduced incidence of gestational hypertension.

Two main reasons may explain the lower prevalence of pregnancy in patients with BC. The first is concern over a possible negative impact on cancer outcomes (41, 42). The second is that a history of BC and its treatment may lead to reduced fertility and diminished ovarian reserve (43, 44). Although cytotoxic drugs can significantly lower mortality in women with BC, they can also cause reproductive toxicity (45), making decreased pregnancy rates a likely challenge for survivors.

Induced delivery refers to the artificial initiation of labor after 12 weeks of gestation for maternal or fetal indications. Plasma prolactin levels, which increase during pregnancy, have been found to be positively associated with BC, particularly in estrogen receptor (ER)+/Progesterone Receptor (PR)+ and invasive types (46). A higher rate of induced delivery among BC patients may reflect the need to resume cancer treatment promptly or respond to poor fetal growth. Additionally, unsuccessful induction of labor, prolonged labor, and intrapartum hemorrhage can lead to excessive maternal fatigue and physical stress, raising the likelihood of fetal stress. Preterm birth may be influenced by cancer itself or by cancer-related fatigue, anxiety, and pain (47, 48). Similarly, the increased risks of low birth weight, very low birth weight, and lower live birth rates in offspring of BC patients may be connected to preterm delivery, induced labor, and failed labor induction.

Previous studies have reported that, in addition to increased risks of preterm birth and low birth weight, offspring born to patients with BC also faced a higher risk of being SGA (9). However, our findings did not support this result. This difference may be explained by the inclusion of a 2024 study (38), the exclusion of one earlier study (17), and the replacement of effect measures in two others (20, 35). The excluded study used inconsistent outcome definitions (suspected poor fetal growth vs. SGA), while the replacements retained the original RR values. In contrast to prior research (9), we found a significantly lower completed pregnancy rate (*p* < 0.05) and a higher rate of fetal abnormalities (*p* < 0.05), which were not statistically significant in previous analyses. This may be due to the larger number of articles included in our review.

High heterogeneity was observed for completed pregnancy, SGA, and live birth outcomes, mainly influenced by studies (22, 24, 25, 38) with narrow confidence intervals and extreme HR values, as shown in the sensitivity analyses (**eFigure 4**, **Supplementary 3**).

Subgroup analysis indicated that both invasive BC and DCIS were associated with higher cesarean delivery rates than those seen in healthy populations. This may reflect a preference among BC patients for cesarean delivery to avoid physiological stress during labor. Invasive BC, unlike DCIS, was associated with a higher risk of preterm birth. When evaluating the role of chemotherapy, we found no significant increase in preterm birth risk in BC patients regardless of chemotherapy exposure. However, some studies have suggested that specific chemotherapy regimens may lead to preterm birth by suppressing immune function and impairing the body’s ability to respond to infections, which could trigger early labor. This risk may vary depending on the timing between chemotherapy and delivery (49).

Regarding fetal outcomes, offspring born to BC patients who had received chemotherapy were more likely to have low birth weight at term (<2,500 g) and be SGA. In contrast, no such risk was observed among those born to BC patients who had not undergone chemotherapy. This difference could be linked to vascular damage to the placenta caused by chemotherapy and the immunosuppressive effects of treatment. These factors may compromise the intrauterine environment and contribute to fetal growth restriction (33).

One study indicated that women with ER-positive tumors were less likely to become pregnant within 5 years of diagnosis. However, by the 10-year mark, their cumulative childbirth rate was comparable to that of ER-negative BC patients (20). This may be due to the common recommendation for women with ER-positive tumors to undergo adjuvant endocrine therapy for at least 5 years, with pregnancy usually advised only after completing the treatment. Compared with healthy populations, BC patients with ER-positive tumors did not show increased rates of preterm birth, low birth weight (LBW), SGA, or cesarean delivery. In contrast, infants born to patients with ER-negative tumors had a higher risk of PTB (RR, 1.84; 95% CI, 1.11 to 3.06) and low birth weight (RR, 2.51; 95% CI, 1.53 to 4.12) (20). However, due to the limited availability of detailed data on BC subtypes and treatment regimens, results related to ER status, endocrine therapy, and other therapeutic approaches require further study for clarification.

Subgroup analyses also showed that BC patients with a diagnosis-to-childbirth interval of ≥5 years had a higher risk of cesarean delivery and SGA. Additionally, those who gave birth within 2 years of diagnosis faced increased SGA risk, while no such risk was observed in patients whose childbirth occurred less than 5 years post-diagnosis. Still, these findings should be interpreted with caution, as they are based on a limited number of outcomes and may be influenced by random variation.

## 5 Strengths and limitations

This study stands out for its thorough and systematic review approach. Original effect measures were retained to maintain the accuracy of the findings. However, several limitations should be noted. High heterogeneity was observed in some outcomes, and most of the included studies were retrospective in design. The number of eligible articles within various subgroups was small, and a lack of detailed data limited the ability to confirm subgroup-specific results. Therefore, important subgroup factors, such as the interval between cancer therapy and pregnancy (50), and tumor characteristics (51), were not analyzed separately.

## 6 Conclusion

This study provides updated and reliable evidence-based insights into the reproductive outcomes of pregnancies in women with a history of BC, which is essential for fertility counseling. Given the potential for unsatisfactory outcomes, patients with BC and their healthcare providers should make well-informed decisions about pregnancy timing and management, and appropriate evaluations should be carried out with care.

## Data Availability

The original contributions presented in the study are included in the article/**Supplementary Material**. Further inquiries can be directed to the corresponding author.

## Supplementary material

The MOOSE Checklist and PRISMA Checklist are in **Supplementary 5** and **Supplementary 6**, respectively.

## References

[1] Bray F Laversanne M Sung H Ferlay J Siegel RL Soerjomataram I . Global cancer statistics 2022: GLOBOCAN estimates of incidence and mortality worldwide for 36 cancers in 185 countries. CA Cancer J Clin. (2024) 74:229–63. doi: 10.3322/caac.21834, PMID: 38572751

[2] Londero AP Bertozzi S Xholli A Cedolini C Cagnacci A . Breast cancer and the steadily increasing maternal age: are they colliding? BMC Womens Health. (2024) 24:286. doi: 10.1186/s12905-024-03138-4, PMID: 38745181 PMC11092140

[3] Poorvu PD Hu J Zheng Y Gelber SI Ruddy KJ Tamimi RM . Treatment-related amenorrhea in a modern, prospective cohort study of young women with breast cancer. NPJ Breast Cancer. (2021) 7:99. doi: 10.1038/s41523-021-00307-8, PMID: 34315890 PMC8316568

[4] Hickey M Basu P Sassarini J Stegmann ME Weiderpass E Nakawala Chilowa K . Managing menopause after cancer. Lancet. (2024) 403:984–96. doi: 10.1016/s0140-6736(23)02802-7, PMID: 38458217

[5] Goldberg C Greenberg MR Noveihed A Agrawal L Omene C Toppmeyer D . Ovarian suppression: early menopause, late effects. Curr Oncol Rep. (2024) 26:427–38. doi: 10.1007/s11912-023-01491-5, PMID: 38305992

[6] Tanaka Y Amano T Nakamura A Yoshino F Takebayashi A Takahashi A . Rapamycin prevents cyclophosphamide-induced ovarian follicular loss and potentially inhibits tumour proliferation in a breast cancer xenograft mouse model. Hum Reprod. (2024) 39:1519–32. doi: 10.1093/humrep/deae085, PMID: 38734930 PMC11759105

[7] Hu L Xu B Chau PH Lok KYW Kwok JYY Choi EPH . Reproductive concerns among young adult women with breast cancer: A systematic review and meta-analysis. Psychooncology. (2024) 33:e9304. doi: 10.1002/pon.9304, PMID: 39160674

[8] Lambertini M Blondeaux E Agostinetto E Hamy AS Kim HJ Di Meglio A . Pregnancy after breast cancer in young BRCA carriers: an international hospital-based cohort study. Jama. (2024) 331:49–59. doi: 10.1001/jama.2023.25463, PMID: 38059899 PMC10704340

[9] Lambertini M Blondeaux E Bruzzone M Perachino M Anderson RA de Azambuja E . Pregnancy after breast cancer: A systematic review and meta-analysis. J Clin Oncol. (2021) 39:3293–305. doi: 10.1200/jco.21.00535, PMID: 34197218

[10] Moher D Liberati A Tetzlaff J Altman DG . Preferred reporting items for systematic reviews and meta-analyses: the PRISMA statement. Bmj. (2009) 339:b2535. doi: 10.1136/bmj.b2535, PMID: 19622551 PMC2714657

[11] Brooke BS Schwartz TA Pawlik TM . MOOSE reporting guidelines for meta-analyses of observational studies. JAMA Surg. (2021) 156:787–8. doi: 10.1001/jamasurg.2021.0522, PMID: 33825847

[12] Stang A . Critical evaluation of the Newcastle-Ottawa scale for the assessment of the quality of nonrandomized studies in meta-analyses. Eur J Epidemiol. (2010) 25:603–5. doi: 10.1007/s10654-010-9491-z, PMID: 20652370

[13] Fahmy O Fahmy UA Alhakamy NA Khairul-Asri MG . Single-port versus multiple-port robot-assisted radical prostatectomy: A systematic review and meta-analysis. J Clin Med. (2021) 10:5723. doi: 10.3390/jcm10245723, PMID: 34945018 PMC8703720

[14] Anderson RA Brewster DH Wood R Nowell S Fischbacher C Kelsey TW . The impact of cancer on subsequent chance of pregnancy: a population-based analysis. Hum Reprod. (2018) 33:1281–90. doi: 10.1093/humrep/dey216, PMID: 29912328 PMC6012597

[15] Stensheim H Cvancarova M Møller B Fosså SD . Pregnancy after adolescent and adult cancer: a population-based matched cohort study. Int J Cancer. (2011) 129:1225–36. doi: 10.1002/ijc.26045, PMID: 21387311

[16] Luke B Brown MB Missmer SA Spector LG Leach RE Williams M . Assisted reproductive technology use and outcomes among women with a history of cancer. Hum Reprod. (2016) 31:183–9. doi: 10.1093/humrep/dev288, PMID: 26577302 PMC4677965

[17] Jacob L Kalder M Arabin B Kostev K . Impact of prior breast cancer on mode of delivery and pregnancy-associated disorders: a retrospective analysis of subsequent pregnancy outcomes. J Cancer Res Clin Oncol. (2017) 143:1069–74. doi: 10.1007/s00432-017-2352-3, PMID: 28220257 PMC11818950

[18] Garg D Meeks HD Johnstone E Berga SL Smith KR Hotaling J . Cancer treatment is associated with a measurable decrease in live births in a large, population-based study. F S Rep. (2021) 2:462–7. doi: 10.1016/j.xfre.2021.08.004, PMID: 34934988 PMC8655402

[19] Madanat LM Malila N Dyba T Hakulinen T Sankila R Boice JD Jr. . Probability of parenthood after early onset cancer: a population-based study. Int J Cancer. (2008) 123:2891–8. doi: 10.1002/ijc.23842, PMID: 18798259 PMC2730156

[20] Anderson C Engel SM Anders CK Nichols HB . Live birth outcomes after adolescent and young adult breast cancer. Int J Cancer. (2018) 142:1994–2002. doi: 10.1002/ijc.31227, PMID: 29266267 PMC5867233

[21] Hartman M Liu J Czene K Miao H Chia KS Salim A . Birth rates among female cancer survivors: A population-based cohort study in Sweden. Cancer. (2013) 119:1892–9. doi: 10.1002/cncr.27929, PMID: 23436251

[22] Anderson RA Kelsey TW Morrison DS Wallace WHB . Family size and duration of fertility in female cancer survivors: a population-based analysis. Fertility Sterility. (2022) 117:387–95. doi: 10.1016/j.fertnstert.2021.11.011, PMID: 34933761 PMC8865032

[23] Lee HM Kim BW Park S Park S Lee JE Choi YJ . Childbirth in young Korean women with previously treated breast cancer: The SMARTSHIP study. Breast Cancer Res Treat. (2019) 176:419–27. doi: 10.1007/s10549-019-05244-6, PMID: 31020470

[24] Baxter NN Sutradhar R DelGuidice ME Forbes S Paszat LF Wilton AS . A population-based study of rates of childbirth in recurrence-free female young adult survivors of non-gynecologic Malignancies. BMC Cancer. (2013) 13:30. doi: 10.1186/1471-2407-13-30, PMID: 23343211 PMC3605316

[25] Rushton M Pudwell J Wei X Powell M Richardson H Velez MP . Reproductive outcomes in young breast cancer survivors treated (15-39) in ontario, Canada. Curr Oncol. (2022) 29:8591–9. doi: 10.3390/curroncol29110677, PMID: 36421330 PMC9689574

[26] Pereira N Hancock K Cordeiro CN Lekovich JP Schattman GL Rosenwaks Z . Comparison of ovarian stimulation response in patients with breast cancer undergoing ovarian stimulation with letrozole and gonadotropins to patients undergoing ovarian stimulation with gonadotropins alone for elective cryopreservation of oocytes†. Gynecol Endocrinol. (2016) 32:823–6. doi: 10.1080/09513590.2016.1177013, PMID: 27114051

[27] Ma KK Preusse CJ Stevenson PA Winget VL McDougall JA Li CI . Obstetric outcomes in young women with breast cancer: prior, postpartum, and subsequent pregnancies. Am J Perinatol. (2020) 37:370–4. doi: 10.1055/s-0039-1678603, PMID: 30726999

[28] Dalberg K Eriksson J Holmberg L . Birth outcome in women with previously treated breast cancer–a population-based cohort study from Sweden. PloS Med. (2006) 3:e336. doi: 10.1371/journal.pmed.0030336, PMID: 16968117 PMC1564170

[29] Velentgas P Daling JR Malone KE Weiss NS Williams MA Self SG . Pregnancy after breast carcinoma: outcomes and influence on mortality. Cancer. (1999) 85:2424–32. doi: 10.1002/(SICI)1097-0142(19990601)85:11<2424::AID-CNCR17>3.0.CO;2-4, PMID: 10357413

[30] Gkekos L Johansson ALV Rodriguez-Wallberg KA Fredriksson I Lundberg FE . Obstetric and perinatal outcomes in women with previous breast cancer: a nationwide study of singleton births 1973-2017. Hum Reprod Open. (2024) 2024:hoae027. doi: 10.1093/hropen/hoae027, PMID: 38784055 PMC11112047

[31] Sunguc C Winter DL Heymer EJ Rudge G Polanco A Birchenall KA . Risks of adverse obstetric outcomes among female survivors of adolescent and young adult cancer in England (TYACSS): a population-based, retrospective cohort study. Lancet Oncol. (2024) 25:1080–91. doi: 10.1016/s1470-2045(24)00269-9, PMID: 38944050

[32] Jorgensen K Nitecki R Nichols HB Fu S Wu CF Melamed A . Obstetric and neonatal outcomes 1 or more years after a diagnosis of breast cancer. Obstet Gynecol. (2022) 140:939–49. doi: 10.1097/aog.0000000000004936, PMID: 36357983 PMC9712170

[33] Hartnett KP Ward KC Kramer MR Lash TL Mertens AC Spencer JB . The risk of preterm birth and growth restriction in pregnancy after cancer. Int J Cancer. (2017) 141:2187–96. doi: 10.1002/ijc.30914, PMID: 28836277 PMC5766343

[34] Langagergaard V Gislum M Skriver MV Nørgård B Lash TL Rothman KJ . Birth outcome in women with breast cancer. Br J Cancer. (2006) 94:142–6. doi: 10.1038/sj.bjc.6602878, PMID: 16306874 PMC2361078

[35] Black KZ Nichols HB Eng E Rowley DL . Prevalence of preterm, low birthweight, and small for gestational age delivery after breast cancer diagnosis: a population-based study. Breast Cancer Res. (2017) 19:11. doi: 10.1186/s13058-017-0803-z, PMID: 28143580 PMC5282806

[36] Anderson C Baggett CD Engel SM Getahun D Cannizzaro NT Mitra S . Risk of adverse birth outcomes after adolescent and young adult cancer. JNCI Cancer Spectr. (2023) 8:pkad106. doi: 10.1093/jncics/pkad106, PMID: 38127994 PMC10868397

[37] Stensheim H Klungsøyr K Skjaerven R Grotmol T Fosså SD . Birth outcomes among offspring of adult cancer survivors: a population-based study. Int J Cancer. (2013) 133:2696–705. doi: 10.1002/ijc.28292, PMID: 23729011

[38] Anderson C Baggett CD Engel SM Getahun D Cannizzaro NT Mitra S . Risk of adverse birth outcomes after adolescent and young adult cancer. JNCI Cancer Spectr. (2024) 8:pkad106. doi: 10.1093/jncics/pkad106, PMID: 38127994 PMC10868397

[39] Verkooijen HM Ang JX Liu J Czene K Salim A Hartman M . Mortality among offspring of women diagnosed with cancer: a population-based cohort study. Int J Cancer. (2013) 132:2432–8. doi: 10.1002/ijc.27899, PMID: 23047289

[40] Auger N Maniraho A Ayoub A Arbour L . Association of maternal cancer with congenital anomalies in offspring. Paediatr Perinat Epidemiol. (2023) 38:121–9. doi: 10.1111/ppe.13031, PMID: 38112586

[41] Kuswanto CN Sharp J Stafford L Schofield P . Fear of cancer recurrence as a pathway from fatigue to psychological distress in mothers who are breast cancer survivors. Stress Health. (2023) 39:197–208. doi: 10.1002/smi.3180, PMID: 35751136 PMC10084015

[42] Xu W Liu X Zhang C Zhu L Zhao Y Liao C . Post-treatment experiences of reproductive concerns among young breast cancer survivors: A descriptive phenomenological study. Asian Nurs Res (Korean Soc Nurs Sci). (2024) 18:331–40. doi: 10.1016/j.anr.2024.09.003, PMID: 39255899

[43] Yildiz S Bildik G Benlioglu C Turan V Dilege E Ozel M . Breast cancer treatment and ovarian function. Reprod BioMed Online. (2023) 46:313–31. doi: 10.1016/j.rbmo.2022.09.014, PMID: 36400663

[44] Chitoran E Rotaru V Mitroiu MN Durdu CE Bohiltea RE Ionescu SO . Navigating fertility preservation options in gynecological cancers: A comprehensive review. Cancers (Basel). (2024) 16:2214. doi: 10.3390/cancers16122214, PMID: 38927920 PMC11201795

[45] Xu Z Takahashi N Harada M Kunitomi C Kusamoto A Koike H . The role of cellular senescence in cyclophosphamide-induced primary ovarian insufficiency. Int J Mol Sci. (2023) 24:17193. doi: 10.3390/ijms242417193, PMID: 38139022 PMC10743614

[46] Aranha AF Dos Anjos LG Turri JAO Simões RS Maciel GAR Baracat EC . Impact of the prolactin levels in breast cancer: a systematic review and meta-analysis. Gynecol Endocrinol. (2022) 38:385–90. doi: 10.1080/09513590.2022.2047173, PMID: 35266411

[47] Becker M Mayo JA Phogat NK Quaintance CC Laborde A King L . Deleterious and protective psychosocial and stress-related factors predict risk of spontaneous preterm birth. Am J Perinatol. (2023) 40:74–88. doi: 10.1055/s-0041-1729162, PMID: 34015838 PMC11036409

[48] Quenby S Gallos ID Dhillon-Smith RK Podesek M Stephenson MD Fisher J . Miscarriage matters: the epidemiological, physical, psychological, and economic costs of early pregnancy loss. Lancet. (2021) 397:1658–67. doi: 10.1016/s0140-6736(21)00682-6, PMID: 33915094

[49] Helmo FR Alves EAR Moreira RAA Severino VO Rocha LP Monteiro M . Intrauterine infection, immune system and premature birth. J Matern Fetal Neonatal Med. (2018) 31:1227–33. doi: 10.1080/14767058.2017.1311318, PMID: 28423971

[50] Hartnett KP Mertens AC Kramer MR Lash TL Spencer JB Ward KC . Pregnancy after cancer: Does timing of conception affect infant health? Cancer. (2018) 124:4401–7. doi: 10.1002/cncr.31732, PMID: 30403424 PMC7886368

[51] Caswell-Jin JL Sun LP Munoz D Lu Y Li Y Huang H . Analysis of breast cancer mortality in the US-1975 to 2019. Jama. (2024) 331:233–41. doi: 10.1001/jama.2023.25881, PMID: 38227031 PMC10792466

