# Supplementary material for: Reproductive outcomes of pregnancy after breast cancer: an updated systematic review and meta-analysis
Source: Front Oncol. 2025 Sep 26;15:1569109. doi: 10.3389/fonc.2025.1569109 (PMC12510861; doi:10.3389/fonc.2025.1569109)
Supplement: Supplementary file 1 — The MOOSE Checklist and PRISMA Checklist are in Supplementary 5 and Supplementary 6 , respectively. [file SupplementaryFile1.docx]

**Search Strategies**

**1 PubMed**

((("Breast Neoplasms"[Mesh]) OR ((Breast Neoplasm [Title/Abstract]) OR (Breast Neoplasms [Title/Abstract]) OR (Neoplasm, Breast[Title/Abstract]) OR (Neoplasms, Breast[Title/Abstract]) OR (Breast Tumors[Title/Abstract]) OR (Breast Tumor[Title/Abstract]) OR (Tumor, Breast[Title/Abstract]) OR (Tumors, Breast[Title/Abstract]) OR (Breast Cancer[Title/Abstract]) OR (Cancer, Breast[Title/Abstract]) OR (Mammary Cancer[Title/Abstract]) OR (Cancer, Mammary[Title/Abstract]) OR (Cancers, Mammary[Title/Abstract]) OR (Mammary Cancers[Title/Abstract]) OR (Malignant Neoplasm of Breast[Title/Abstract]) OR (Breast Malignant Neoplasm[Title/Abstract]) OR (Breast Malignant Neoplasms[Title/Abstract]) OR (Malignant Tumor of Breast[Title/Abstract]) OR (Breast Malignancy[Title/Abstract]) OR (Breast Malign*[Title/Abstract]) OR (Breast Malignant Tumor[Title/Abstract]) OR (Breast Malignant Tumors[Title/Abstract]) OR (Cancer of Breast[Title/Abstract]) OR (Cancer of the Breast[Title/Abstract]) OR (Mammary Carcinoma, Human[Title/Abstract]) OR (Carcinoma, Human Mammary[Title/Abstract]) OR (Carcinomas, Human Mammary[Title/Abstract]) OR (Human Mammary Carcinomas[Title/Abstract]) OR (Mammary Carcinomas, Human[Title/Abstract]) OR (Human Mammary Carcinoma[Title/Abstract]) OR (Mammary Neoplasms, Human[Title/Abstract]) OR (Human Mammary Neoplasm[Title/Abstract]) OR (Human Mammary Neoplasms[Title/Abstract]) OR (Neoplasm, Human Mammary[Title/Abstract]) OR (Neoplasms, Human Mammary[Title/Abstract]) OR (Mammary Neoplasm, Human[Title/Abstract]) OR (Breast Carcinoma[Title/Abstract]) OR (Breast Carcinomas[Title/Abstract]) OR (Carcinoma, Breast[Title/Abstract]) OR (Carcinomas, Breast[Title/Abstract]))) AND (((((("Pregnancy"[Mesh]) OR ("Fertilization"[Mesh])) OR ("Parturition"[Mesh])) OR ("Fertility"[Mesh])) OR ("Obstetrics"[Mesh])) OR ((Pregnancy[Title/Abstract]) OR (Pregnancies[Title/Abstract]) OR (Gestation[Title/Abstract]) OR (Pregnant[Title/Abstract]) OR (Fertilization[Title/Abstract]) OR (Fertilizations[Title/Abstract]) OR (Fertilization, Delayed[Title/Abstract]) OR (Delayed Fertilization[Title/Abstract]) OR (Delayed Fertilizations[Title/Abstract]) OR (Fertilizations, Delayed[Title/Abstract]) OR (Fertilization, Polyspermic[Title/Abstract]) OR (Fertilizations, Polyspermic[Title/Abstract]) OR (Polyspermic Fertilization[Title/Abstract]) OR (Polyspermic Fertilizations[Title/Abstract]) OR (Conception[Title/Abstract]) OR (Conceptions[Title/Abstract]) OR (Conceiving[Title/Abstract]) OR (Parturition[Title/Abstract]) OR (Parturitions[Title/Abstract]) OR (Birth[Title/Abstract]) OR (Births[Title/Abstract]) OR (Childbirth[Title/Abstract]) OR (Childbirths[Title/Abstract]) OR (Fertility[Title/Abstract]) OR (Fecundability[Title/Abstract]) OR (Fecundity[Title/Abstract]) OR (Differential Fertility[Title/Abstract]) OR (Fertility, Differential[Title/Abstract]) OR (Fertility Determinants[Title/Abstract]) OR (Determinant, Fertility[Title/Abstract]) OR (Determinants, Fertility[Title/Abstract]) OR (Fertility Determinant[Title/Abstract]) OR (Subfecundity[Title/Abstract]) OR (Fertility Preferences[Title/Abstract]) OR (Fertility Preference[Title/Abstract]) OR (Preference, Fertility[Title/Abstract]) OR (Preferences, Fertility[Title/Abstract]) OR (Fertility, Below Replacement[Title/Abstract]) OR (Below Replacement Fertility[Title/Abstract]) OR (Marital Fertility[Title/Abstract]) OR (Fertility, Marital[Title/Abstract]) OR (Natural Fertility[Title/Abstract]) OR (Fertility, Natural[Title/Abstract]) OR (World Fertility Survey[Title/Abstract]) OR (Fertility Survey, World[Title/Abstract]) OR (Fertility Surveys, World[Title/Abstract]) OR (Survey, World Fertility[Title/Abstract]) OR (Surveys, World Fertility[Title/Abstract]) OR (World Fertility Surveys[Title/Abstract]) OR (Fertility Incentives[Title/Abstract]) OR (Fertility Incentive[Title/Abstract]) OR (Obstetrics[Title/Abstract]) OR (Obstetrical outcomes[Title/Abstract])))) AND ((((("Cohort Studies"[Mesh]) OR ("Case-Control Studies"[Mesh])) OR ("Clinical Trial" [Publication Type])) OR ("Randomized Controlled Trial" [Publication Type])) OR ((cohort studies[Title/Abstract]) OR (case-control studies[Title/Abstract]) OR (comparative study[Title/Abstract]) OR (cohort*[Title/Abstract]) OR (compared[Title/Abstract]) OR (case control[Title/Abstract]) OR (case series[Title/Abstract]) OR (randomized controlled trial[Title/Abstract]) OR (controlled clinical trial[Title/Abstract]) OR (randomized[Title/Abstract]) OR (placebo[Title/Abstract]) OR (drug therapy[Title/Abstract]) OR (randomly[Title/Abstract]) OR (clinical trial[Title/Abstract]))) Filters: from 1000/1/1 - 2024/09/03

**2 Embase**

#17 #3 AND #10 AND #16 AND [1900-2024]/py 7534

#16 #11 OR #12 OR #13 OR #14 OR #15 9,867,951

#15 'cohort studies':ab,ti OR 'case-control studies':ab,ti OR 'comparative study':ab,ti OR 'cohort*':ab,ti OR 'compared':ab,ti OR 'case control':ab,ti OR 'case series':ab,ti OR 'randomized controlled trial':ab,ti OR 'controlled clinical trial':ab,ti OR 'randomized':ab,ti OR 'placebo':ab,ti OR 'drug therapy':ab,ti OR 'randomly':ab,ti OR 'clinical trial':ab,ti 8,923,025

#14 'randomized controlled trial'/exp 840,502

#13 'clinical trial'/exp 1,946,125

#12 'case control study'/exp 238,989

#11 'cohort analysis'/exp 1,205,467

#10 #4 OR #5 OR #6 OR #7 OR #8 OR #9 1,836,821

#9 'pregnancy':ab,ti OR 'pregnancies':ab,ti OR 'gestation':ab,ti OR 'pregnant':ab,ti OR 'fertilization':ab,ti OR 'fertilizations':ab,ti OR 'fertilization, delayed':ab,ti OR 'delayed fertilization':ab,ti OR 'delayed fertilizations':ab,ti OR 'fertilizations, delayed':ab,ti OR 'fertilization, polyspermic':ab,ti OR 'fertilizations, polyspermic':ab,ti OR 'polyspermic fertilization':ab,ti OR 'polyspermic fertilizations':ab,ti OR 'conception':ab,ti OR 'conceptions':ab,ti OR 'conceiving':ab,ti OR 'parturition':ab,ti OR 'parturitions':ab,ti OR 'birth':ab,ti OR 'births':ab,ti OR 'childbirth':ab,ti OR 'childbirths':ab,ti OR 'fertility':ab,ti OR 'fecundability':ab,ti OR 'fecundity':ab,ti OR 'differential fertility':ab,ti OR 'fertility, differential':ab,ti OR 'fertility determinants':ab,ti OR 'determinant, fertility':ab,ti OR 'determinants, fertility':ab,ti OR 'fertility determinant':ab,ti OR 'subfecundity':ab,ti OR 'fertility preferences':ab,ti OR 'fertility preference':ab,ti OR 'preference, fertility':ab,ti OR 'preferences, fertility':ab,ti OR 'fertility, below replacement':ab,ti OR 'below replacement fertility':ab,ti OR 'marital fertility':ab,ti OR 'fertility, marital':ab,ti OR 'natural fertility':ab,ti OR 'fertility, natural':ab,ti OR 'world fertility survey':ab,ti OR 'fertility survey, world':ab,ti OR 'fertility surveys, world':ab,ti OR 'survey, world fertility':ab,ti OR 'surveys, world fertility':ab,ti OR 'world fertility surveys':ab,ti OR 'fertility incentives':ab,ti OR 'fertility incentive':ab,ti OR 'obstetrics':ab,ti OR 'obstetrical outcomes':ab,ti 1,489,214

#8 'obstetrics'/exp 47,723

#7 'fertility'/exp 100,483

#6 'birth'/exp 34,092

#5 'fertilization'/exp 42,020

#4 'pregnancy'/exp 916,396

#3 #1 OR #2 735,912

#2 'breast neoplasm':ab,ti OR 'breast neoplasms':ab,ti OR 'neoplasm, breast':ab,ti OR 'neoplasms, breast':ab,ti OR 'breast tumors':ab,ti OR 'breast tumor':ab,ti OR 'tumor, breast':ab,ti OR 'tumors, breast':ab,ti OR 'breast cancer':ab,ti OR 'cancer, breast':ab,ti OR 'mammary cancer':ab,ti OR 'cancer, mammary':ab,ti OR 'cancers, mammary':ab,ti OR 'mammary cancers':ab,ti OR 'malignant neoplasm of breast':ab,ti OR 'breast malignant neoplasm':ab,ti OR 'breast malignant neoplasms':ab,ti OR 'malignant tumor of breast':ab,ti OR 'breast malignancy':ab,ti OR 'breast malign*':ab,ti OR 'breast malignant tumor':ab,ti OR 'breast malignant tumors':ab,ti OR 'cancer of breast':ab,ti OR 'cancer of the breast':ab,ti OR 'mammary carcinoma, human':ab,ti OR 'carcinoma, human mammary':ab,ti OR 'carcinomas, human mammary':ab,ti OR 'human mammary carcinomas':ab,ti OR 'mammary carcinomas, human':ab,ti OR 'human mammary carcinoma':ab,ti OR 'mammary neoplasms, human':ab,ti OR 'human mammary neoplasm':ab,ti OR 'human mammary neoplasms':ab,ti OR 'neoplasm, human mammary':ab,ti OR 'neoplasms, human mammary':ab,ti OR 'mammary neoplasm, human':ab,ti OR 'breast carcinoma':ab,ti OR 'breast carcinomas':ab,ti OR 'carcinoma, breast':ab,ti OR 'carcinomas, breast':ab,ti 560130

#1 'breast cancer'/exp 619,793

**3 Cochrane**

ID Search Hits

#1 MeSH descriptor: [Breast Neoplasms] explode all trees 20444

#2 (Breast Neoplasm):ti,ab,kw OR (Breast Neoplasms):ti,ab,kw OR (Neoplasm, Breast):ti,ab,kw OR (Neoplasms, Breast):ti,ab,kw OR (Breast Tumors):ti,ab,kw OR (Breast Tumor):ti,ab,kw OR (Tumor, Breast):ti,ab,kw OR (Tumors, Breast):ti,ab,kw OR (Breast Cancer):ti,ab,kw OR (Cancer, Breast):ti,ab,kw OR (Mammary Cancer):ti,ab,kw OR (Cancer, Mammary):ti,ab,kw OR (Cancers, Mammary):ti,ab,kw OR (Mammary Cancers):ti,ab,kw OR (Malignant Neoplasm of Breast):ti,ab,kw OR (Breast Malignant Neoplasm):ti,ab,kw OR (Breast Malignant Neoplasms):ti,ab,kw OR (Malignant Tumor of Breast):ti,ab,kw OR (Breast Malignancy):ti,ab,kw OR (Breast Malign*):ti,ab,kw OR (Breast Malignant Tumor):ti,ab,kw OR (Breast Malignant Tumors):ti,ab,kw OR (Cancer of Breast):ti,ab,kw OR (Cancer of the Breast):ti,ab,kw OR (Mammary Carcinoma, Human):ti,ab,kw OR (Carcinoma, Human Mammary):ti,ab,kw OR (Carcinomas, Human Mammary):ti,ab,kw OR (Human Mammary Carcinomas):ti,ab,kw OR (Mammary Carcinomas, Human):ti,ab,kw OR (Human Mammary Carcinoma):ti,ab,kw OR (Mammary Neoplasms, Human):ti,ab,kw OR (Human Mammary Neoplasm):ti,ab,kw OR (Human Mammary Neoplasms):ti,ab,kw OR (Neoplasm, Human Mammary):ti,ab,kw OR (Neoplasms, Human Mammary):ti,ab,kw OR (Mammary Neoplasm, Human):ti,ab,kw OR (Breast Carcinoma):ti,ab,kw OR (Breast Carcinomas):ti,ab,kw OR (Carcinoma, Breast):ti,ab,kw OR (Carcinomas, Breast):ti,ab,kw 49237

#3 #1 OR #2 49237

#4 MeSH descriptor: [Pregnancy] explode all trees 34383

#5 MeSH descriptor: [Parturition] explode all trees 911

#6 MeSH descriptor: [Obstetrics] explode all trees 340

#7 (Pregnancy):ti,ab,kw OR (Pregnancies):ti,ab,kw OR (Gestation):ti,ab,kw OR (Pregnant):ti,ab,kw OR (Fertilization):ti,ab,kw OR (Fertilizations):ti,ab,kw OR (Fertilization, Delayed):ti,ab,kw OR (Delayed Fertilization):ti,ab,kw OR (Delayed Fertilizations):ti,ab,kw OR (Fertilizations, Delayed):ti,ab,kw OR (Fertilization, Polyspermic):ti,ab,kw OR (Fertilizations, Polyspermic):ti,ab,kw OR (Polyspermic Fertilization):ti,ab,kw OR (Polyspermic Fertilizations):ti,ab,kw OR (Conception):ti,ab,kw OR (Conceptions):ti,ab,kw OR (Conceiving):ti,ab,kw OR (Parturition):ti,ab,kw OR (Parturitions):ti,ab,kw OR (Birth):ti,ab,kw OR (Births):ti,ab,kw OR (Childbirth):ti,ab,kw OR (Childbirths):ti,ab,kw OR (Fertility):ti,ab,kw OR (Fecundability):ti,ab,kw OR (Fecundity):ti,ab,kw OR (Differential Fertility):ti,ab,kw OR (Fertility, Differential):ti,ab,kw OR (Fertility Determinants):ti,ab,kw OR (Determinant, Fertility):ti,ab,kw OR (Determinants, Fertility):ti,ab,kw OR (Fertility Determinant):ti,ab,kw OR (Subfecundity):ti,ab,kw OR (Fertility Preferences):ti,ab,kw OR (Fertility Preference):ti,ab,kw OR (Preference, Fertility):ti,ab,kw OR (Preferences, Fertility):ti,ab,kw OR (Fertility, Below Replacement):ti,ab,kw OR (Below Replacement Fertility):ti,ab,kw OR (Marital Fertility):ti,ab,kw OR (Fertility, Marital):ti,ab,kw OR (Natural Fertility):ti,ab,kw OR (Fertility, Natural):ti,ab,kw OR (World Fertility Survey):ti,ab,kw OR (Fertility Survey, World):ti,ab,kw OR (Fertility Surveys, World):ti,ab,kw OR (Survey, World Fertility):ti,ab,kw OR (Surveys, World Fertility):ti,ab,kw OR (World Fertility Surveys):ti,ab,kw OR (Fertility Incentives):ti,ab,kw OR (Fertility Incentive):ti,ab,kw OR (Obstetrics):ti,ab,kw OR (Obstetrical outcomes):ti,ab,kw 120072

#8 #4 OR #5 OR #6 OR #7 120248

#9 #3 AND #8 1073

time: Start year-Present, search date:2024-09-03

**4 Web of Science-5337**

# Database: Web of Science Core Collection

# Searches:

1: TS=(Breast Neoplasm OR Breast Neoplasms OR Neoplasm, Breast OR Neoplasms, Breast OR Breast Tumors OR Breast Tumor OR Tumor, Breast OR Tumors, Breast OR Breast Cancer OR Cancer, Breast OR Mammary Cancer OR Cancer, Mammary OR Cancers, Mammary OR Mammary Cancers OR Malignant Neoplasm of Breast OR Breast Malignant Neoplasm OR Breast Malignant Neoplasms OR Malignant Tumor of Breast OR Breast Malignancy OR Breast Malign* OR Breast Malignant Tumor OR Breast Malignant Tumors OR Cancer of Breast OR Cancer of the Breast OR Mammary Carcinoma, Human OR Carcinoma, Human Mammary OR Carcinomas, Human Mammary OR Human Mammary Carcinomas OR Mammary Carcinomas, Human OR Human Mammary Carcinoma OR Mammary Neoplasms, Human OR Human Mammary Neoplasm OR Human Mammary Neoplasms OR Neoplasm, Human Mammary OR Neoplasms, Human Mammary OR Mammary Neoplasm, Human OR Breast Carcinoma OR Breast Carcinomas OR Carcinoma, Breast OR Carcinomas, Breast) Timespan: 1900-01-01 to 2024-09-03 Results: 577973

2: TS=(Pregnancy OR Pregnancies OR Gestation OR Pregnant OR Fertilization OR Fertilizations OR Fertilization, Delayed OR Delayed Fertilization OR Delayed Fertilizations OR Fertilizations, Delayed OR Fertilization, Polyspermic OR Fertilizations, Polyspermic OR Polyspermic Fertilization OR Polyspermic Fertilizations OR Conception OR Conceptions OR Conceiving OR Parturition OR Parturitions OR Birth OR Births OR Childbirth OR Childbirths OR Fertility OR Fecundability OR Fecundity OR Differential Fertility OR Fertility, Differential OR Fertility Determinants OR Determinant, Fertility OR Determinants, Fertility OR Fertility Determinant OR Subfecundity OR Fertility Preferences OR Fertility Preference OR Preference, Fertility OR Preferences, Fertility OR Fertility, Below Replacement OR Below Replacement Fertility OR Marital Fertility OR Fertility, Marital OR Natural Fertility OR Fertility, Natural OR World Fertility Survey OR Fertility Survey, World OR Fertility Surveys, World OR Survey, World Fertility OR Surveys, World Fertility OR World Fertility Surveys OR Fertility Incentives OR Fertility Incentive OR Obstetrics OR Obstetrical outcomes)

Timespan: 1900-01-01 to 2024-09-03 Results: 933,394

3: TS=(cohort studies OR case-control studies OR comparative study OR cohort* OR compared OR case control OR case series OR randomized controlled trial OR controlled clinical trial OR randomized OR placebo OR drug therapy OR randomly OR clinical trial)

Timespan: 1900-01-01 to 2024-09-03

4: #1 AND #2 AND #3 Timespan: 1900-01-01 to 2024-09-03 Results: 5,337

**5 Science Direct**

Year: 1900-2024

Title, abstract, keywords: (breast cancer OR breast neoplasm OR breast tumor OR breast carcinoma OR breast malignancy) AND (pregnancy OR gestation OR fertility OR parturition)

**6 Scopus**

( TITLE-ABS-KEY ( "Breast Neoplasm" ) OR TITLE-ABS-KEY ( "Breast Neoplasms" ) OR TITLE-ABS-KEY ( "Neoplasm, Breast" ) OR TITLE-ABS-KEY ( "Neoplasms, Breast" ) OR TITLE-ABS-KEY ( "Breast Tumors" ) OR TITLE-ABS-KEY ( "Breast Tumor" ) OR TITLE-ABS-KEY ( "Tumor, Breast" ) OR TITLE-ABS-KEY ( "Tumors, Breast" ) OR TITLE-ABS-KEY ( "Breast Cancer" ) OR TITLE-ABS-KEY ( "Cancer, Breast" ) OR TITLE-ABS-KEY ( "Mammary Cancer" ) OR TITLE-ABS-KEY ( "Cancer, Mammary" ) OR TITLE-ABS-KEY ( "Cancers, Mammary" ) OR TITLE-ABS-KEY ( "Mammary Cancers" ) OR TITLE-ABS-KEY ( "Malignant Neoplasm of Breast" ) OR TITLE-ABS-KEY ( "Breast Malignant Neoplasm" ) OR TITLE-ABS-KEY ( "Breast Malignant Neoplasms" ) OR TITLE-ABS-KEY ( "Malignant Tumor of Breast" ) OR TITLE-ABS-KEY ( "Breast Malignancy" ) OR TITLE-ABS-KEY ( "Breast Malign*" ) OR TITLE-ABS-KEY ( "Breast Malignant Tumor" ) OR TITLE-ABS-KEY ( "Breast Malignant Tumors" ) OR TITLE-ABS-KEY ( "Cancer of Breast" ) OR TITLE-ABS-KEY ( "Cancer of the Breast" ) OR TITLE-ABS-KEY ( "Mammary Carcinoma, Human" ) OR TITLE-ABS-KEY ( "Carcinoma, Human Mammary" ) OR TITLE-ABS-KEY ( "Carcinomas, Human Mammary" ) OR TITLE-ABS-KEY ( "Human Mammary Carcinomas" ) OR TITLE-ABS-KEY ( "Mammary Carcinomas, Human" ) OR TITLE-ABS-KEY ( "Human Mammary Carcinoma" ) OR TITLE-ABS-KEY ( "Mammary Neoplasms, Human" ) OR TITLE-ABS-KEY ( "Human Mammary Neoplasm" ) OR TITLE-ABS-KEY ( "Human Mammary Neoplasms" ) OR TITLE-ABS-KEY ( "Neoplasm, Human Mammary" ) OR TITLE-ABS-KEY ( "Neoplasms, Human Mammary" ) OR TITLE-ABS-KEY ( "Mammary Neoplasm, Human" ) OR TITLE-ABS-KEY ( "Breast Carcinoma" ) OR TITLE-ABS-KEY ( "Breast Carcinomas" ) OR TITLE-ABS-KEY ( "Carcinoma, Breast" ) OR TITLE-ABS-KEY ( "Carcinomas, Breast" ) ) AND ( TITLE-ABS-KEY ( "Pregnancy" ) OR TITLE-ABS-KEY ( "Pregnancies" ) OR TITLE-ABS-KEY ( "Gestation" ) OR TITLE-ABS-KEY ( "Pregnant" ) OR TITLE-ABS-KEY ( "Fertilization" ) OR TITLE-ABS-KEY ( "Fertilizations" ) OR TITLE-ABS-KEY ( "Fertilization, Delayed" ) OR TITLE-ABS-KEY ( "Delayed Fertilization" ) OR TITLE-ABS-KEY ( "Delayed Fertilizations" ) OR TITLE-ABS-KEY ( "Fertilizations, Delayed" ) OR TITLE-ABS-KEY ( "Fertilization, Polyspermic" ) OR TITLE-ABS-KEY ( "Fertilizations, Polyspermic" ) OR TITLE-ABS-KEY ( "Polyspermic Fertilization" ) OR TITLE-ABS-KEY ( "Polyspermic Fertilizations" ) OR TITLE-ABS-KEY ( "Conception" ) OR TITLE-ABS-KEY ( "Conceptions" ) OR TITLE-ABS-KEY ( "Conceiving" ) OR TITLE-ABS-KEY ( "Parturition" ) OR TITLE-ABS-KEY ( "Parturitions" ) OR TITLE-ABS-KEY ( "Birth" ) OR TITLE-ABS-KEY ( "Births" ) OR TITLE-ABS-KEY ( "Childbirth" ) OR TITLE-ABS-KEY ( "Childbirths" ) OR TITLE-ABS-KEY ( "Fertility" ) OR TITLE-ABS-KEY ( "Fecundability" ) OR TITLE-ABS-KEY ( "Fecundity" ) OR TITLE-ABS-KEY ( "Differential Fertility" ) OR TITLE-ABS-KEY ( "Fertility, Differential" ) OR TITLE-ABS-KEY ( "Fertility Determinants" ) OR TITLE-ABS-KEY ( "Determinant, Fertility" ) OR TITLE-ABS-KEY ( "Determinants, Fertility" ) OR TITLE-ABS-KEY ( "Fertility Determinant" ) OR TITLE-ABS-KEY ( "Subfecundity" ) OR TITLE-ABS-KEY ( "Fertility Preferences" ) OR TITLE-ABS-KEY ( "Fertility Preference" ) OR TITLE-ABS-KEY ( "Preference, Fertility" ) OR TITLE-ABS-KEY ( "Preferences, Fertility" ) OR TITLE-ABS-KEY ( "Fertility, Below Replacement" ) OR TITLE-ABS-KEY ( "Below Replacement Fertility" ) OR TITLE-ABS-KEY ( "Marital Fertility" ) OR TITLE-ABS-KEY ( "Fertility, Marital" ) OR TITLE-ABS-KEY ( "Natural Fertility" ) OR TITLE-ABS-KEY ( "Fertility, Natural" ) OR TITLE-ABS-KEY ( "World Fertility Survey" ) OR TITLE-ABS-KEY ( "Fertility Survey, World" ) OR TITLE-ABS-KEY ( "Fertility Surveys, World" ) OR TITLE-ABS-KEY ( "Survey, World Fertility" ) OR TITLE-ABS-KEY ( "Surveys, World Fertility" ) OR TITLE-ABS-KEY ( "World Fertility Surveys" ) OR TITLE-ABS-KEY ( "Fertility Incentives" ) OR TITLE-ABS-KEY ( "Fertility Incentive" ) OR TITLE-ABS-KEY ( "Obstetrics" ) OR TITLE-ABS-KEY ( "Obstetrical outcomes" ) ) AND ( TITLE-ABS-KEY ( "cohort studies" ) OR TITLE-ABS-KEY ( "case-control studies" ) OR TITLE-ABS-KEY ( "comparative study" ) OR TITLE-ABS-KEY ( "cohort*" ) OR TITLE-ABS-KEY ( "compared" ) OR TITLE-ABS-KEY ( "case control" ) OR TITLE-ABS-KEY ( "case series" ) OR TITLE-ABS-KEY ( "randomized controlled trial" ) OR TITLE-ABS-KEY ( "controlled clinical trial" ) OR TITLE-ABS-KEY ( "randomized" ) OR TITLE-ABS-KEY ( "placebo" ) OR TITLE-ABS-KEY ( "drug therapy" ) OR TITLE-ABS-KEY ( "randomly" ) OR TITLE-ABS-KEY ( "clinical trial" ) ) AND PUBYEAR > 1952 AND PUBYEAR < 2025

**7 CNKI**

(TKA % '乳腺恶性肿瘤' OR TKA % '乳房恶性肿瘤' OR TKA % '乳腺癌' OR TKA % '乳癌' OR TKA % '乳岩' OR TKA % '乳腺肿瘤' OR TKA % '乳房肿瘤') AND (TKA % '怀孕' OR TKA % '妊娠' OR TKA % '受精' OR TKA % '受孕' OR TKA % '受胎' OR TKA % '分娩' OR TKA % '生产' OR TKA % '生育' OR TKA % '生殖' OR TKA % '孕育') AND (AB % '队列研究' OR AB % '病例对照研究' OR AB % '临床试验' OR AB % '群组研究' OR AB % '定群研究' OR AB % '前瞻性研究' OR AB % '回顾性研究' OR AB % '随机对照' OR AB % '临床研究') time: Start year-2024-09-03

**8 Wang Fang**

(主题:(乳腺恶性肿瘤 or 乳房恶性肿瘤 or 乳腺癌 or 乳癌 or 乳岩 or 乳腺肿瘤 or 乳房肿瘤) and 主题:(怀孕 or 妊娠 or 受精 or 受孕 or 受胎 or 分娩 or 生产 or 生育 or 生殖 or 孕育) and 主题:(队列研究 or 病例对照研究 or 临床试验 or 群组研究 or 定群研究 or 前瞻性研究 or 回顾性研究 or 随机对照 or 临床研究)) time: Start year-2024-09-03

**9 VIP**

U=(乳腺恶性肿瘤+乳房恶性肿瘤+乳腺癌+乳癌+乳岩+乳腺肿瘤+乳房肿瘤) AND U=(怀孕+妊娠+受精+受孕+受胎+分娩+生产+生育+生殖+孕育) AND U=(队列研究+病例对照研究+临床试验+群组研究+定群研究+前瞻性研究+回顾性研究+随机对照+临床研究)

time: Start year-Present, search date:2024-09-03

**10 CBM**

1) "乳腺肿瘤"[不加权:扩展] 117370

2) ( "乳腺恶性肿瘤"[常用字段:智能] OR "乳房恶性肿瘤"[常用字段:智能] OR "乳腺癌"[常用字段:智能] OR "乳癌"[常用字段:智能] OR "乳岩"[常用字段:智能] OR "乳腺肿瘤"[常用字段:智能] OR "乳房肿瘤"[常用字段:智能])

3) (#2) OR (#1) 140391

4) "妊娠"[不加权:扩展] 125006

5) "受精"[不加权:扩展] 3504

6) "分娩"[不加权:扩展] 63497

7) "生育力"[不加权:扩展] 8309

8) ( "怀孕"[常用字段:智能] OR "妊娠"[常用字段:智能] OR "受精"[常用字段:智能] OR "受孕"[常用字段:智能] OR "受胎"[常用字段:智能] OR "分娩"[常用字段:智能] OR "生产"[常用字段:智能] OR "生育"[常用字段:智能] OR "生殖"[常用字段:智能] OR "孕育"[常用字段:智能]) 947544

9) (#8) OR (#7) OR (#6) OR (#5) OR (#4) 948762

10) "队列研究"[不加权:扩展] 244072

11) "病例对照研究"[不加权:扩展] 139460

12) "临床试验"[不加权:扩展] 208360

13) "前瞻性研究"[不加权:扩展] 11956

14) "回顾性研究"[不加权:扩展] 121578

15) "随机对照试验"[不加权:扩展] 198307

16) "临床研究"[不加权:扩展] 208910

17) ( "队列研究"[常用字段:智能] OR "病例对照研究"[常用字段:智能] OR "临床试验"[常用字段:智能] OR "群组研究"[常用字段:智能] OR "定群研究 OR前瞻性研究"[常用字段:智能] OR "回顾性研究"[常用字段:智能] OR "随机对照"[常用字段:智能] OR "临床研究"[常用字段:智能]) 807361

18) (#17) OR (#16) OR (#15) OR (#14) OR (#13) OR (#12) OR (#11) OR (#10) 807361

19) ((#18) AND (#9) AND (#3)) AND 1900-2024[日期] 390
