# Supplementary material for: Reproductive outcomes of pregnancy after breast cancer: an updated systematic review and meta-analysis
Source: Front Oncol. 2025 Sep 26;15:1569109. doi: 10.3389/fonc.2025.1569109 (PMC12510861; doi:10.3389/fonc.2025.1569109)
Supplement: Supplementary file 2 [file SupplementaryFile2.docx]

**eTable 1 Basic information of included articles**

| No. | First Author | Year | Country | Study design | Follow-up time | Tumor characteristics |
| --- | --- | --- | --- | --- | --- | --- |
| 1 | Priscilla Velentgas | 1999 | America | Prospective cohort study | mean 97 months (rage 22-179 months) | Ⅰ-Ⅱ, invasive |
| 2 | Kristina Dalberg | 2006 | Sweden | Retrospective cohort study | NA | NA |
| 3 | V Langagergaard | 2006 | Denmark | Retrospective cohort study | NA | excluded carcinoma in situ and sarcoma involving the breast |
| 4 | Louis Jacob | 2017 | German | Retrospective cohort study | at least 330 days | NA |
| 5 | Chelsea Anderson | 2018 | America | Prospective cohort study | median 6.0 years | Ⅰ-Ⅳ |
| 6 | Richard A. Anderson | 2022 | England | Retrospective cohort study | ≥ 6 years | NA |
| 7 | Kirsten Jorgensen | 2022 | America | Retrospective cohort study | NA | Ⅰ-Ⅲ |
| 8 | Moira Rushton | 2022 | Canada | Retrospective cohort study | median 12.8 years | Ⅰ-Ⅲ |
| 9 | Kristin Zeneé Black | 2017 | America | Retrospective cohort study | NA | NA |
| 10 | Mikael Hartman | 2013 | Singapore; | Retrospective cohort study | 0 to 44 years, a median follow-up of 4.5 years | NA |
| 11 | Helena M. Verkooijen | 2013 | Singapore | Retrospective cohort study | follow up to December 31, 2003 | NA |
| 12 | Deepika Garg | 2021 | America | Retrospective cohort study | NA | NA |
| 13 | Hak Min Lee | 2019 | South Korea | Prospective cohort study | 2-7 years | Ⅰ-Ⅲ |
| 14 | Leo Gkekos | 2024 | Sweden | Retrospective cohort study | NA | Ⅰ-Ⅲ |
| 15 | [Ceren Sunguc](https://www.thelancet.com/journals/lanonc/article/PIIS1470-2045(24)00269-9/fulltext) | 2024 | England | Retrospective cohort study | study end date (March 31, 2022), or until survivors were age 50 years | NA |
| 16 | Chelsea Anderson | 2024 | America | Retrospective cohort study | NA | NA |
| 17 | Nathalie Auger | 2024 | Canada | Retrospective cohort study | NA | NA |
| 18 | Richard A. Anderson | 2018 | England | Retrospective cohort study | NA | NA |
| 19 | Nigel Pereira | 2016 | America | Retrospective cohort study | NA | NA |
| 20 | Barbara Luke | 2016 | America | Retrospective cohort study | NA | NA |
| 21 | Hanne Stensheim | 2011 | Norway | Retrospective cohort study | median 3.6 years | NA |
| 22 | Laura-Maria S. Madanat | 2008 | Finland | Retrospective cohort study | NA | NA |
| 23 | Nancy N Baxter | 2013 | Canada | Retrospective cohort study | median 12 years | at least 5 years recurrence-free |
| 24 | Kathleen P. Hartnett | 2017 | America | Retrospective cohort study | NA | invasive and ductal carcinoma in situ |
| 25 | Hanne Stensheim | 2013 | Norway | Retrospective cohort study | NA | NA |
| 26 | Kimberly K. Ma | 2020 | America | Case control study | NA | NA |

Abbreviations: NA, not applicable

**eTable 2 Reproductive outcomes of included articles**

| No. | First Author | pregnancy outcomes | obstetrical outcomes | fetal outcomes | pregnancy complications |
| --- | --- | --- | --- | --- | --- |
| 1 | Priscilla Velentgas | Miscarriage | NA | NA | NA |
| 2 | Kristina Dalberg | NA | Pregnancy bleeding, Instrumental delivery, Cesarean section, Delivery complication | Very preterm birth, PTB, Post-term delivery, LBW, Birth weight ＞4500 g, Stillbirth, Live born, Apgar score, Malformation, Birth trauma | NA |
| 3 | V Langagergaard | NA | PTB | LBW at term, Stillbirth, Abnormalities, Male proportion of newborns | NA |
| 4 | Louis Jacob | Early and late pregnancy loss, Spontaneous abortion, Medical abortion, Unspecified abortion, Delivery of a live-born child, Stillbirth | PTB, Spontaneous delivery, Cesarean delivery, Preterm contractions without preterm birth, Hemorrhage in early pregnancy without fetal loss | Suspected poor fetal growth, Fetal malpresentation | Genito-urinary infections, Known or suspected abnormality of pelvic organs, Gestational diabetes mellitus, Pre-eclampsia incl. HELLP syndrome, Disorders of breast and lactation associated with childbirth |
| 5 | Chelsea Anderson | NA | Live birth rate, Cumulative incidence of live birth: case series of BC patients 5-year cumulative incidence of live birth, 10-year cumulative incidence of live birth, Cumulative incidence of cesarean delivery, PTB | LBW, Small for gestational age (SGA) | NA |
| 6 | Richard A. Anderson | NA | Live birth | NA | NA |
| 7 | Kirsten Jorgensen | NA | PTB, Very preterm birth, Cesarean Delivery | SGA, Neonatal Morbidity, Fetal demise | severe maternal morbidity based on the Centers for Disease Control and Prevention algorithm |
| 8 | Moira Rushton | NA | Childbirth | NA | NA |
| 9 | Kristin Zeneé Black | NA | PTB | LBW, SGA | NA |
| 10 | Mikael Hartman | NA | Standardized Birth Ratio | NA | NA |
| 11 | Helena M. Verkooijen | NA | Live birth rate | NA | NA |
| 12 | Deepika Garg | NA | Live birth rate | NA | NA |
| 13 | Hak Min Lee | Miscarriage | Childbirths, Full-term delivery, Preterm delivery, Preterm labor, Plural birth, Hydramnios/Oligo, Obstetric hemorrhage, Premature rupture of membranes | NA | Pre-eclampsia |
| 14 | Leo Gkekos | NA | Very preterm birth, PTB, Induced delivery, Planned PTB, Spontaneous PTB, Placental abruption, Prelabor rupture of membranes, Caesarean section, Assisted vaginal, Planned cesarean, Emergency cesarean, Birth injury | Stillbirth, LBW, SGA, Apgar score at 5 minutes, Congenital malformations, Neonatal mortality within 27 days | Gestational diabetes, Gestational hypertension, Pre-eclampsia, Bleeding during pregnancy |
| 15 | [Ceren Sunguc](https://www.thelancet.com/journals/lanonc/article/PIIS1470-2045(24)00269-9/fulltext) | Birth rate | Antepartum hemorrhage, Prolonged pregnancy, Preterm labor and delivery, Unsuccessful induction of labor, Abnormalities of forces of labor, Long labor, Obstructed labor due to malposition and malpresentation of fetus, Intrapartum hemorrhage, Perineal laceration, Postpartum hemorrhage, Retained placenta and membranes without hemorrhage, Puerperal infections | Malpresentation of fetus, Polyhydramnios, Other disorders of amniotic fluid and membranes, Premature rupture of membranes, Placental disorders, Placenta praevia, Fetal stress, Umbilical cord complications | Pre-existing hypertension complicating pregnancy, Gestational oedema and proteinuria without hypertension, Gestational hypertension, Pre-eclampsia, Unspecified maternal hypertension, Infections of genitourinary tract in pregnancy, Diabetes arising in pregnancy |
| 16 | Chelsea Anderson | NA | PTB | LBW, SGA, At least one birth defect | NA |
| 17 | Nathalie Auger | NA | NA | Birth defects | NA |
| 18 | Richard A. Anderson | NA | NA | NA | NA |
| 19 | Nigel Pereira | NA | NA | Live birth rate | NA |
| 20 | Barbara Luke | Pregnant rate | Live birth | NA | NA |
| 21 | Hanne Stensheim | Pregnancy rate | NA | NA | NA |
| 22 | Laura-Maria S. Madanat | NA | Live birth rate | NA | NA |
| 23 | Nancy N Baxter | NA | Cumulative 10-year rate of childbirth | NA | NA |
| 24 | Kathleen P. Hartnett | NA | PTB, Very preterm birth, LBW, Very LBW, LBW at term, SGA, Undergoing elective or emergency cesarian section, Admission to NICU, Apgar score < 7 at 5 minutes | NA | NA |
| 25 | Hanne Stensheim | NA | NA | Perinatal mortality, PTB, LBW, LBW at term, Primiparous congenital anomalies | NA |
| 26 | Kimberly K. Ma | Accumulated miscarriage, Accumulated induced abortion, Accumulated ectopic pregnancy, Non-live birth, Cesarean section | Accumulated live single birth, Accumulated multiple gestation, Accumulated stillbirth | NA | NA |

Abbreviations: NA, not applicable; OR, odds ratio; RR, relative risk; HR, hazard ratio; CI, confidence intervals; PTB, preterm birth; LBW, low birth weight; SGA, small for gestational age

**eTable 3 Quality evaluation**

| Title | First-Author | Year | Study design | selection | comparability | exposure | Total score |
| --- | --- | --- | --- | --- | --- | --- | --- |
| Pregnancy after Breast Carcinoma Outcomes and Influence on Mortality | Priscilla Velentgas | 1999 | Prospective cohort study | 3 | 1 | 2 | 6 |
| Birth outcome in women with previously treated breast cancer—A population-based cohort study from Sweden | Kristina Dalberg | 2006 | Retrospective cohort study | 3 | 2 | 3 | 8 |
| Birth outcome in women with breast cancer | V Langagergaard | 2006 | Retrospective cohort study | 3 | 2 | 2 | 7 |
| Impact of prior breast cancer on mode of delivery and pregnancy associated disorders: a retrospective analysis of subsequent pregnancy outcomes | Louis Jacob | 2017 | Retrospective cohort study | 3 | 1 | 2 | 6 |
| Live birth outcomes after adolescent and young adult breast cancer | Chelsea Anderson | 2018 | Prospective cohort study | 3 | 2 | 2 | 7 |
| Family size and duration of fertility in female cancer survivors: a population-based analysis | Richard A. Anderson | 2022 | Retrospective cohort study | 3 | 1 | 2 | 6 |
| Obstetric and Neonatal Outcomes One or More Years After a Diagnosis of Breast Cancer | Kirsten JORGENSEN | 2022 | Retrospective cohort study | 3 | 2 | 2 | 7 |
| Reproductive Outcomes in Young Breast Cancer Survivors Treated (15-39) in Ontario, Canada | Moira Rushton | 2022 | Retrospective cohort study | 3 | 1 | 2 | 6 |
| Prevalence of preterm, low birthweight,and small for gestational age delivery after breast cancer diagnosis: a population based study | Kristin Zeneé Black | 2017 | Retrospective cohort study | 3 | 2 | 2 | 7 |
| Birth Rates Among Female Cancer Survivors | Mikael Hartman | 2013 | Retrospective cohort study | 3 | 1 | 3 | 7 |
| Mortality among offspring of women diagnosed with cancer: A population-based cohort study | Helena M. Verkooijen | 2013 | Retrospective cohort study | 2 | 1 | 2 | 5 |
| Cancer treatment is associated with a measurable decrease in live births in a large, population-based study | Deepika Garg | 2021 | Retrospective cohort study | 3 | 1 | 2 | 6 |
| Childbirth in young Korean women with previously treated breast cancer: The SMARTSHIP study | Hak Min Lee | 2019 | Prospective cohort study | 4 | 2 | 2 | 8 |
| Obstetric and perinatal outcomes in women with  previous breast cancer: a nationwide study of  singleton births 1973-2017 | Leo Gkekos | 2024 | Retrospective cohort study | 3 | 1 | 2 | 6 |
| Risks of adverse obstetric outcomes among female survivors of adolescent and young adult cancer in England (TYACSS): a population-based, retrospective cohort study | [Ceren Sunguc](https://www.thelancet.com/journals/lanonc/article/PIIS1470-2045(24)00269-9/fulltext) | 2024 | Retrospective cohort study | 2 | 2 | 2 | 6 |
| Risk of adverse birth outcomes after adolescent and young adult cancer | Chelsea Anderson | 2024 | Retrospective cohort study | 3 | 2 | 2 | 7 |
| Association of maternal cancer with congenital anomalies in offspring | Nathalie Auger | 2024 | Retrospective cohort study | 3 | 1 | 2 | 6 |
| The impact of cancer on subsequent chance of pregnancy: a populationbased analysis | Richard A. Anderson | 2018 | Retrospective cohort study | 2 | 1 | 2 | 5 |
| Comparison of ovarian stimulation response in patients with breast cancer undergoing ovarian stimulation with letrozole and gonadotropins to patients undergoing ovarian stimulation with gonadotropins alone for elective cryopreservation of oocytes | Nigel Pereira | 2016 | Retrospective cohort study | 3 | 1 | 1 | 5 |
| Assisted reproductive technology use and outcomes among women with a history of cancer | Barbara Luke | 2016 | Retrospective cohort study | 3 | 2 | 2 | 7 |
| Pregnancy after adolescent and adult cancer: a population-based matched cohort study | Hanne Stensheim | 2011 | Retrospective cohort study | 3 | 1 | 2 | 6 |
| Probability of parenthood after early onset cancer: A population-based study | Laura-Maria S. Madanat | 2008 | Retrospective cohort study | 3 | 1 | 2 | 6 |
| A population-based study of rates of childbirth in recurrence-free female young adult survivors of Non-gynecologic malignancies | Nancy N Baxter | 2013 | Retrospective cohort study | 3 | 1 | 2 | 6 |
| The risk of preterm birth and growth restriction in pregnancy after cancer | Kathleen P. Hartnett | 2017 | Retrospective cohort study | 3 | 1 | 3 | 7 |
| Birth outcomes among offspring of adult cancer survivors: A population-based study | Hanne Stensheim | 2013 | Retrospective cohort study | 3 | 1 | 3 | 7 |
| Obstetric Outcomes in Young Women with Breast Cancer: Prior, Postpartum, and Subsequent Pregnancies | Kimberly K. Ma | 2020 | Case control study | 3 | 1 | 1 | 5 |
