# Supplementary material for: Reproductive outcomes of pregnancy after breast cancer: an updated systematic review and meta-analysis
Source: Front Oncol. 2025 Sep 26;15:1569109. doi: 10.3389/fonc.2025.1569109 (PMC12510861; doi:10.3389/fonc.2025.1569109)

**eFigure 1 Pregnancy outcomes comparing between breast cancer patients and healthy women from the general population.**

A) Prevalence of pregnancy


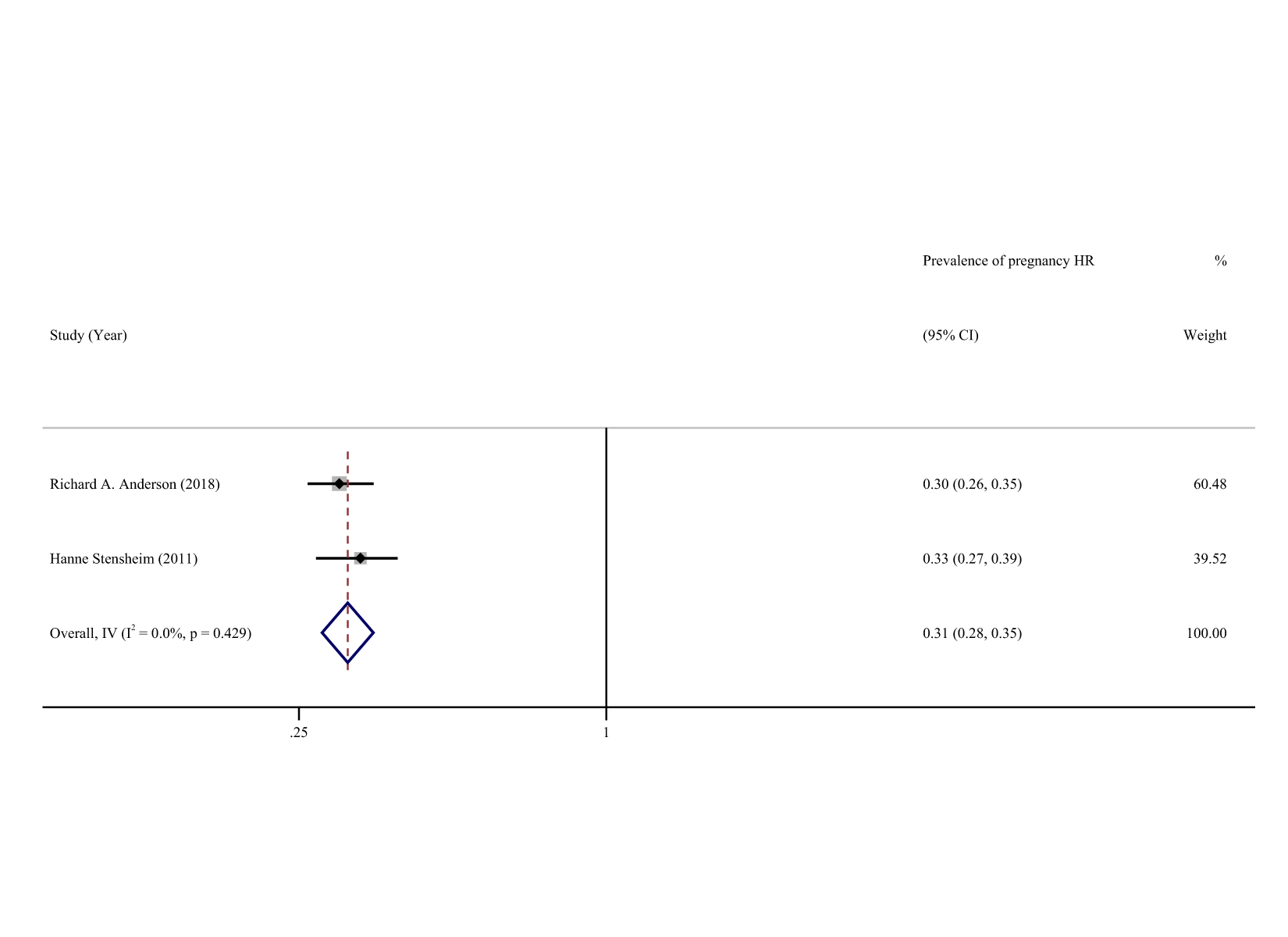


Random effect: p=0.000.

Egger’s test: not calculable


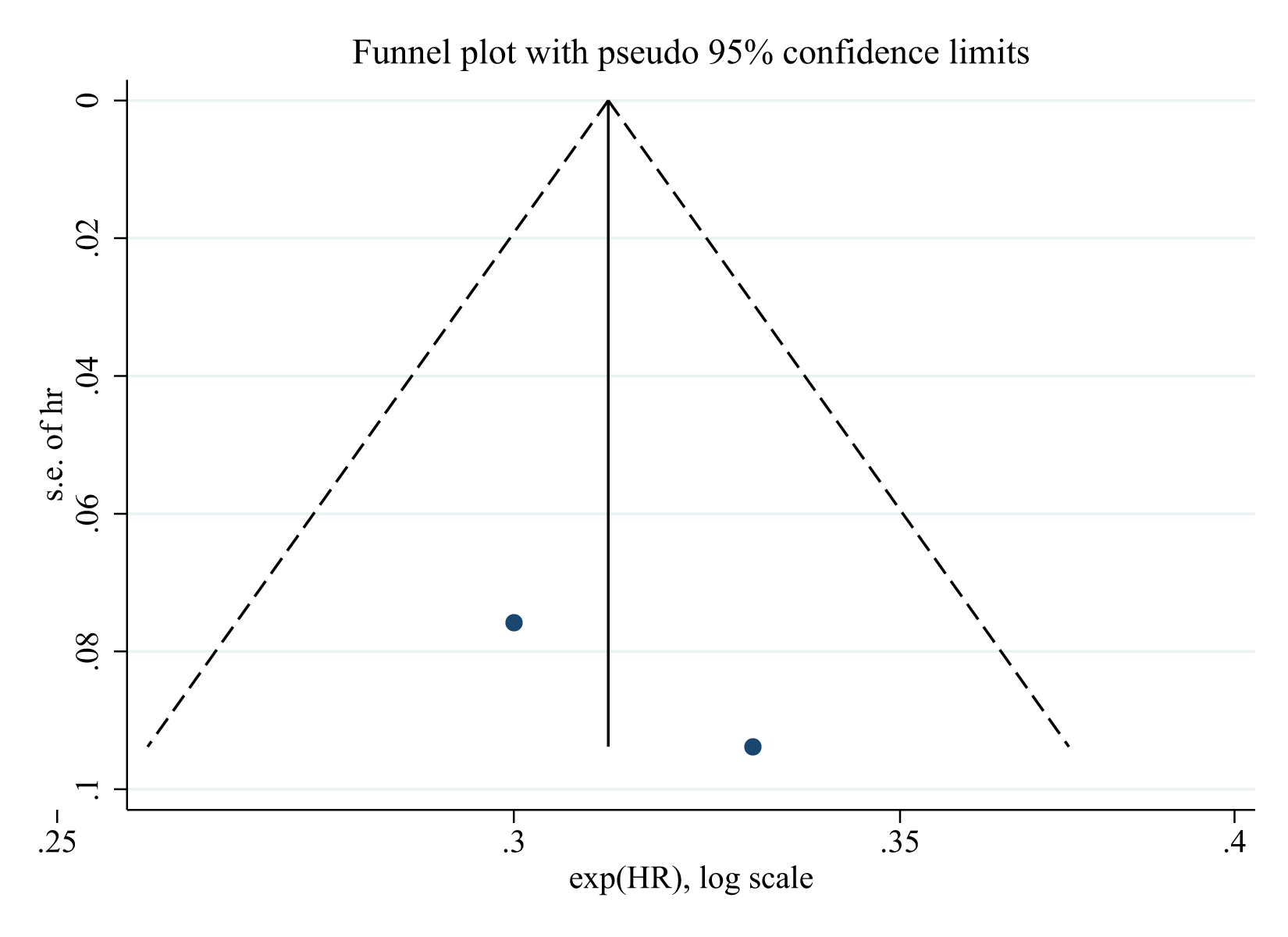

1. Completed pregnancy

① OR


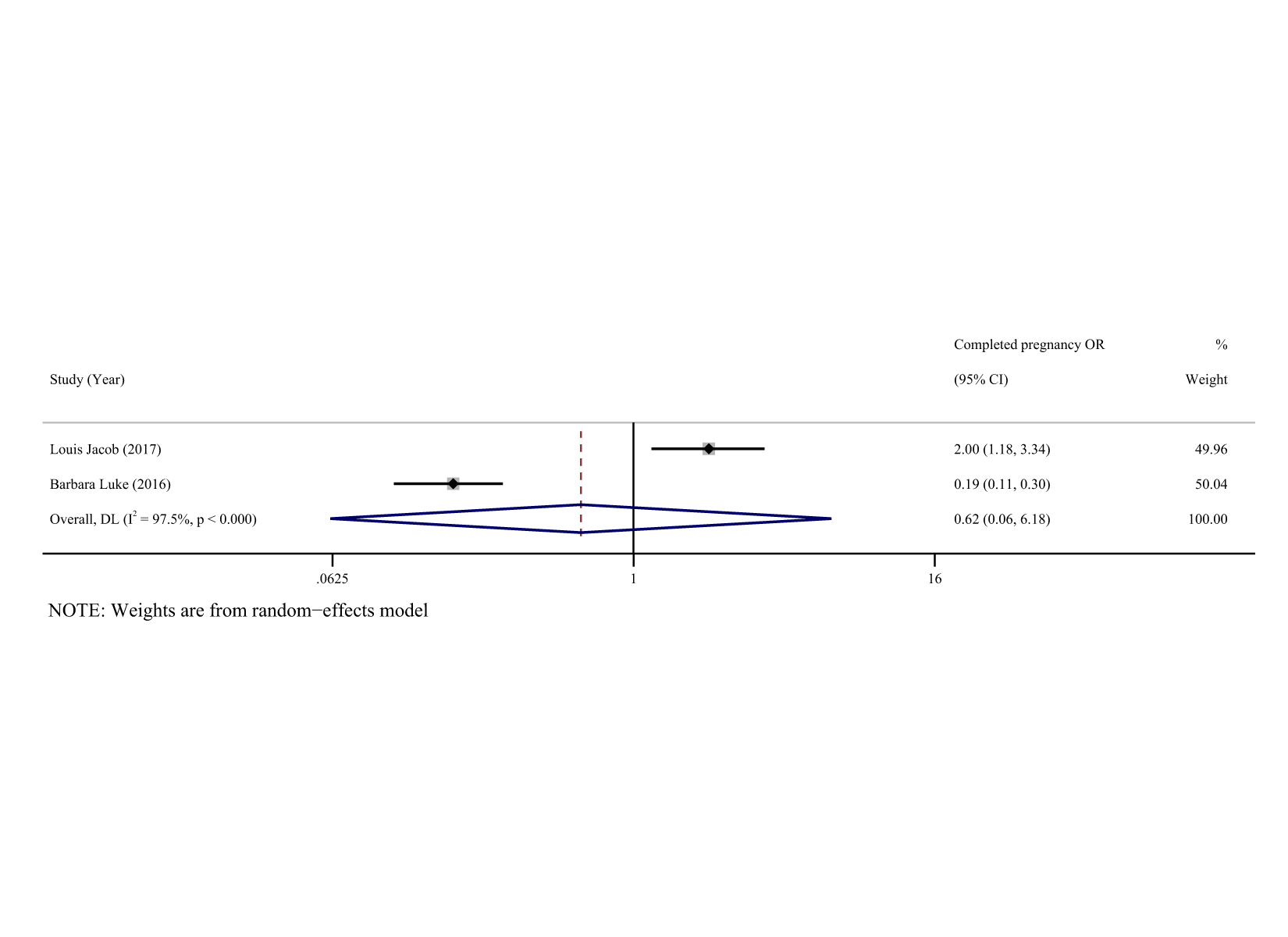


Random effect: p=0.680.

Egger’s test: not calculable


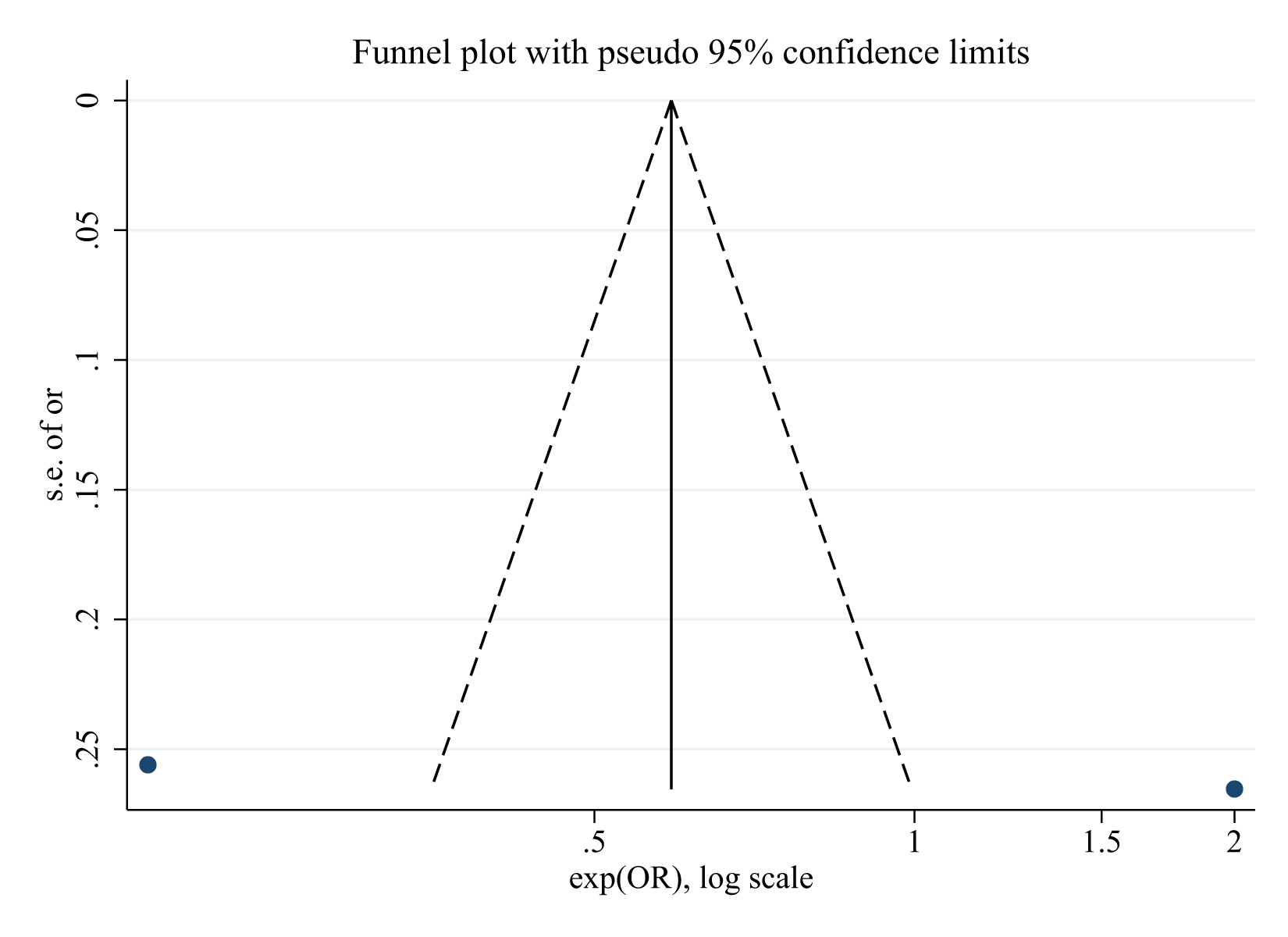

② RR


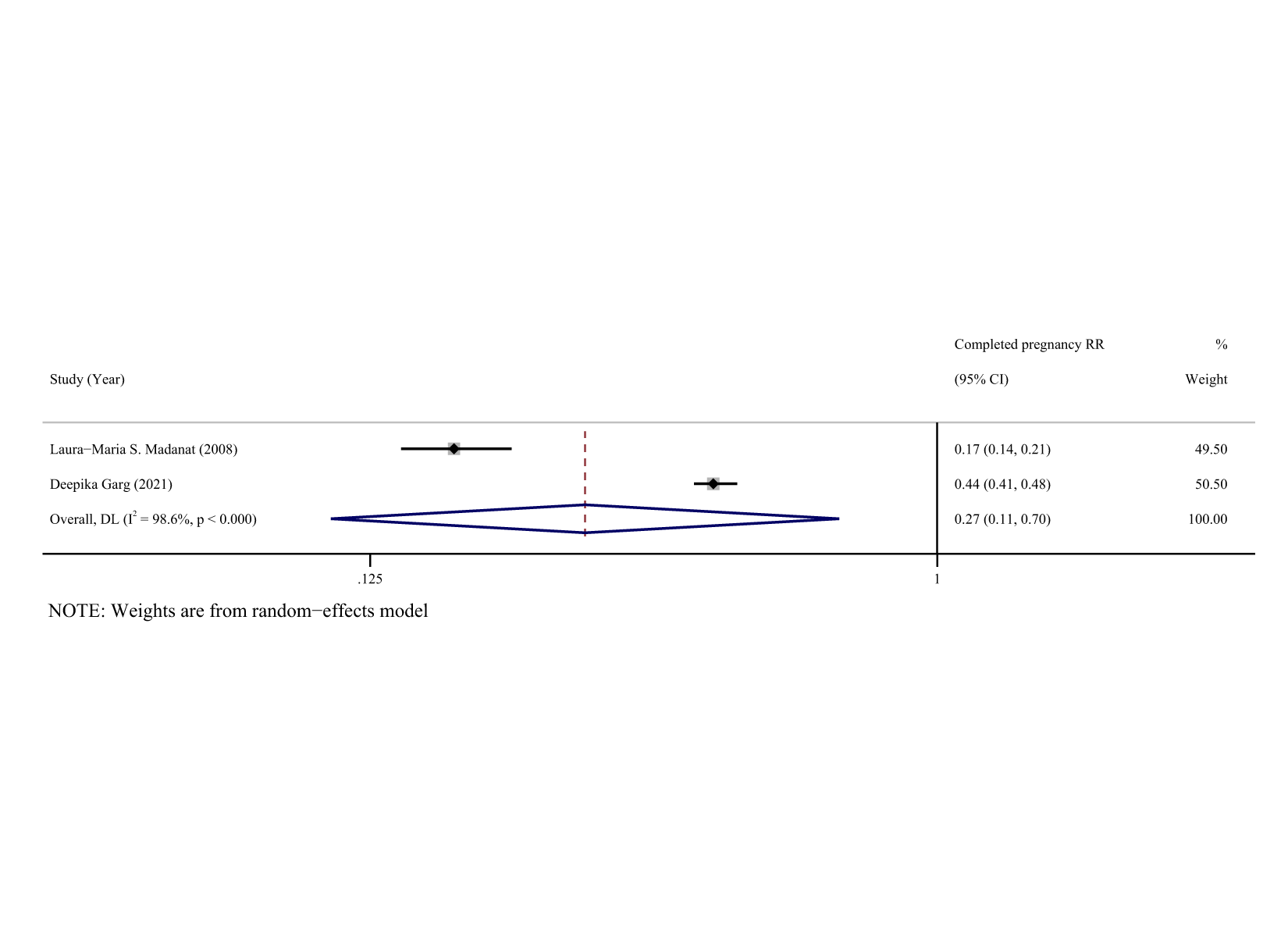


Random effect: p=0.007.

Egger’s test: not calculable


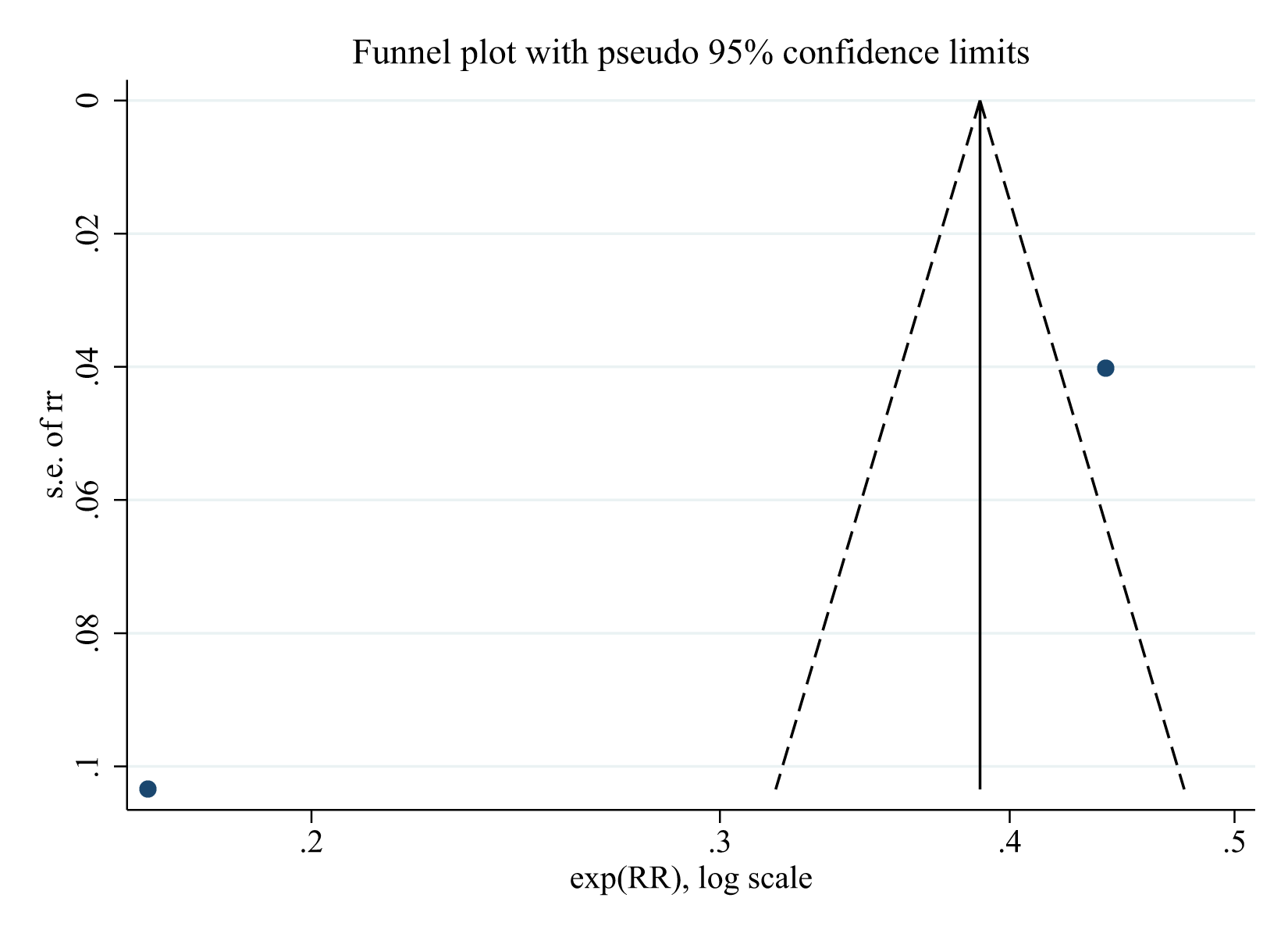

③ SBR


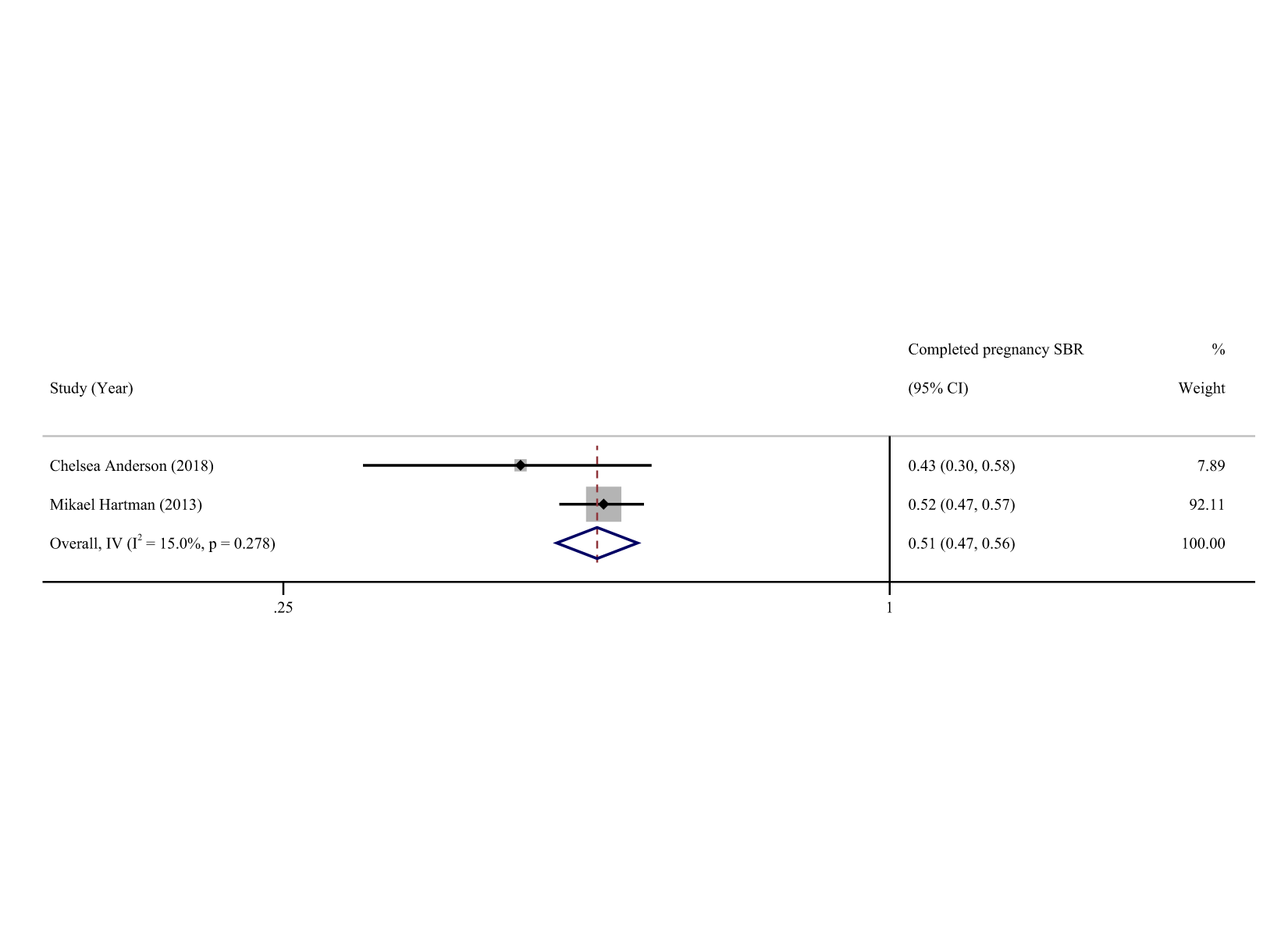


Random effect: p=0.000.

Egger’s test: not calculable


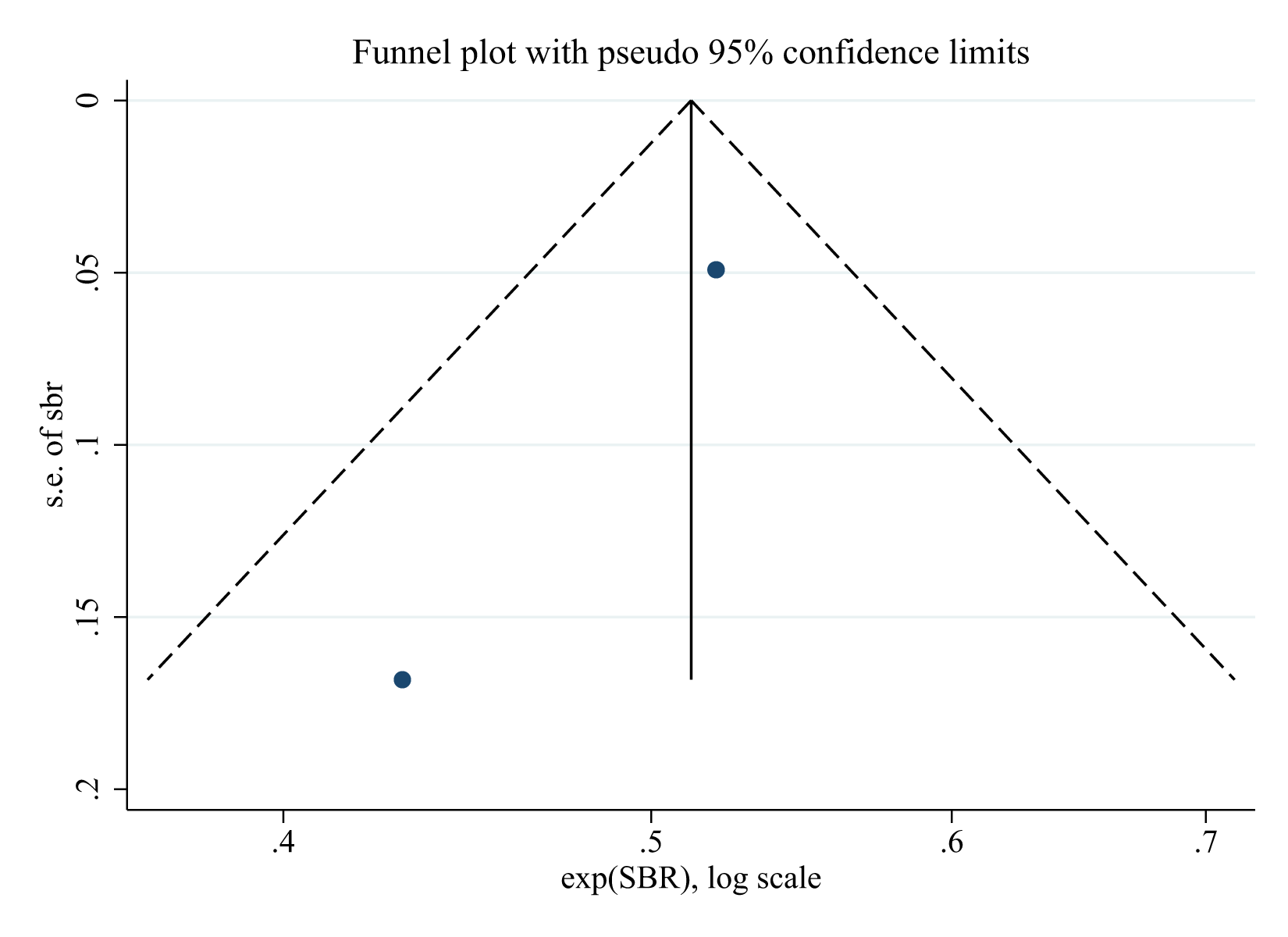

④ HR


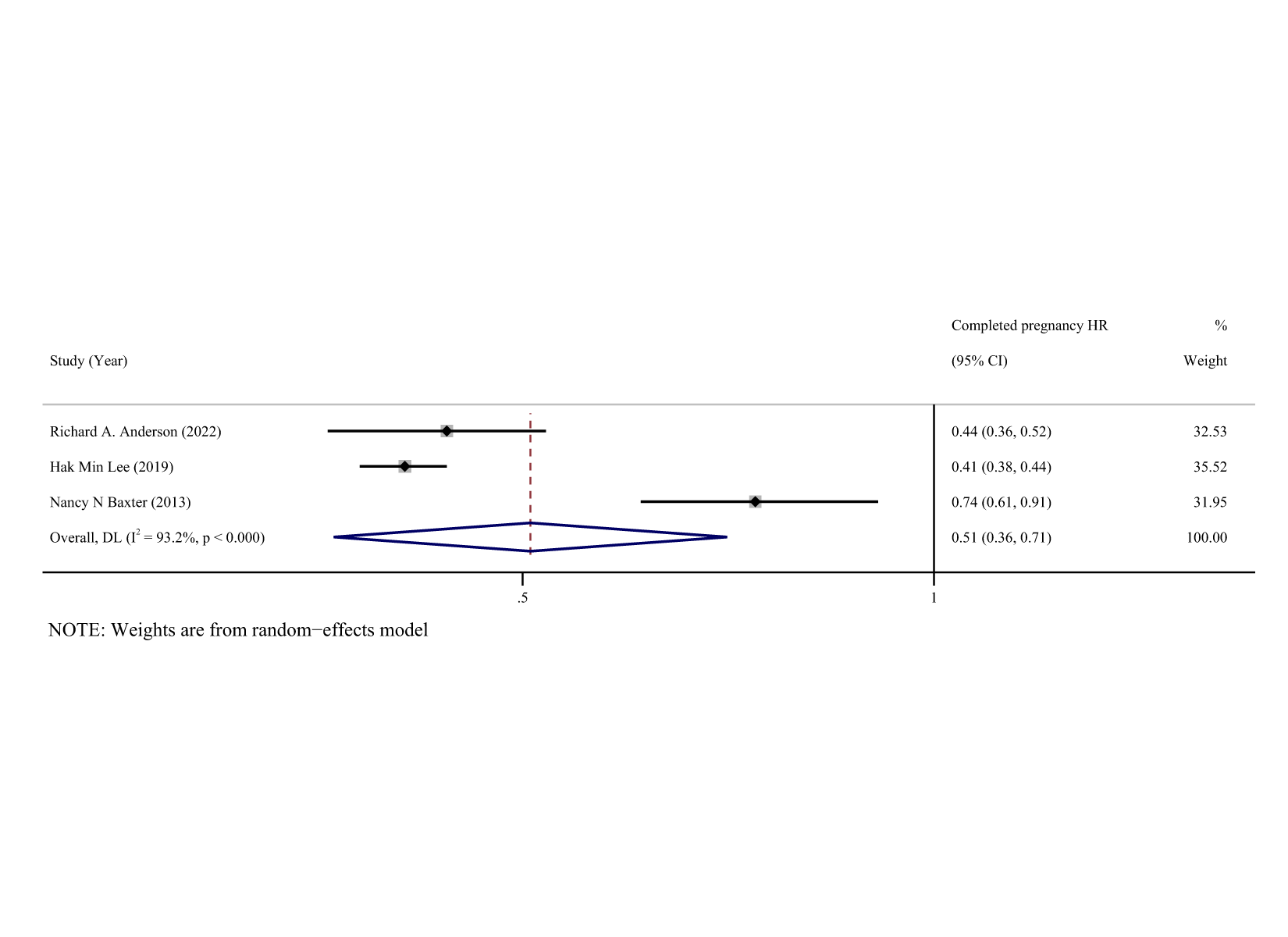


Random effect: p=0.000.

Egger’s test: p=0.436.


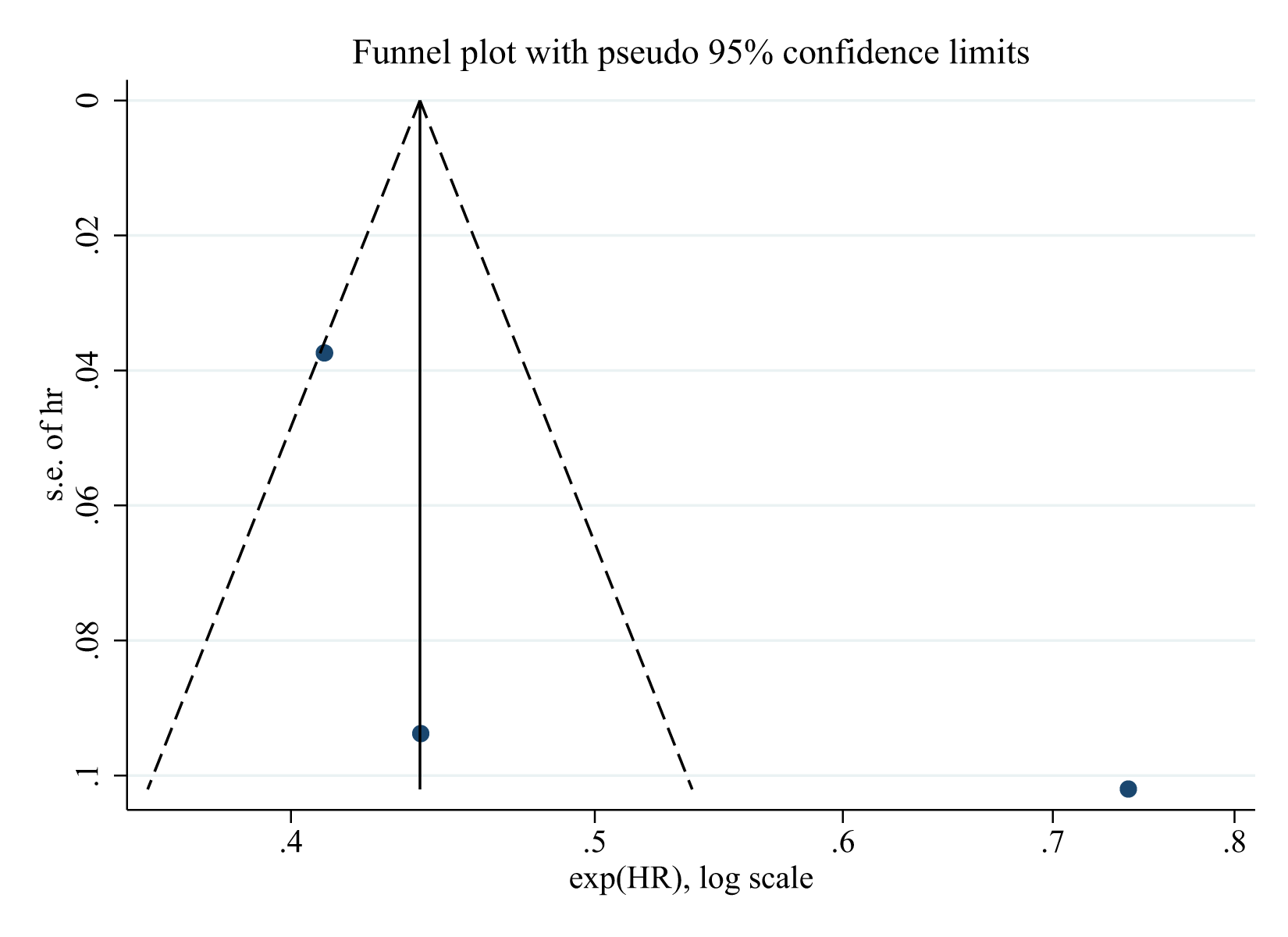

⑤ Binary variable


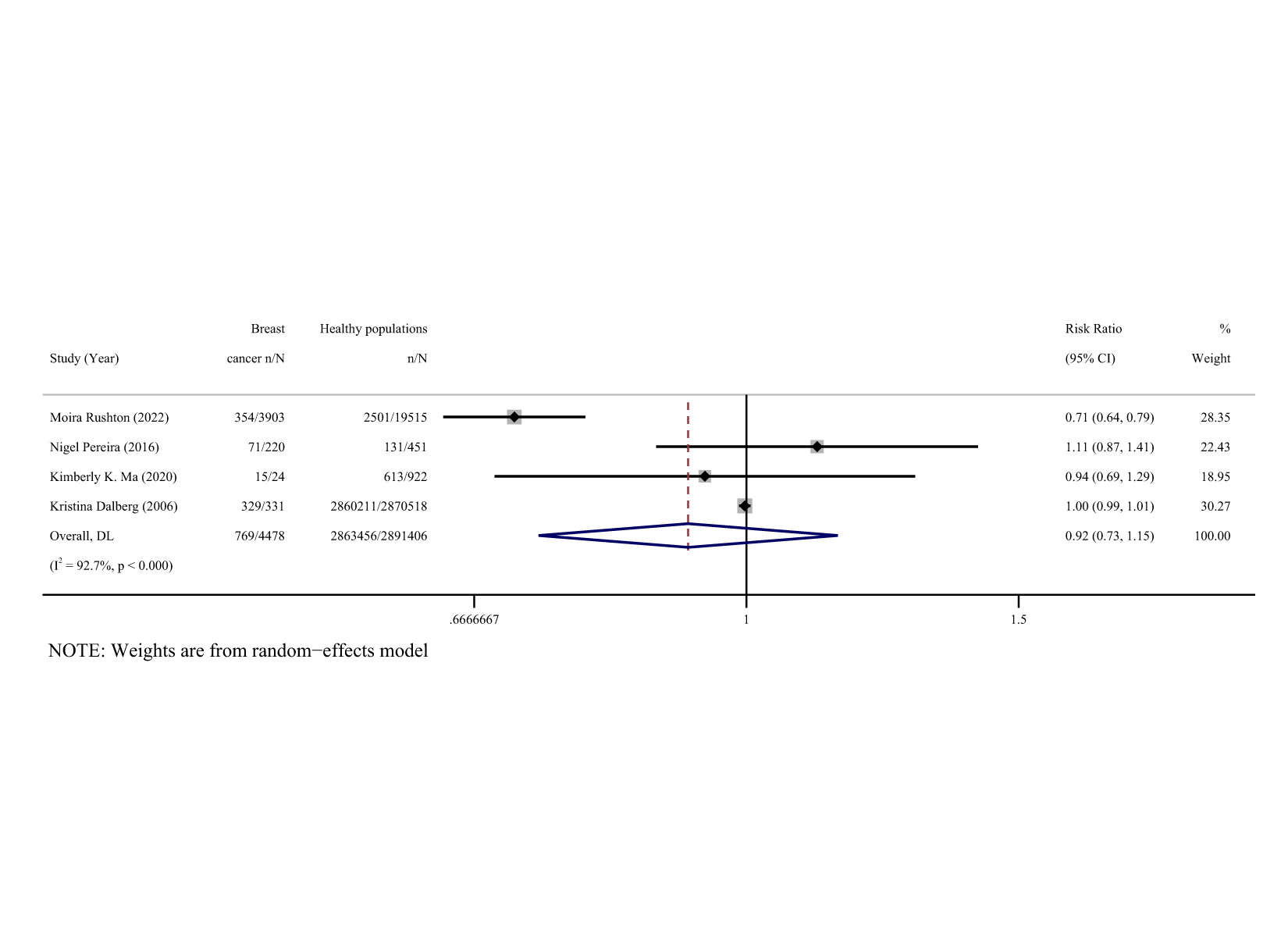


Random effect: p=0.445.

Egger’s test: p=0.498


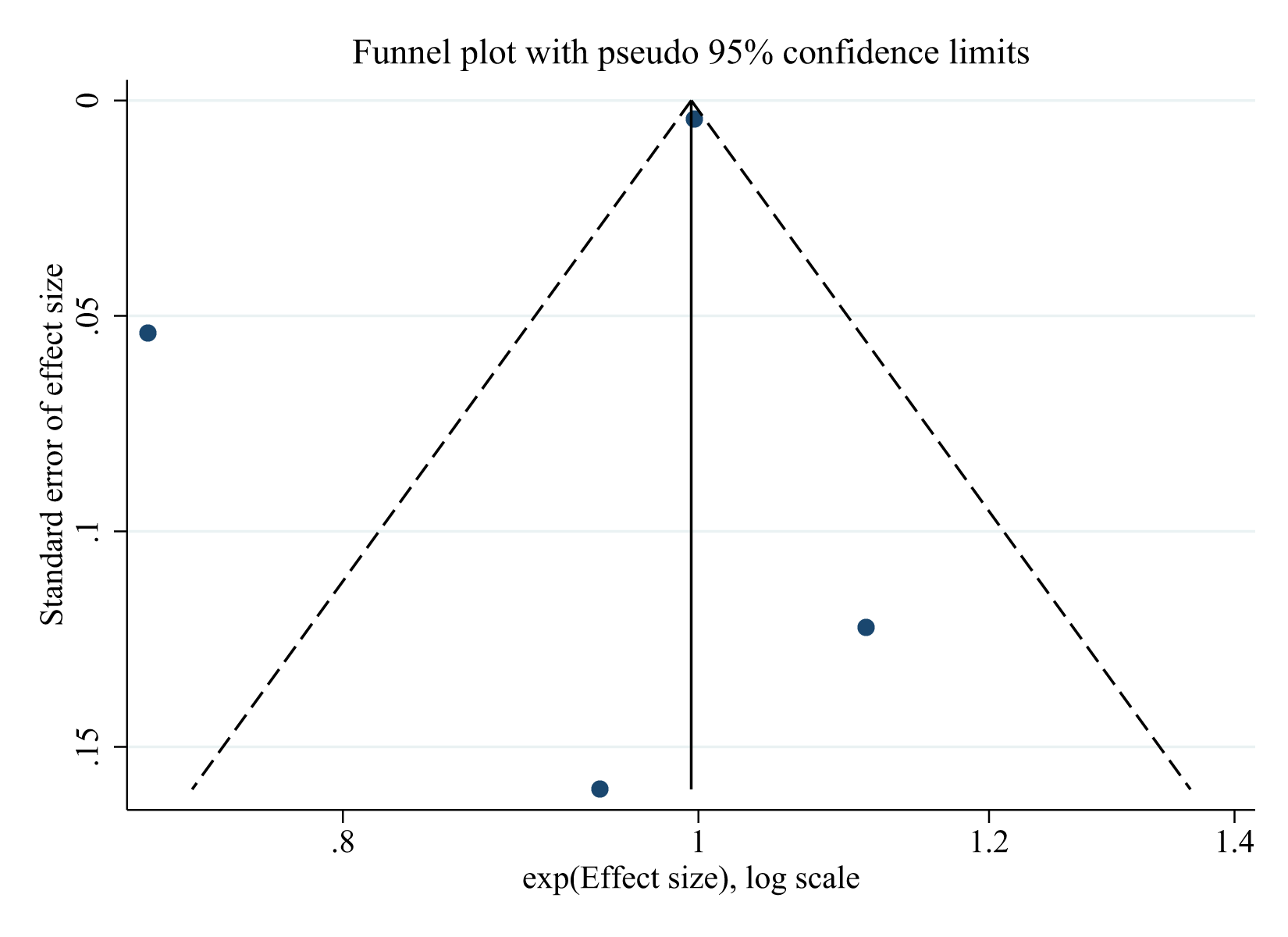

1. Spontaneous abortion


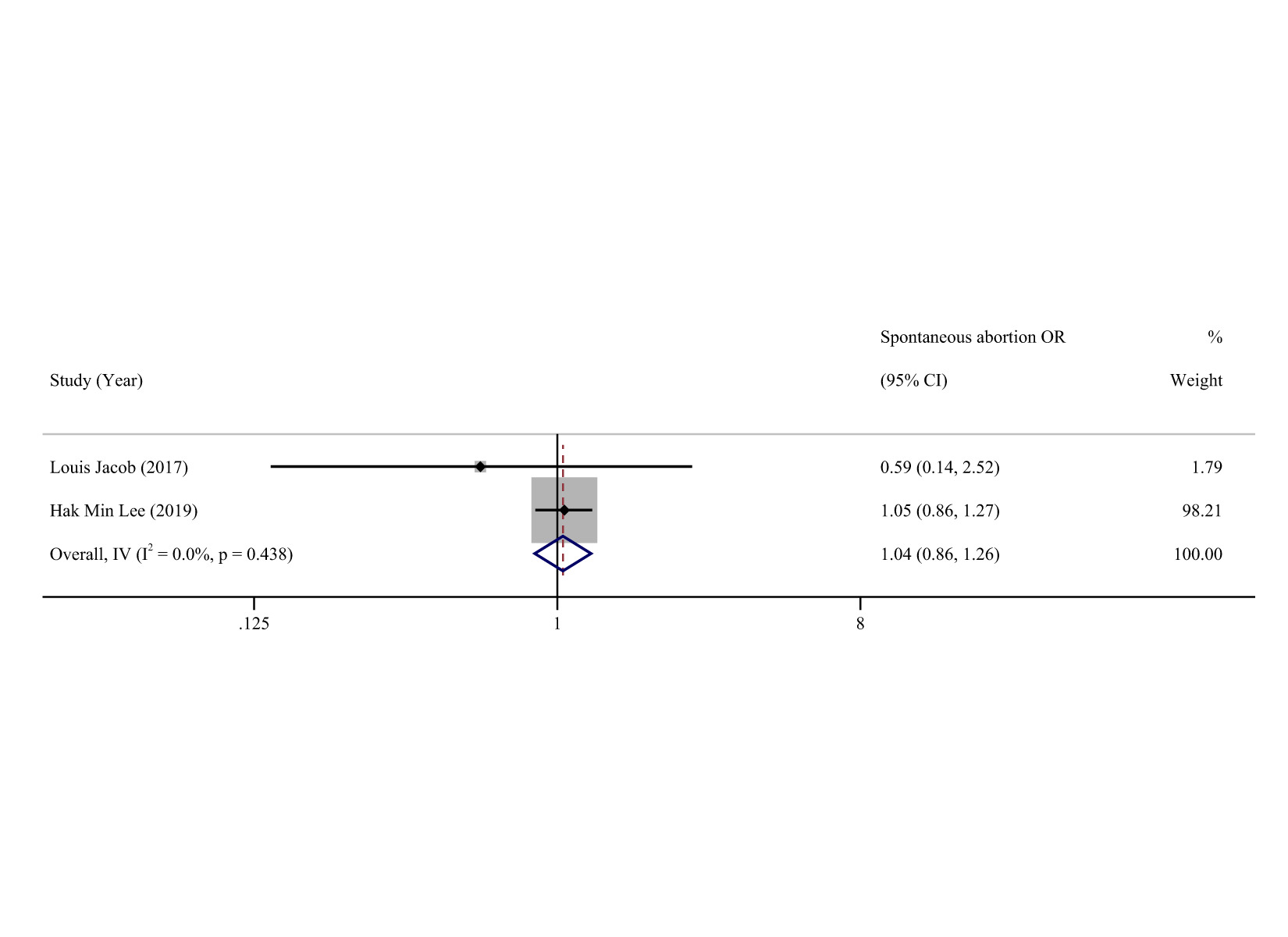


Random effect: p=0.696.

Egger’s test: not calculable


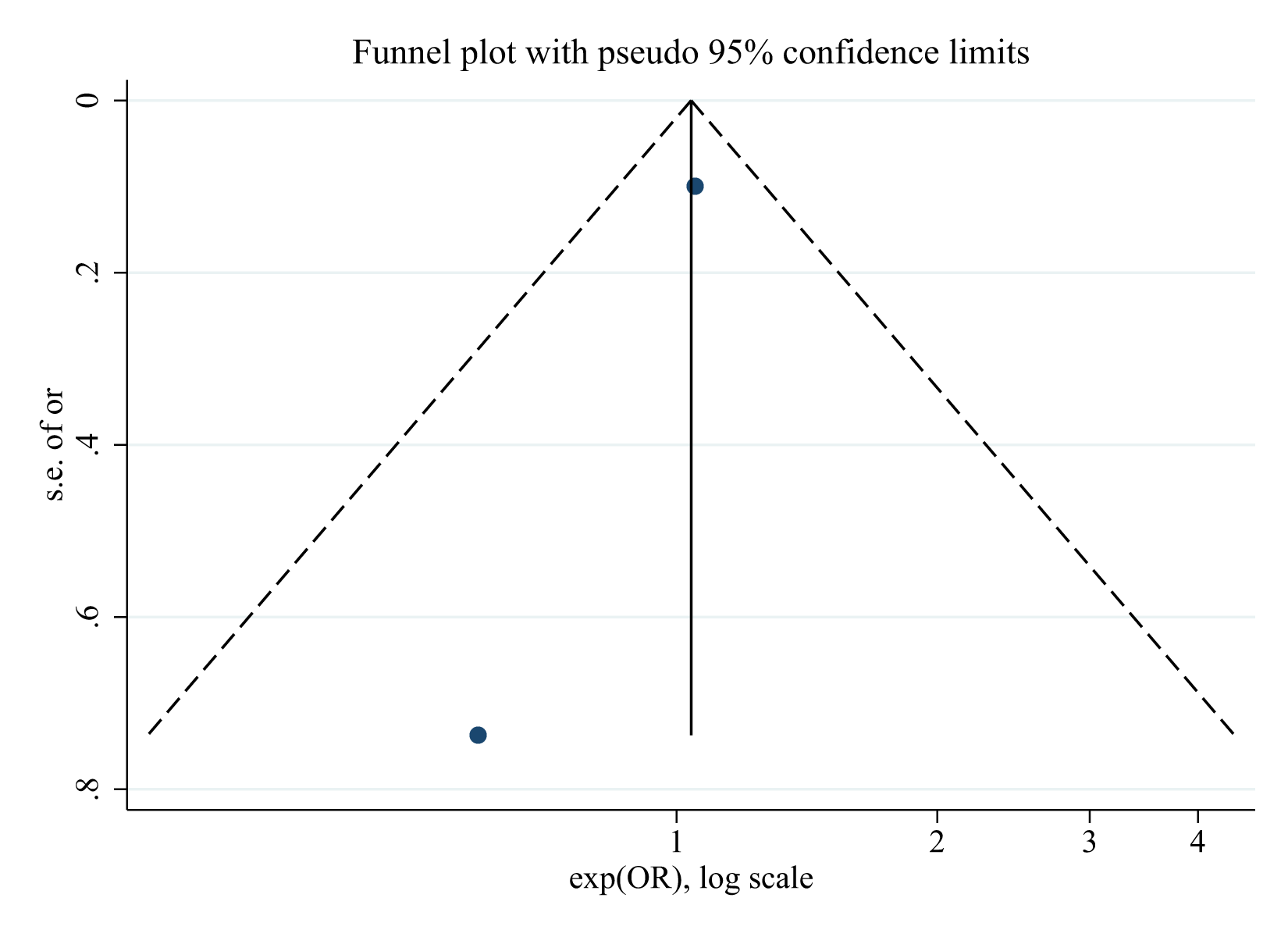

*Abbreviations: OR, odds ratio; RR, relative risk; HR, hazard ratio; CI, confidence intervals

**eFigure 2 Obstetrical outcomes comparing between breast cancer patients and healthy women from the general population.**

A) Children birth

① Binary variable


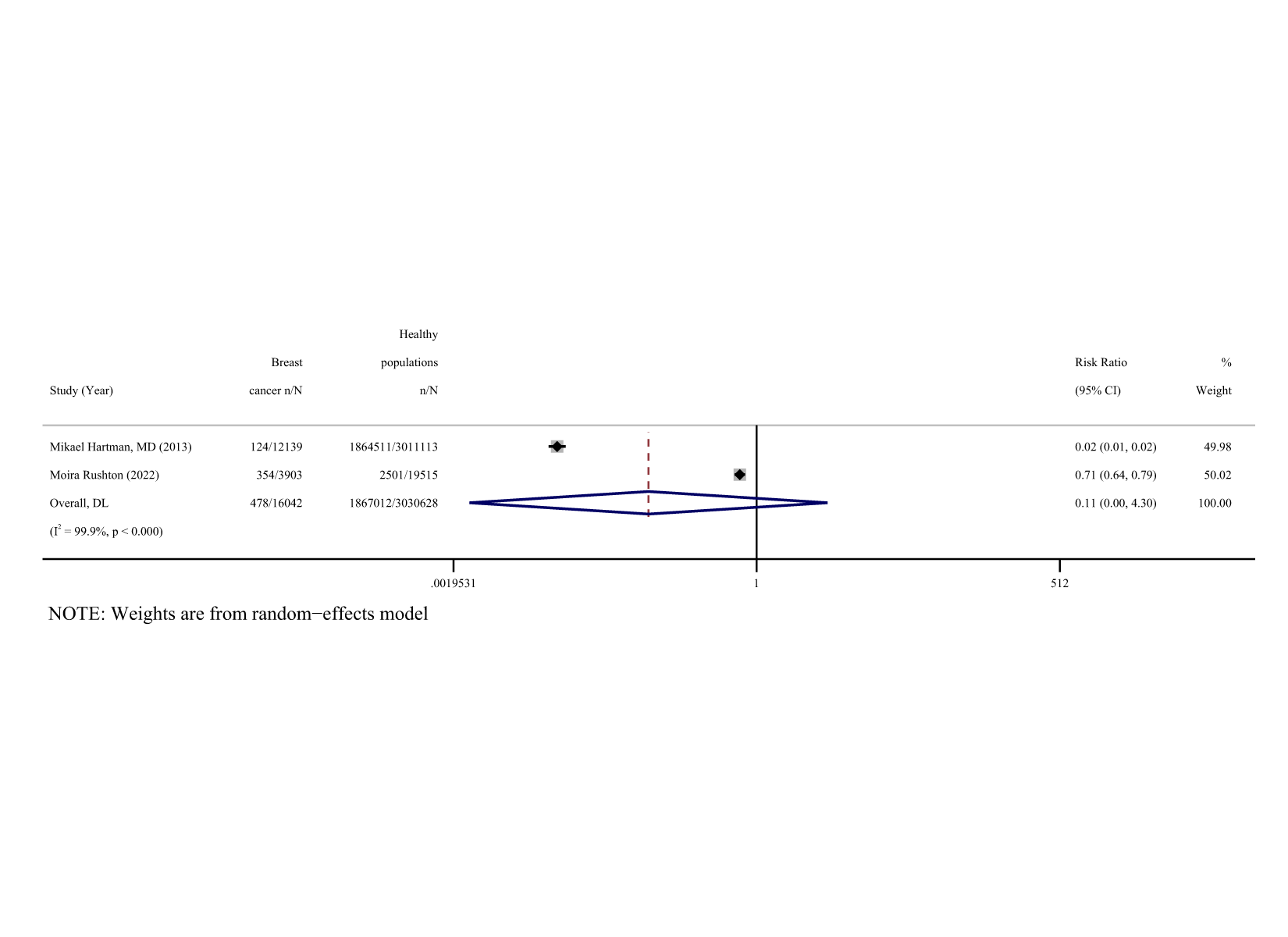


Random effect: p=0.237.

Egger’s test: not calculable


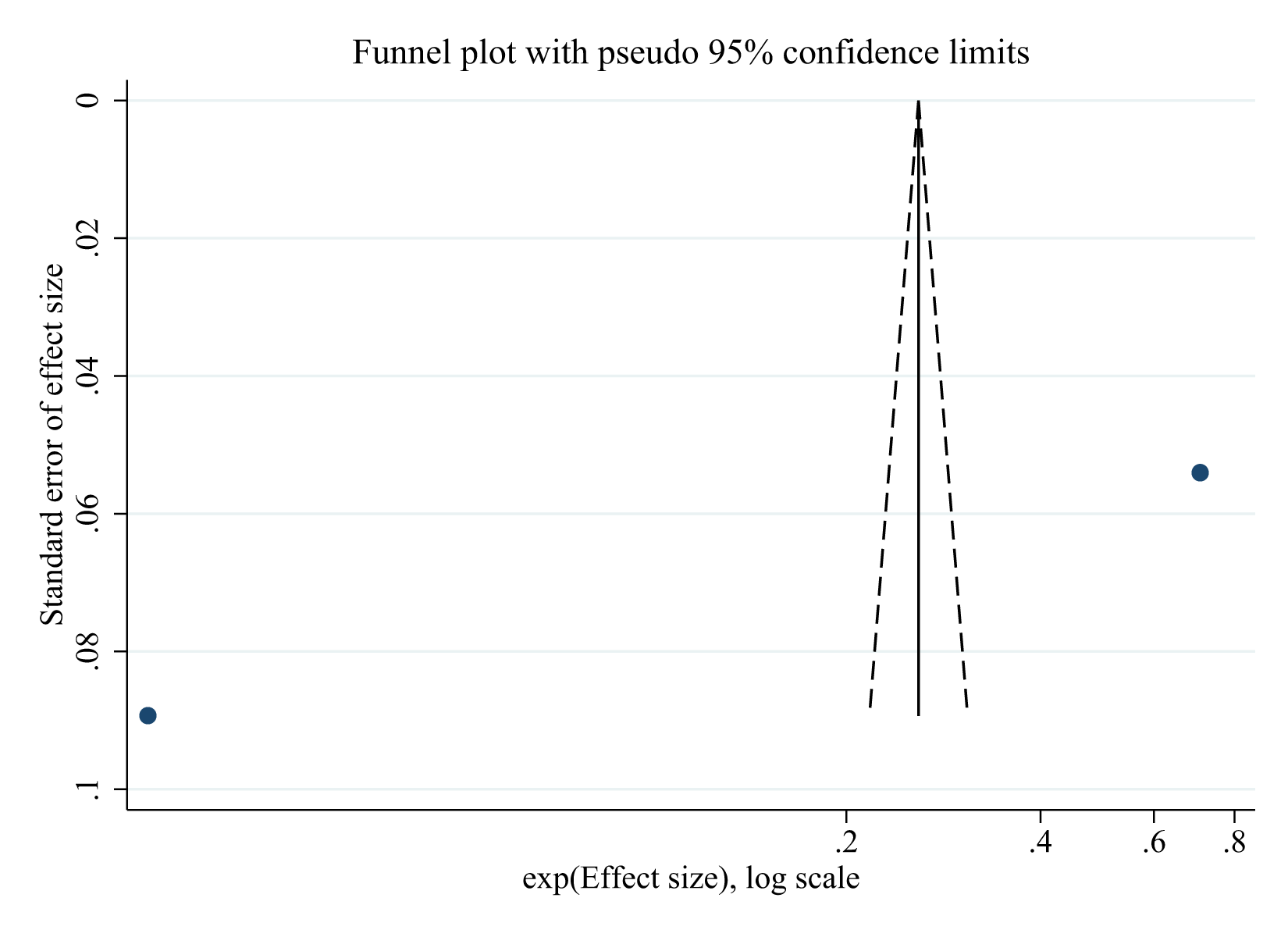

② HR


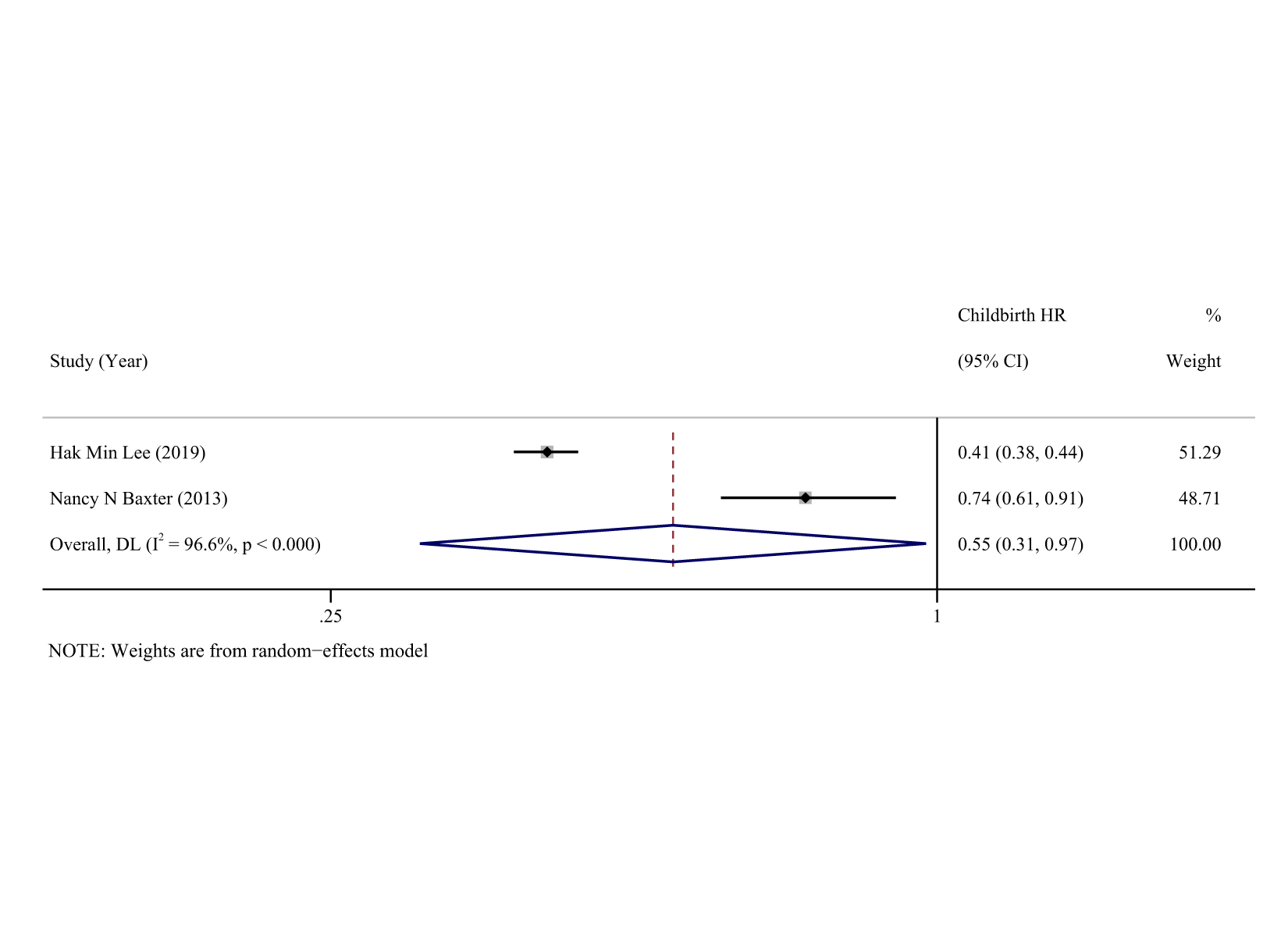


Random effect: p=0.041.

Egger’s test: not calculable


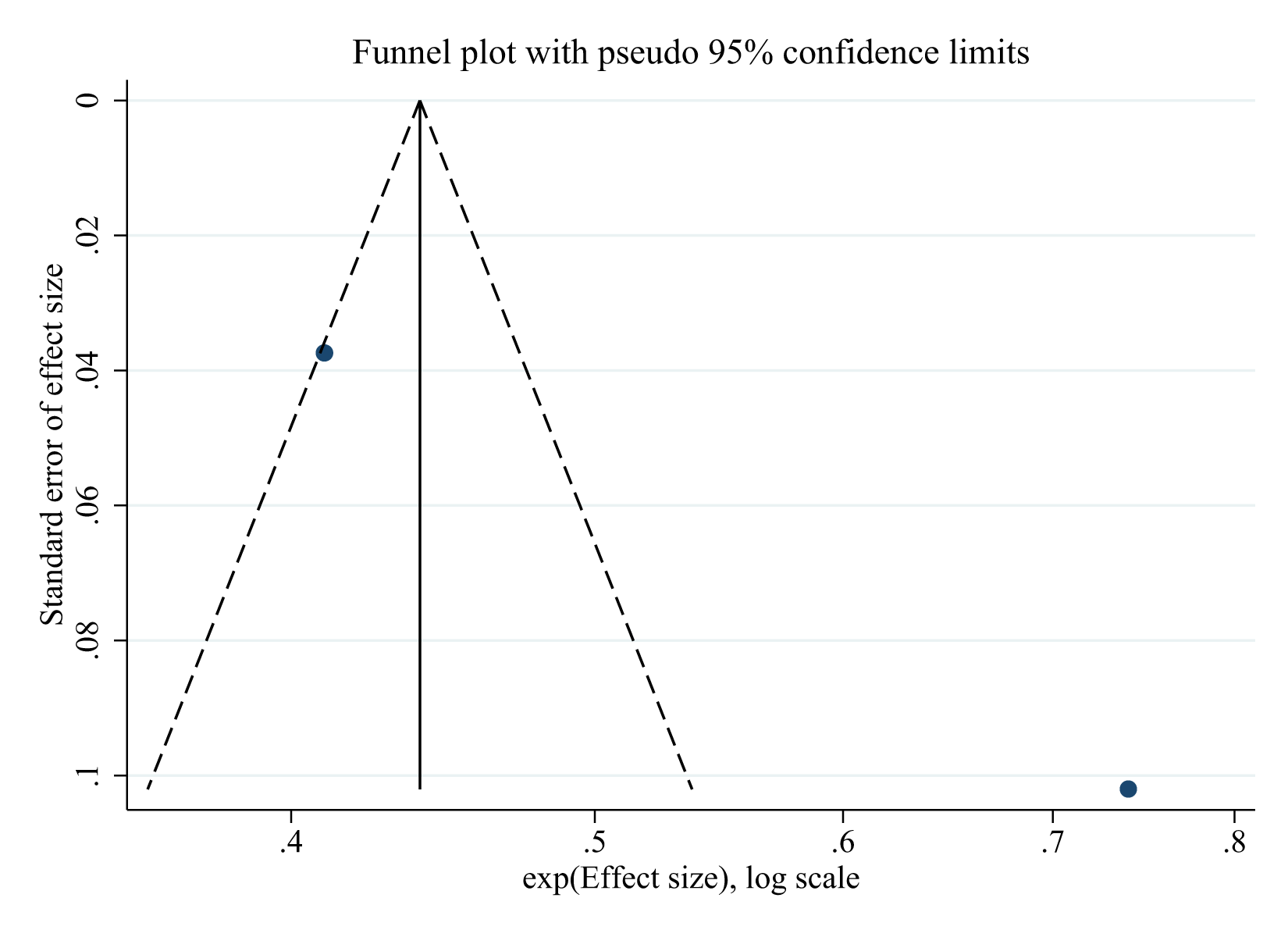

B) Assisted vaginal delivery


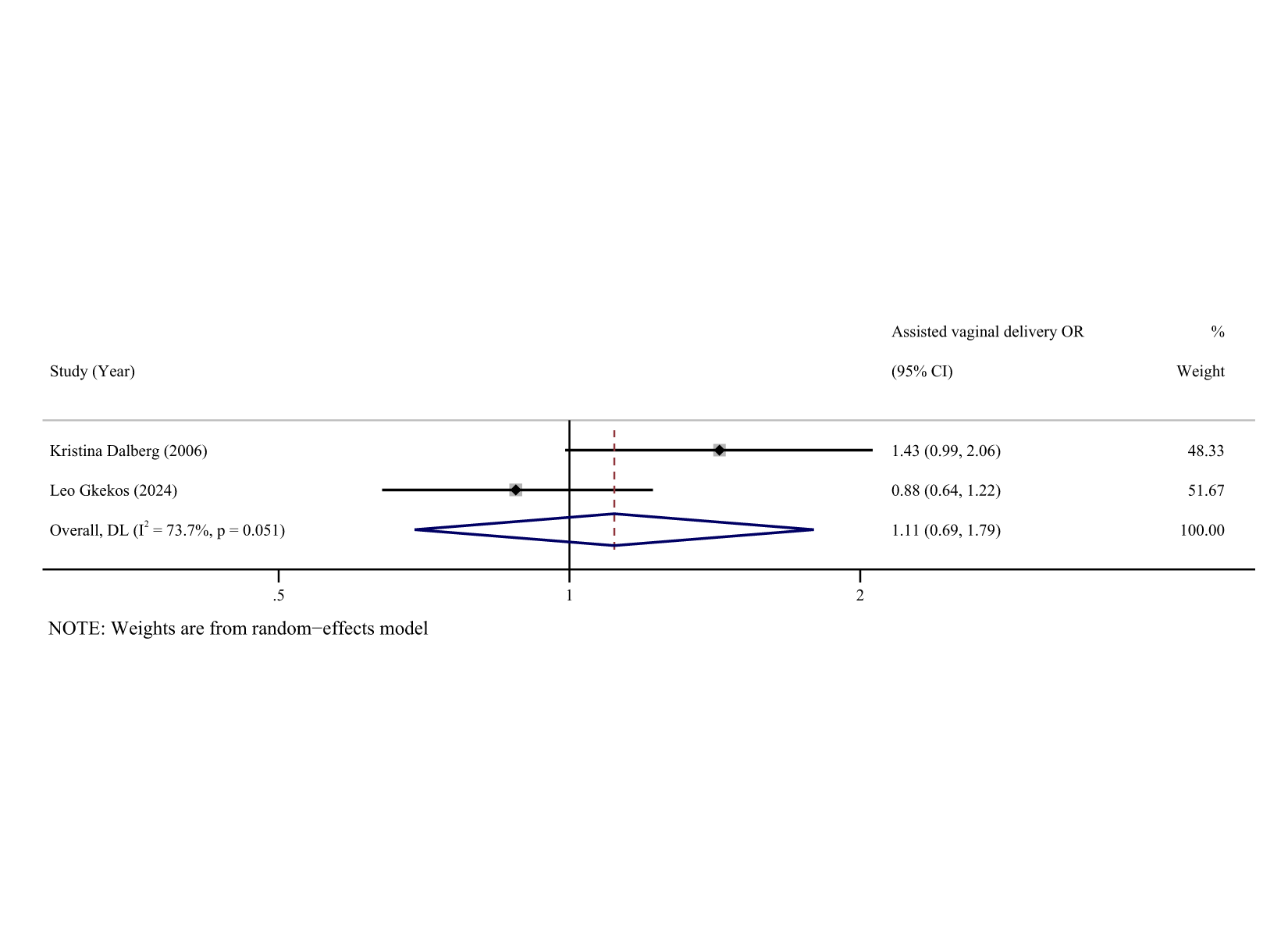


Random effect: p=0.660.

Egger’s test: not calculable


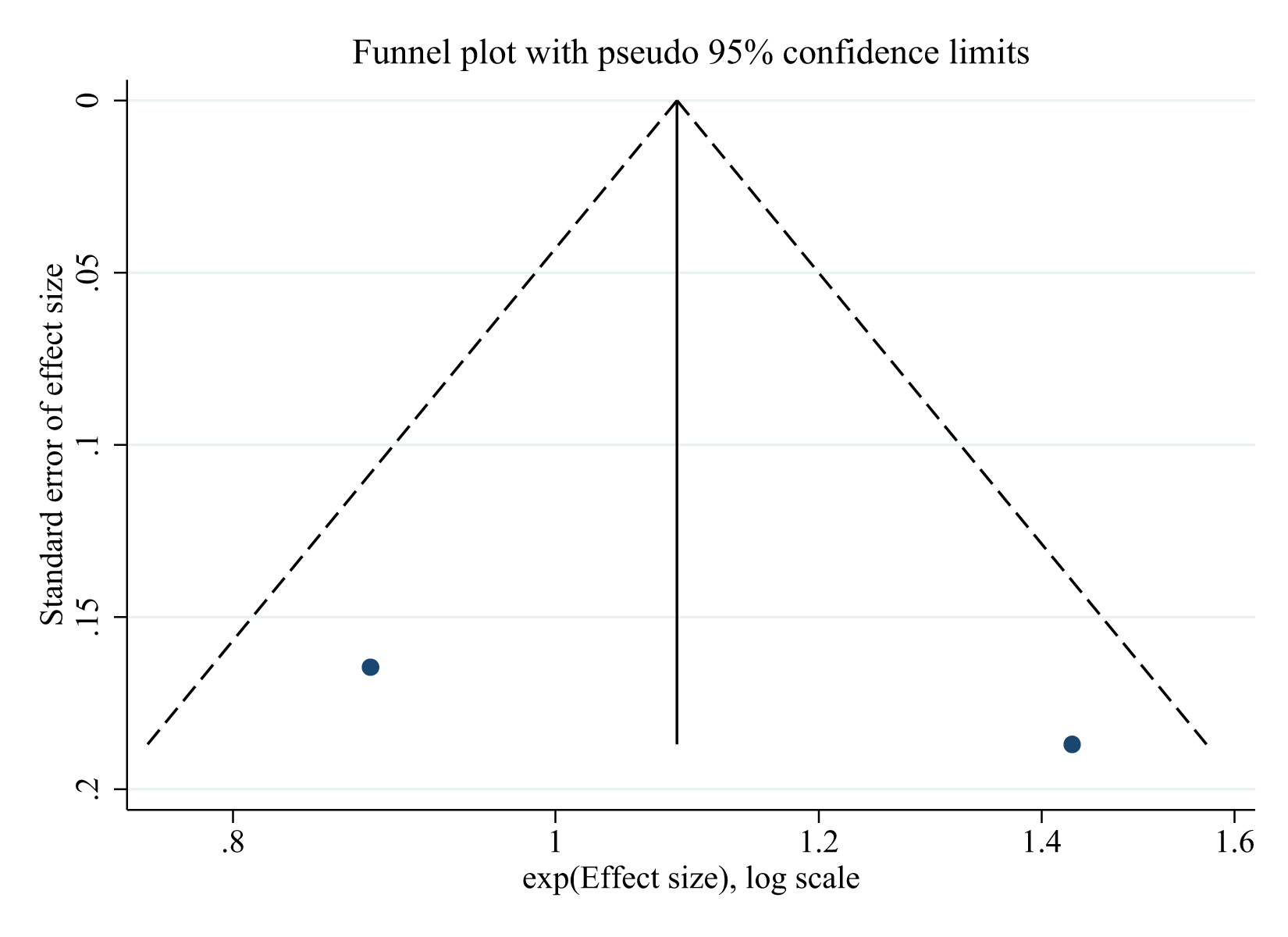

C) Cesarean delivery

① OR


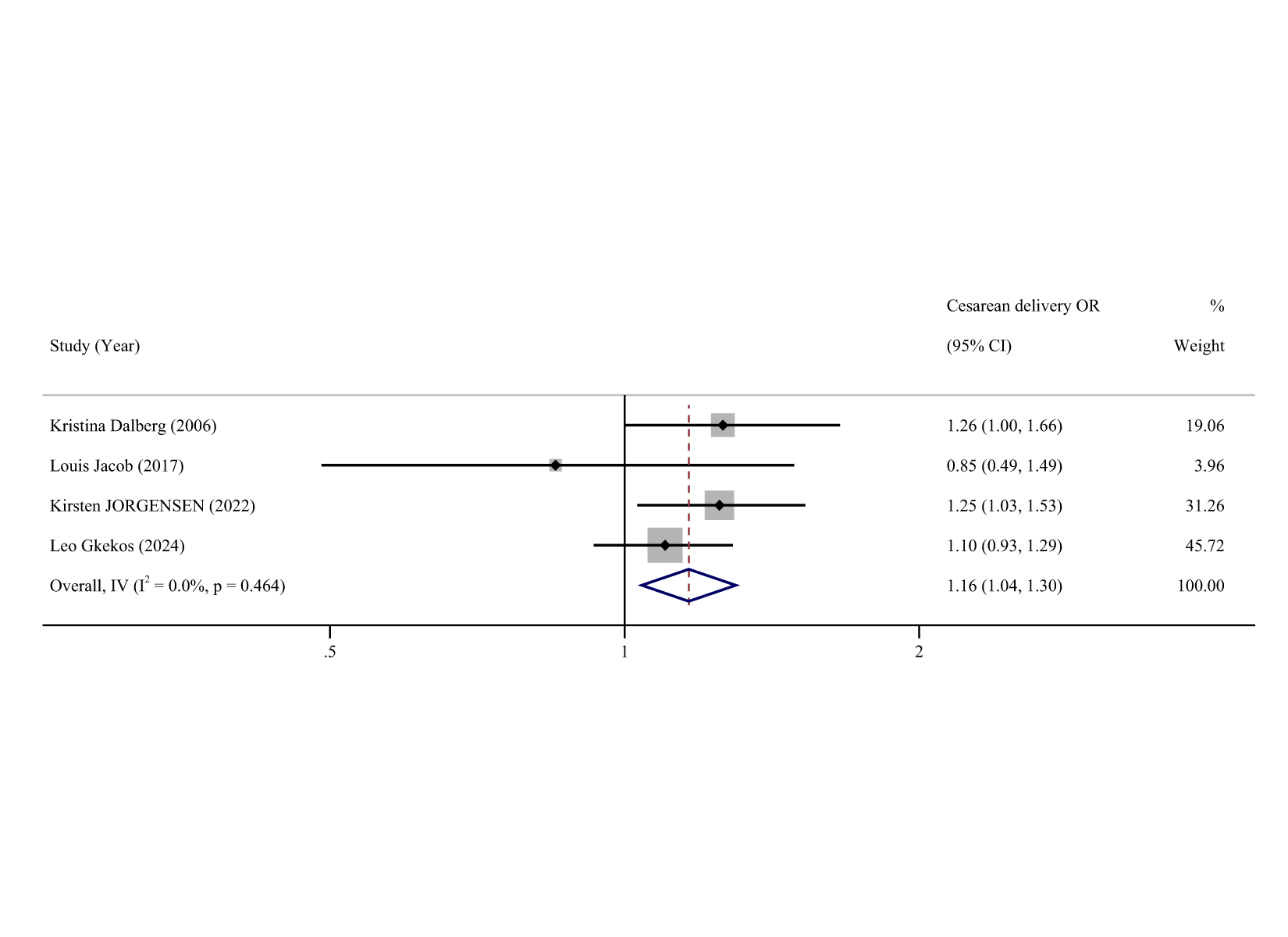


Random effect: p=0.007.

Egger’s test: p=0.614


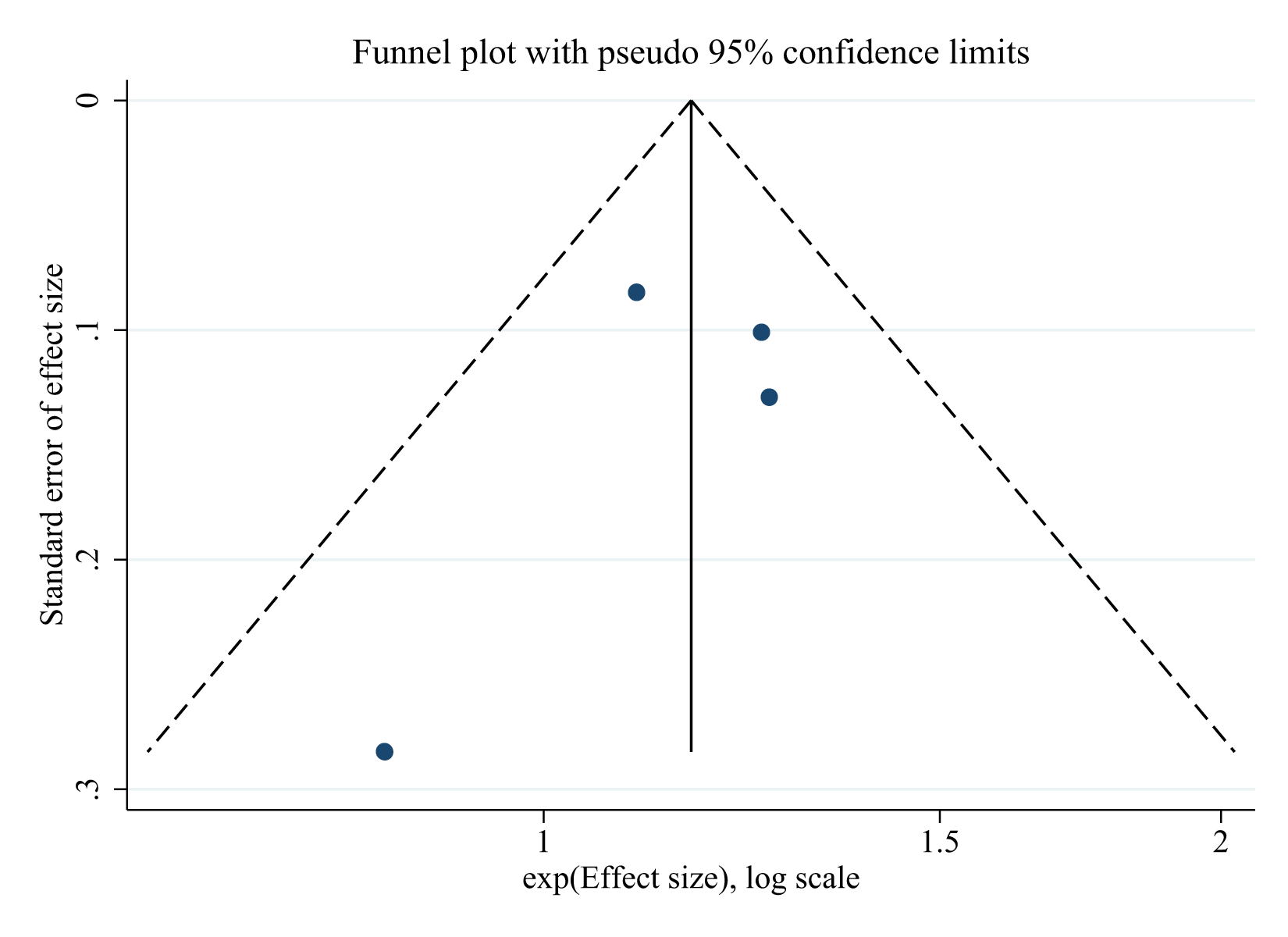

② RR


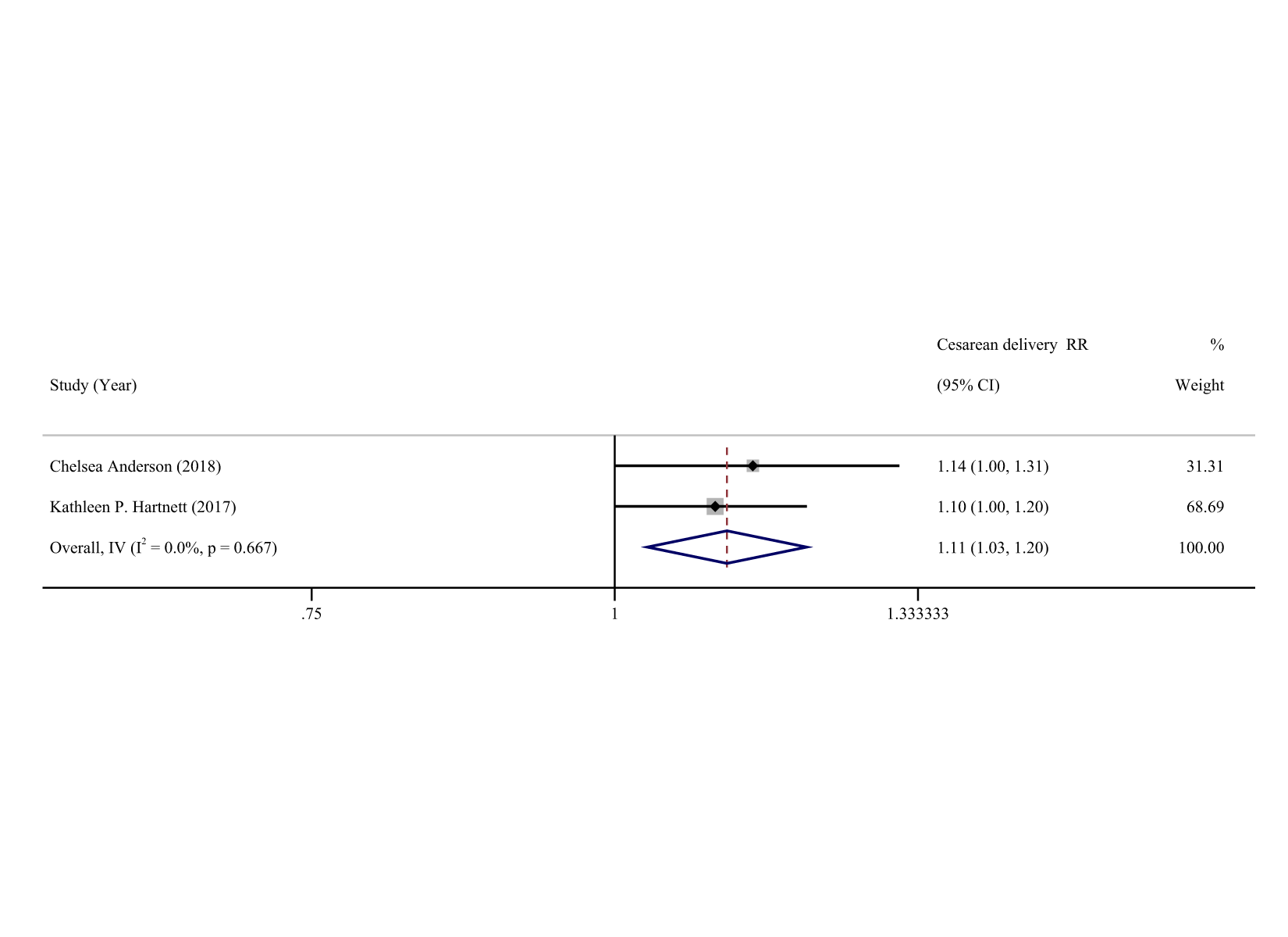


Random effect: p=0.006.

Egger’s test: not calculable


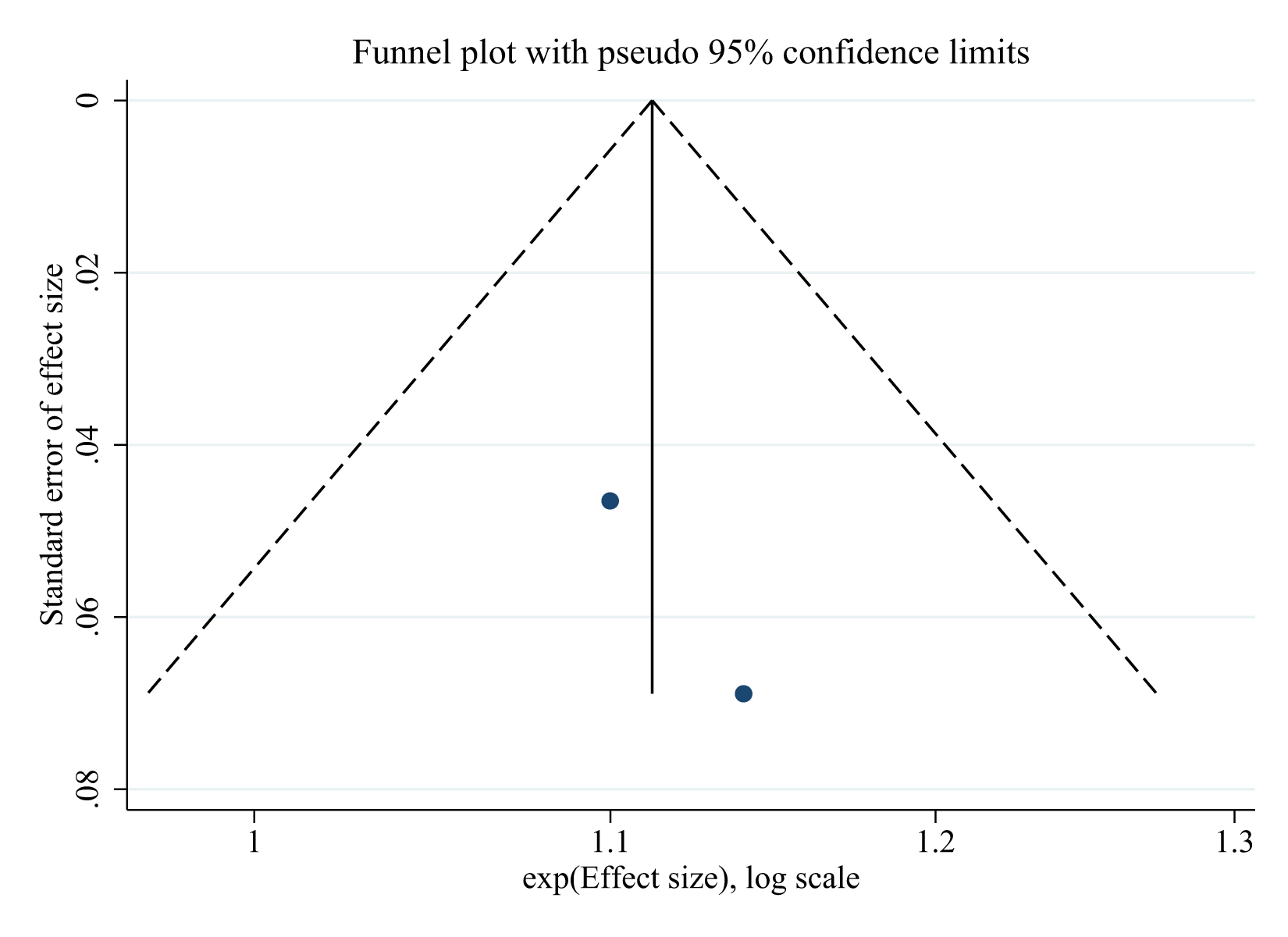

1. Birth trauma


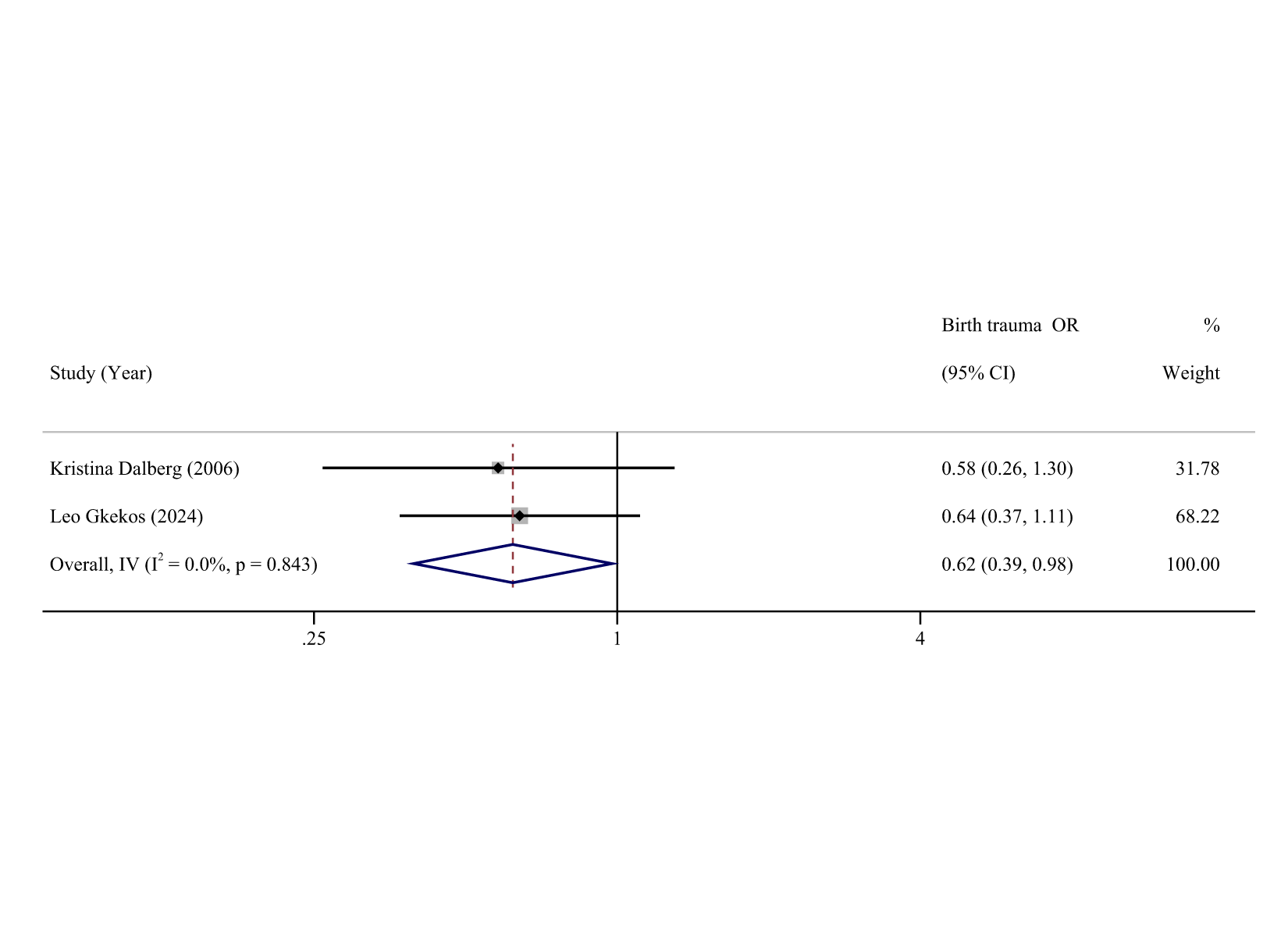


Random effect: p=0.039.

Egger’s test: not calculable


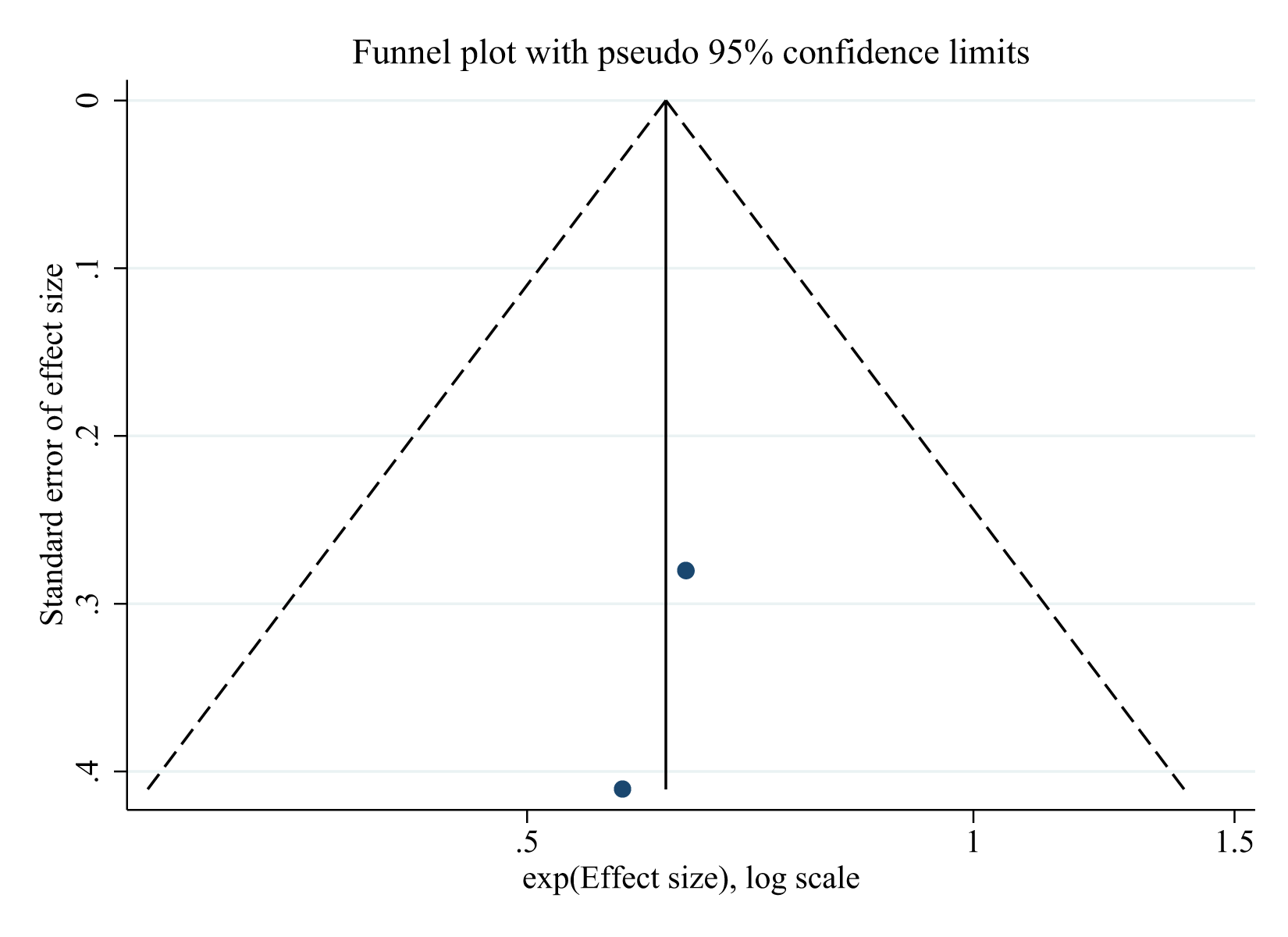

1. Preterm labor


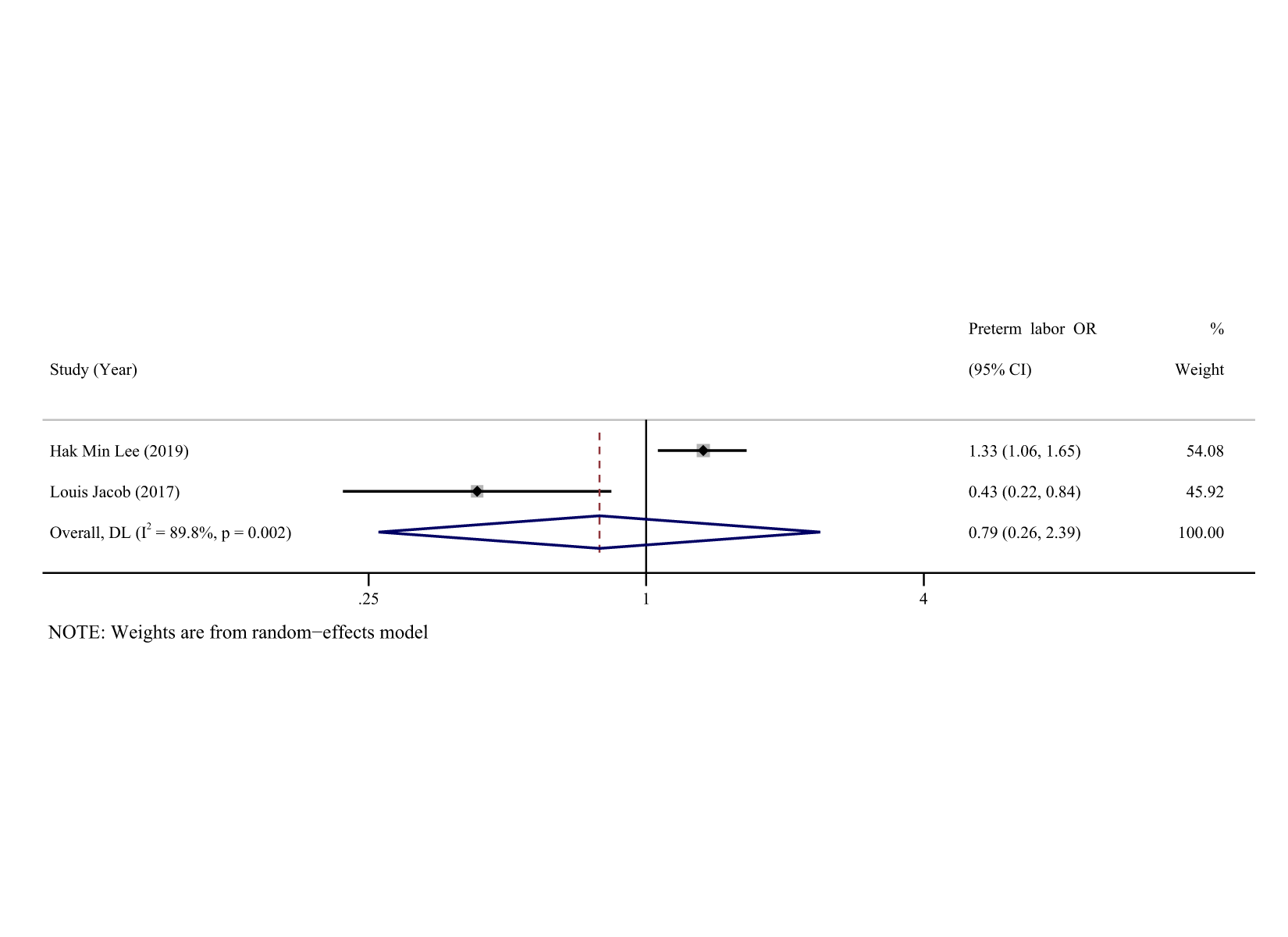


Random effect: p=0.678.

Egger’s test: not calculable


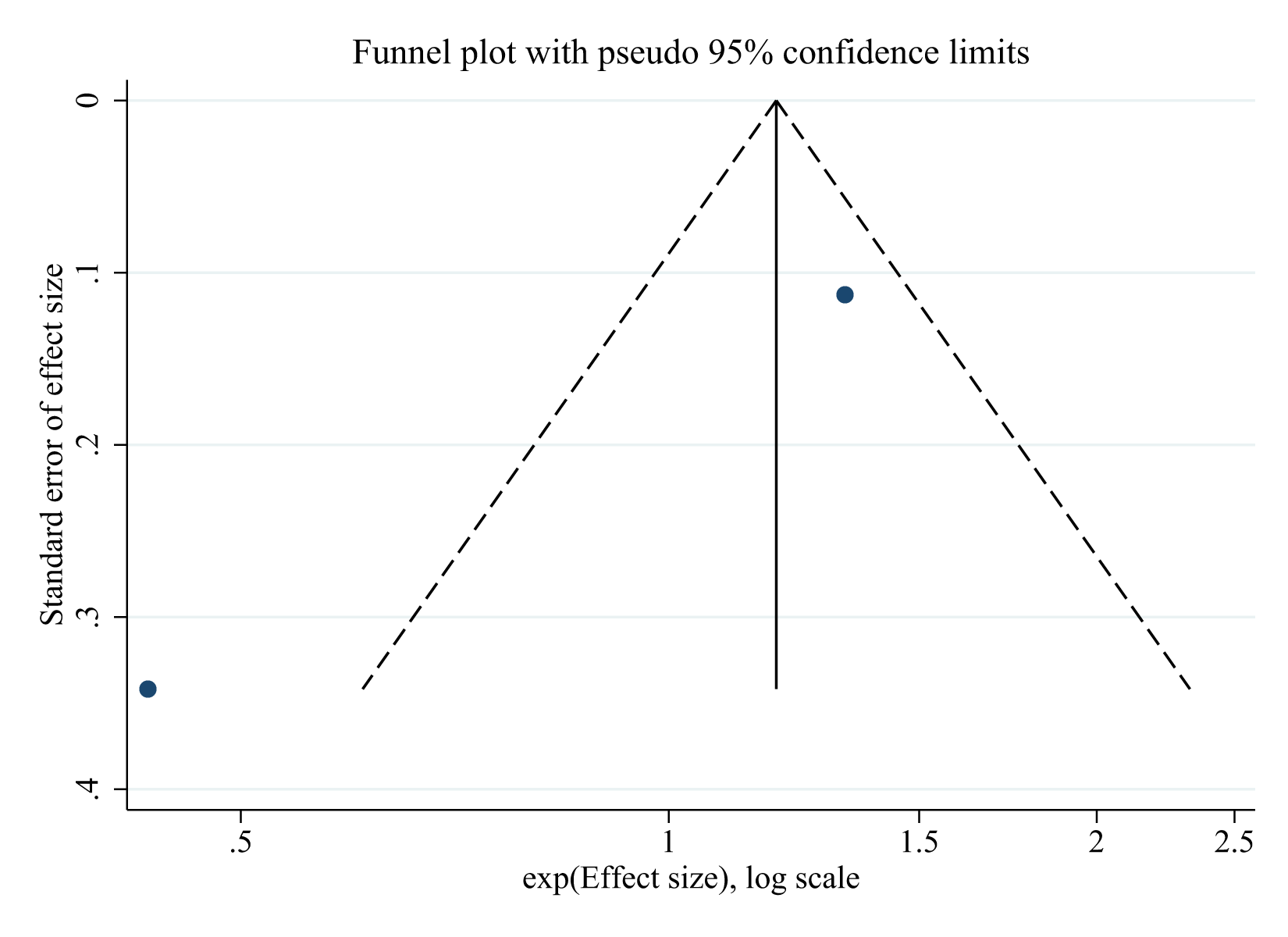

1. Very preterm birth(＜32 wk)


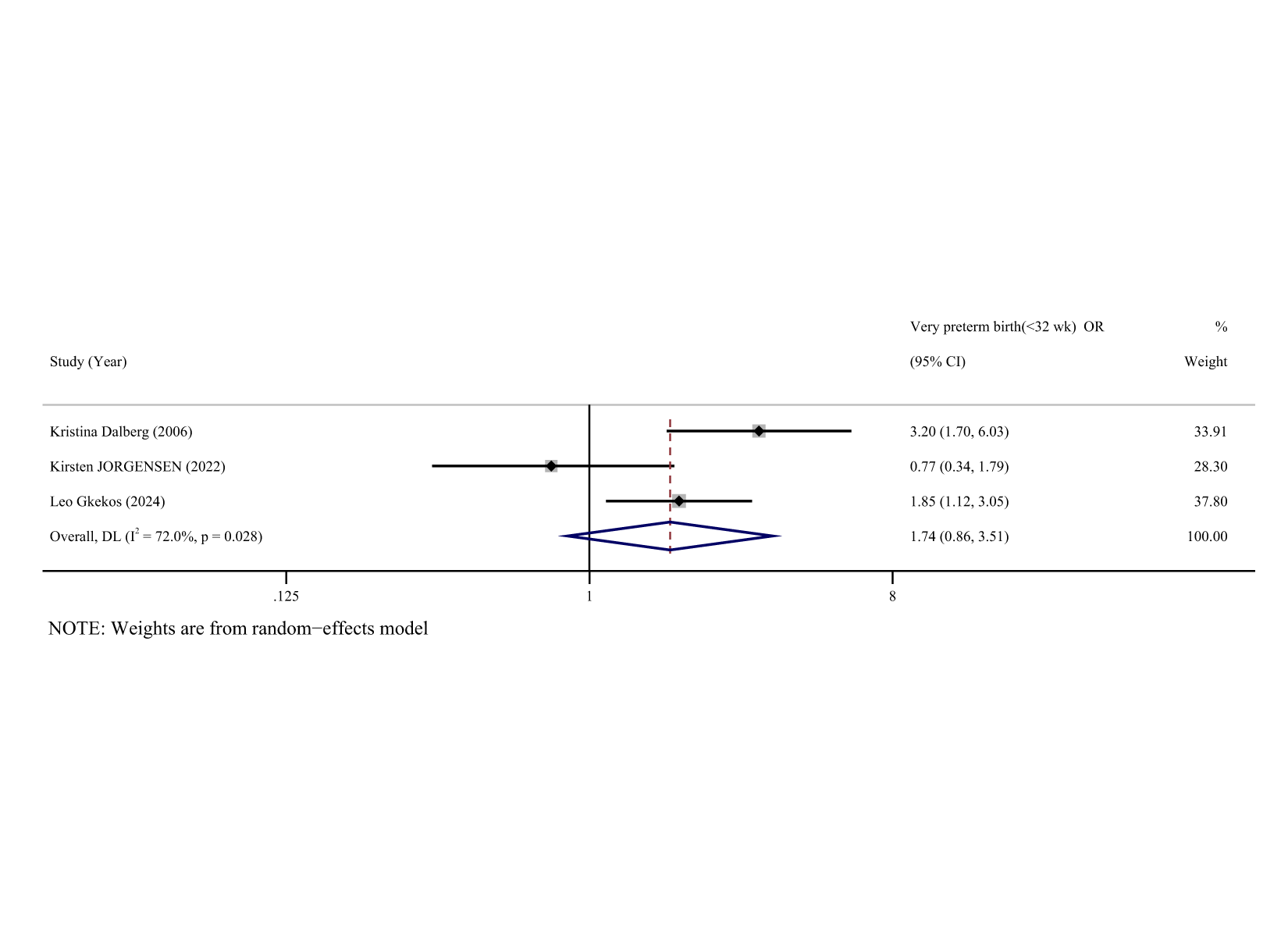


Random effect: p=0.123.

Egger’s test: p=0.646


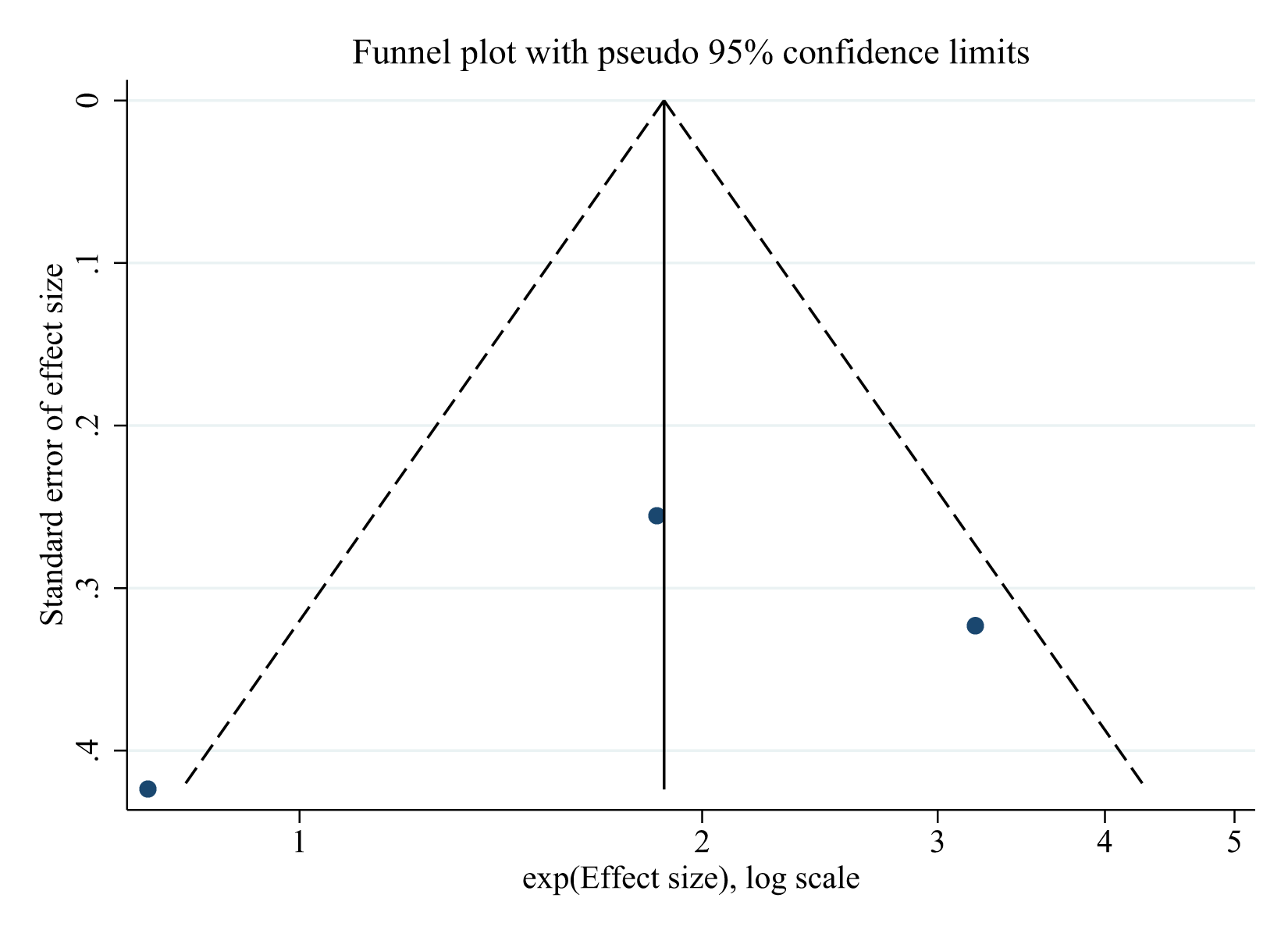

1. Preterm birth


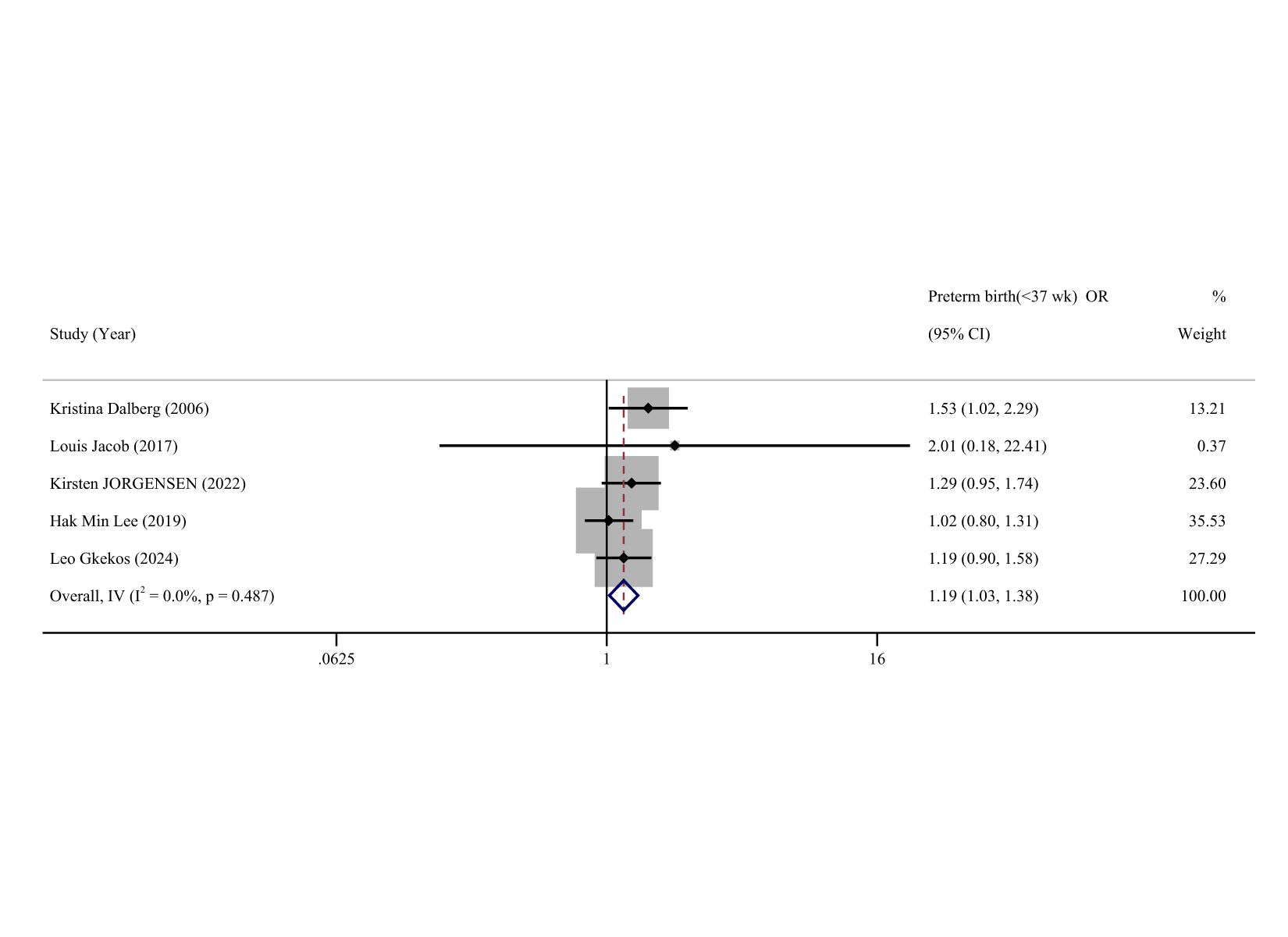


Random effect: p=0.021.

Egger’s test: p=0.338


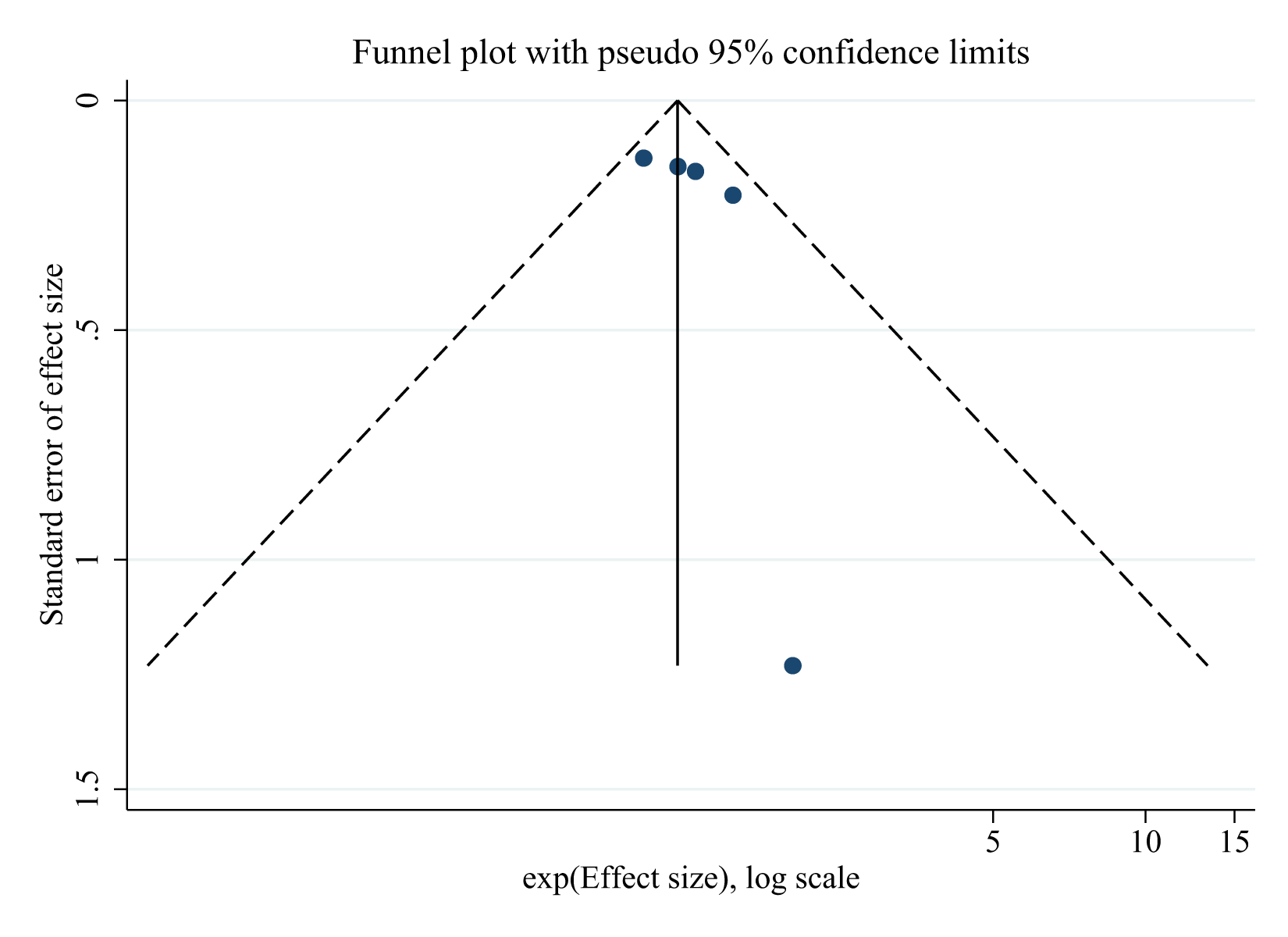

1. Preterm birth


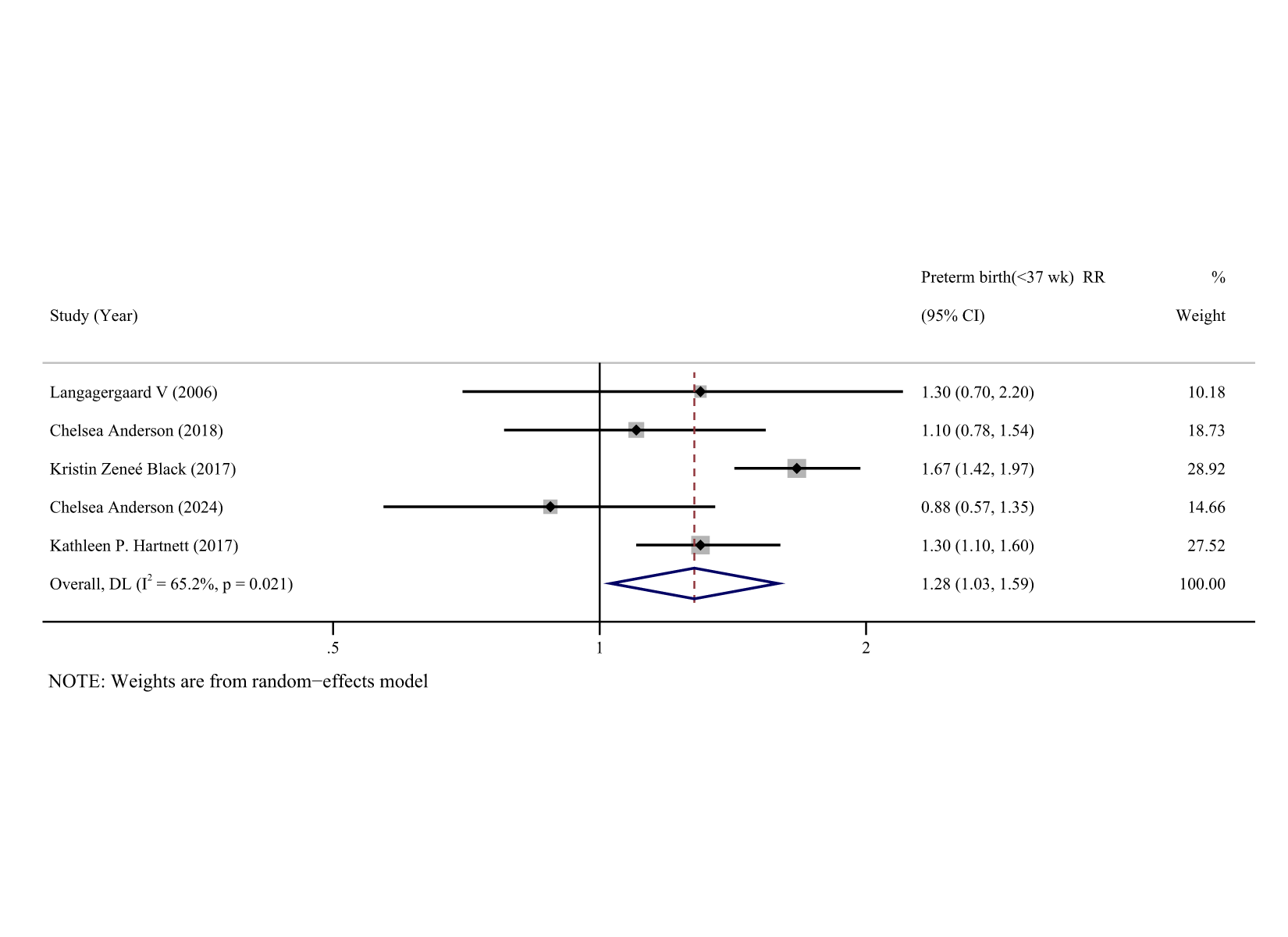


Random effect: p=0.026.

Egger’s test: p=0.180


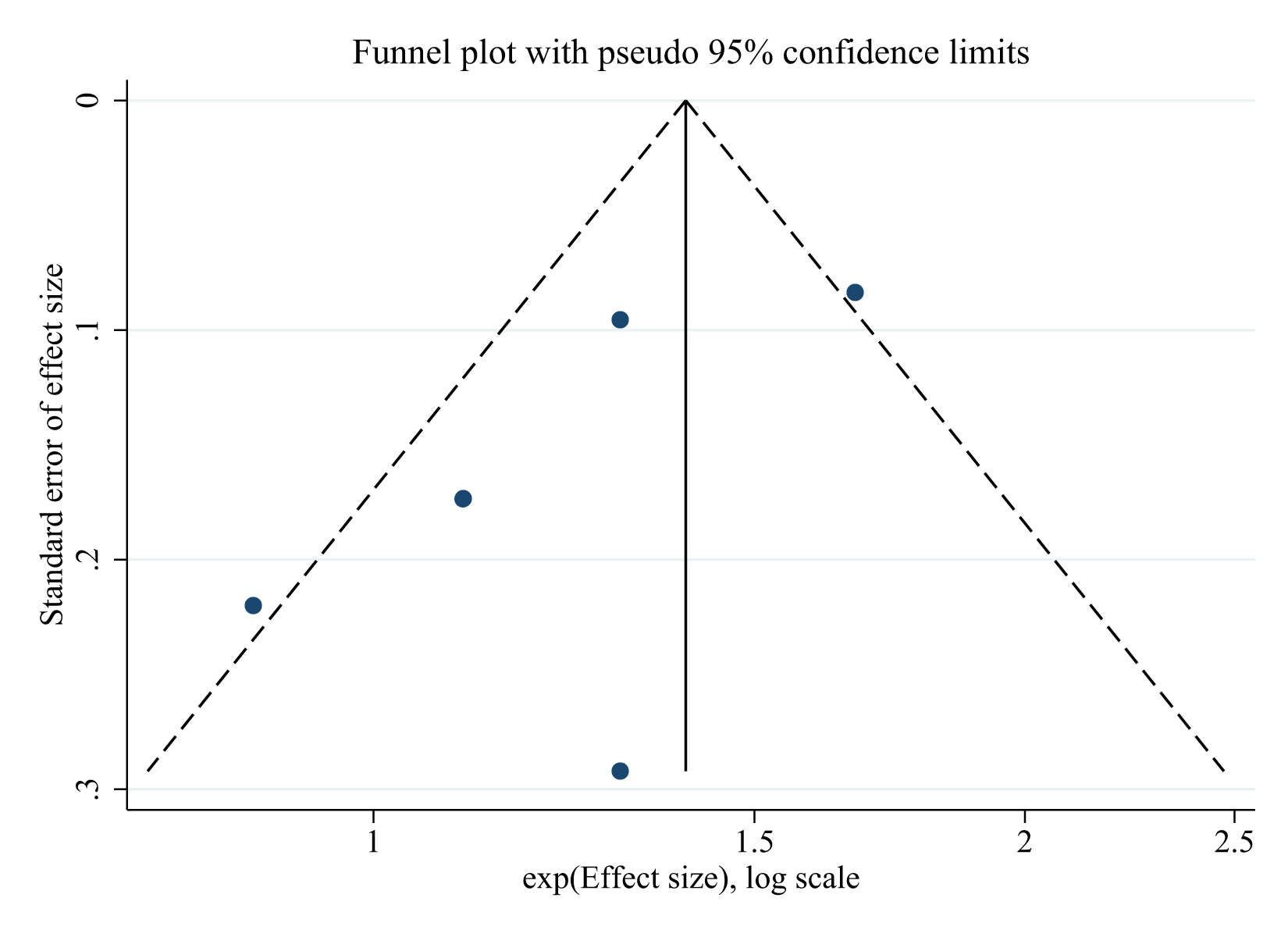

1. Multiple gestation


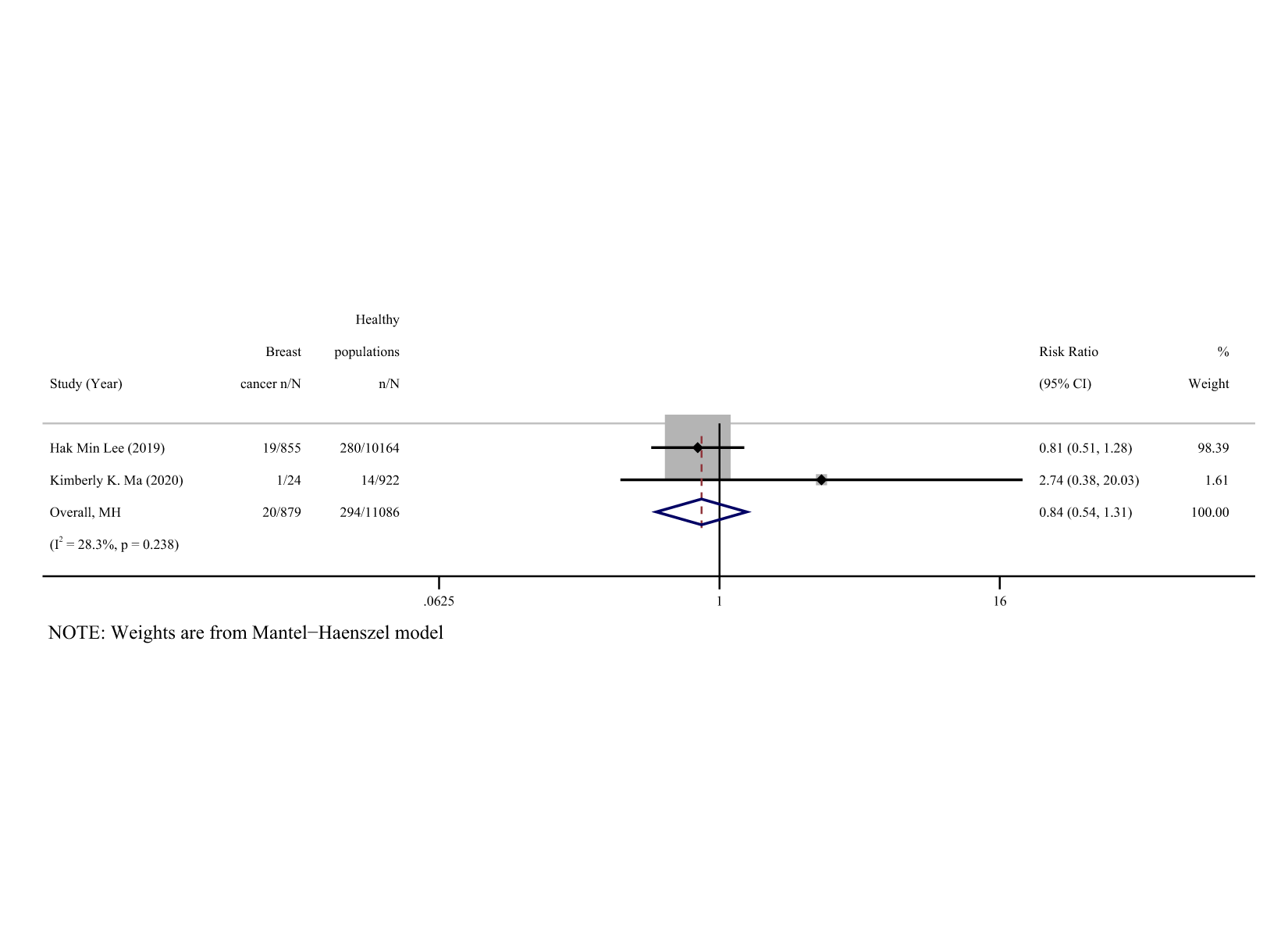


Random effect: p=0.438.

Egger’s test: not calculable


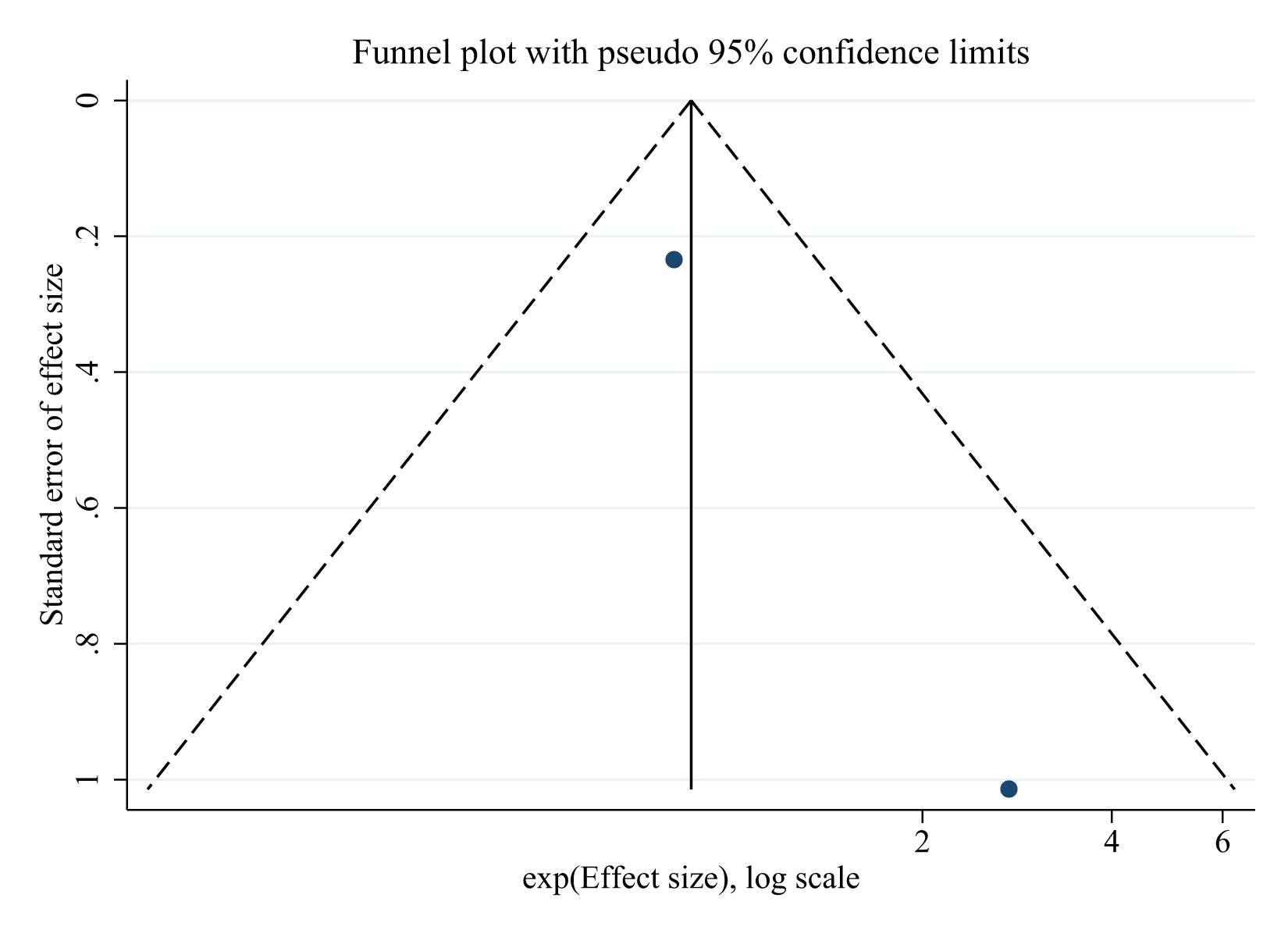

1. Premature rupture of membranes


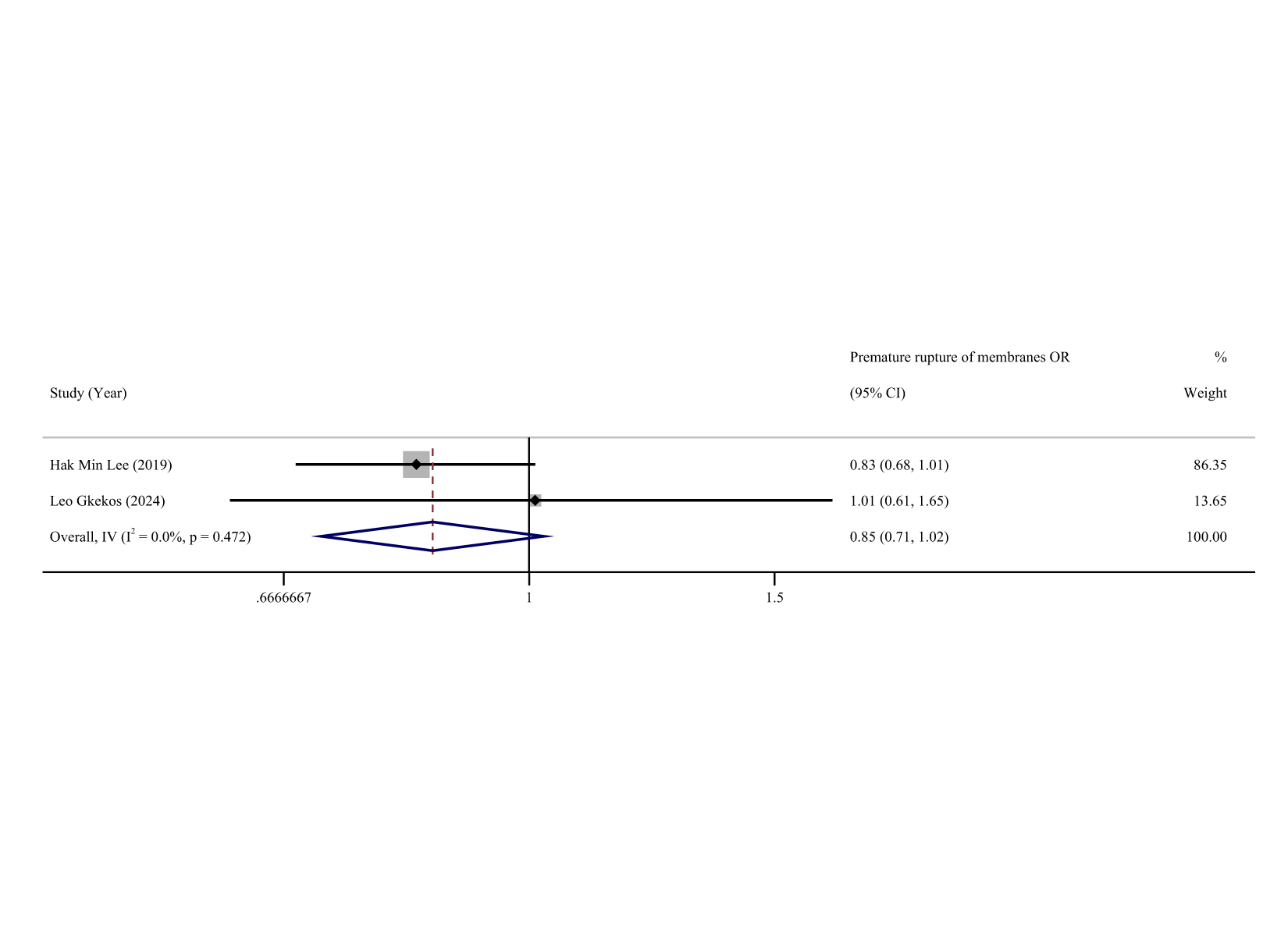


Random effect: p=0.089.

Egger’s test: not calculable


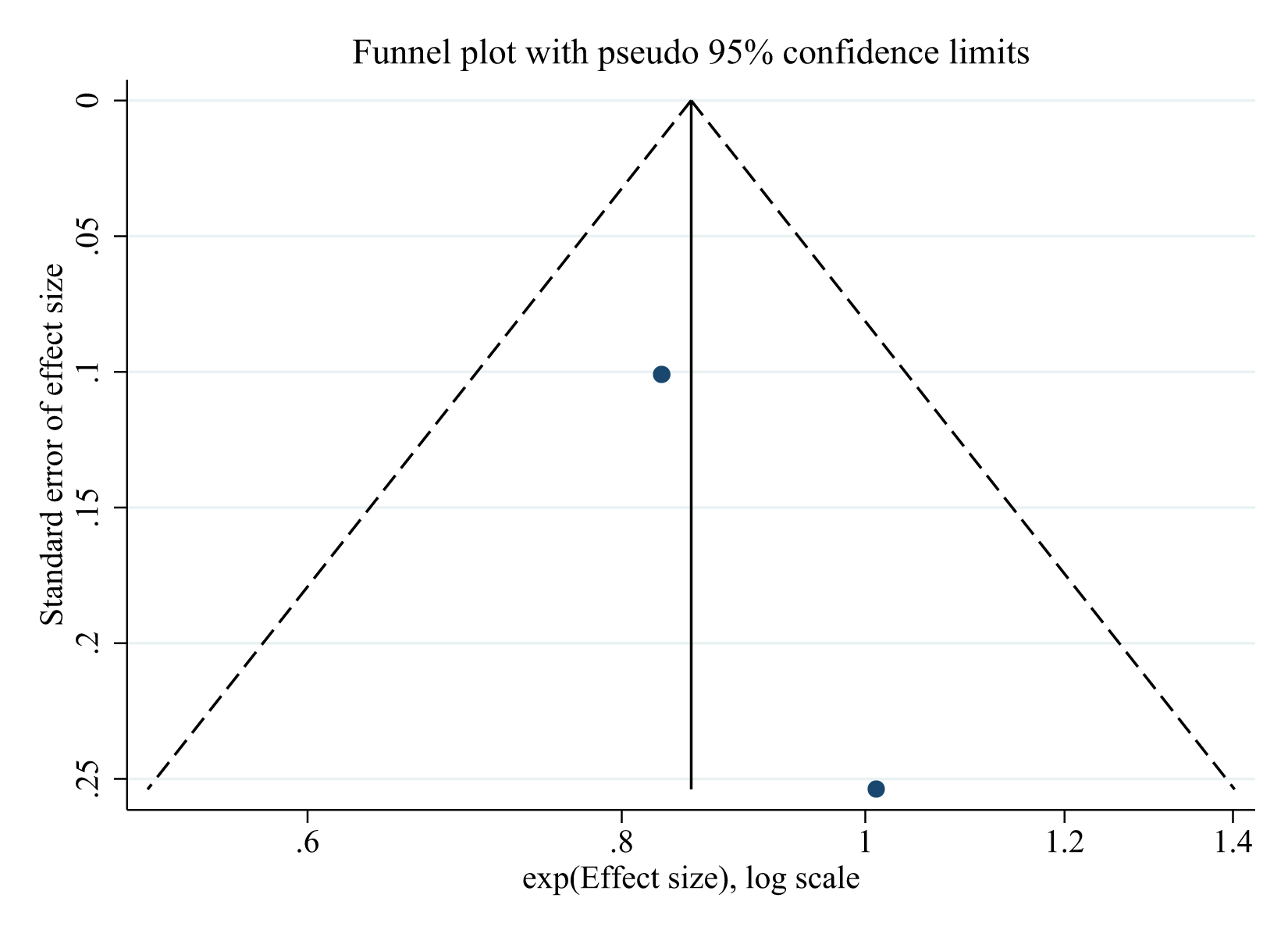
`

*Abbreviations: OR, odds ratio; RR, relative risk; HR, hazard ratio; CI, confidence intervals

**eFigure 3 Fetal outcomes comparing between breast cancer patients and healthy women from the general population.**

1. Very low birth weight (＜1500 g)
2. Binary variable


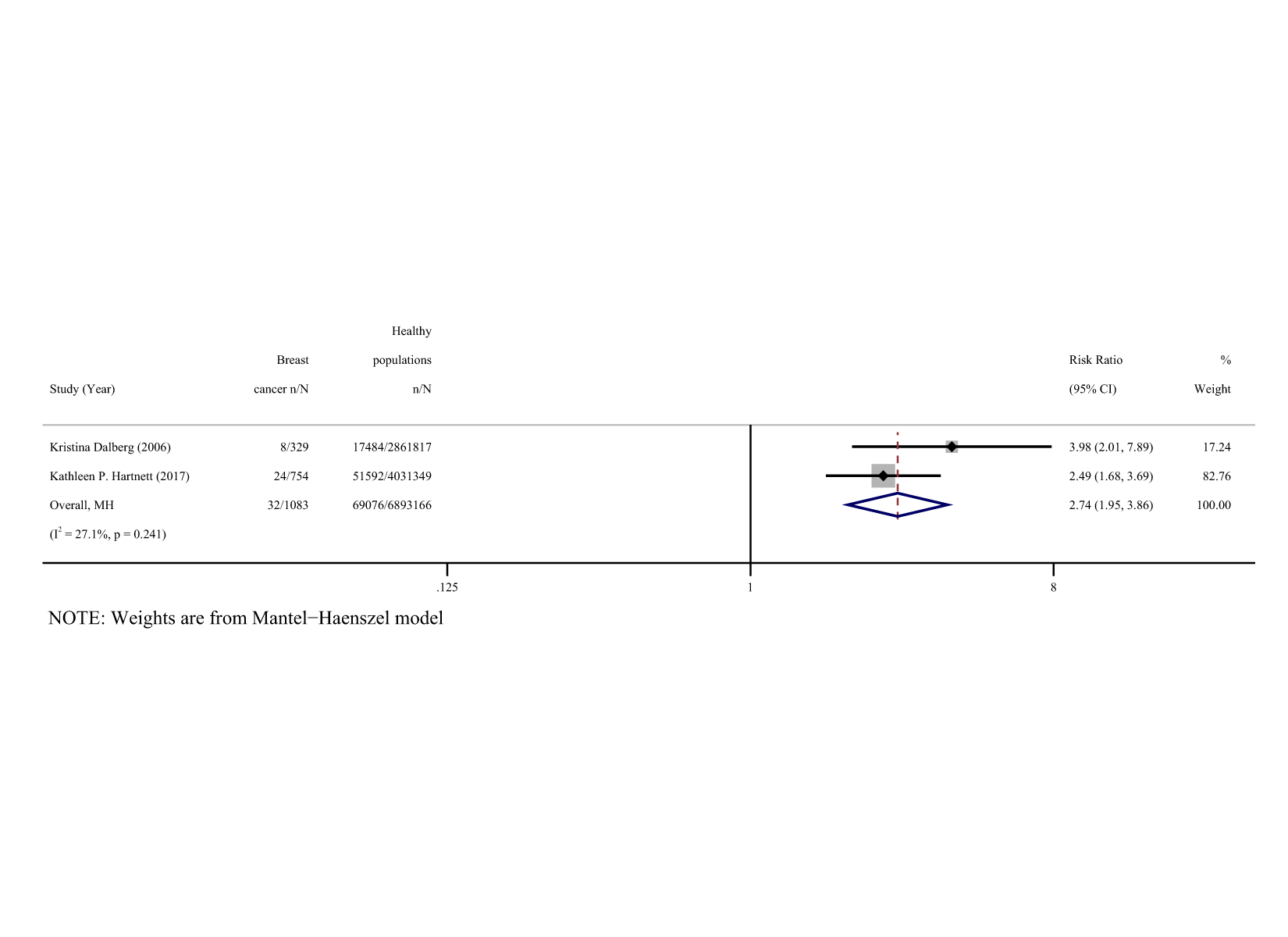


Random effect: p=0.000.

Egger’s test: not calculable.


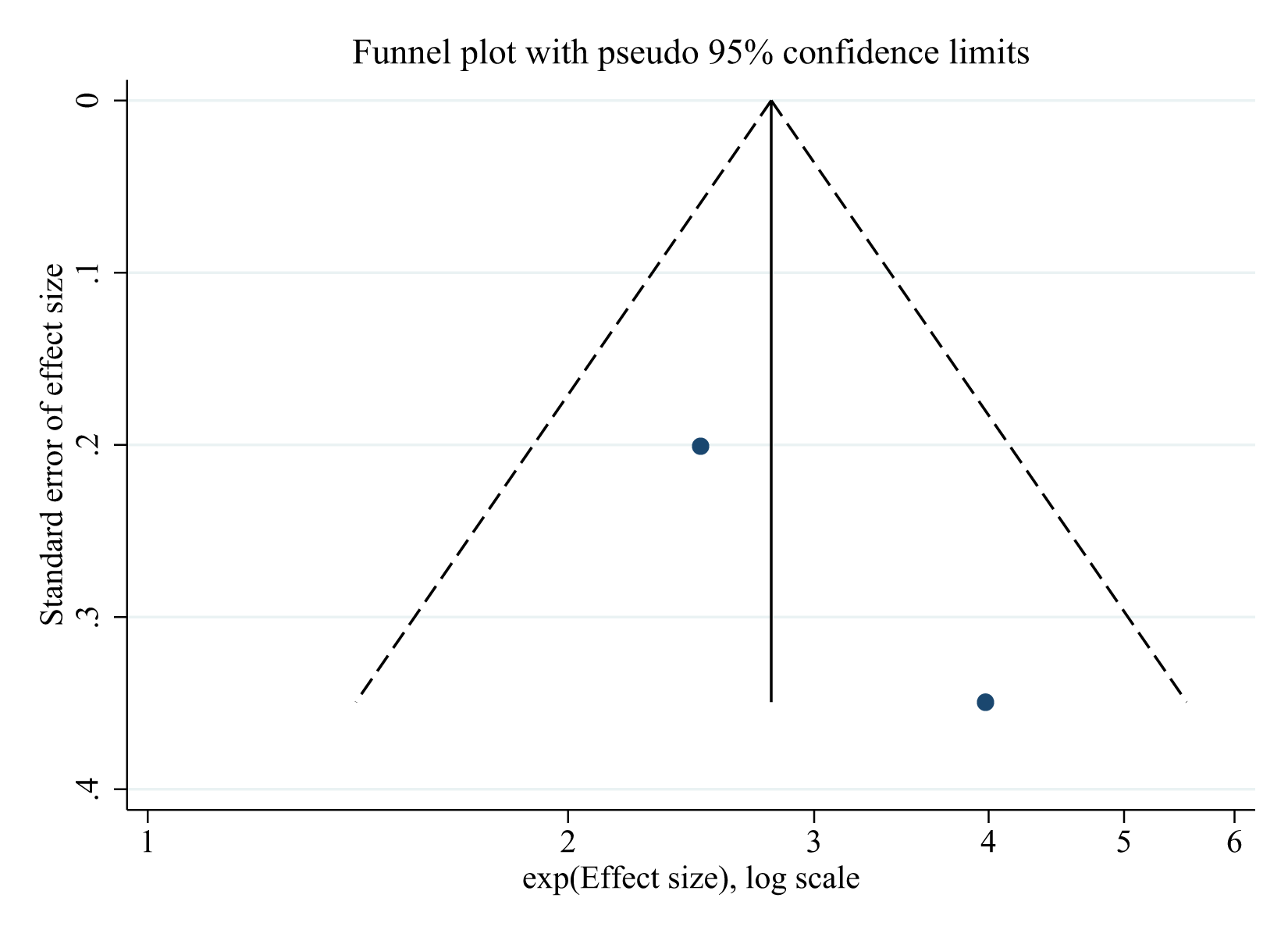

1. Low birth weight (＜2500 g)
2. RR


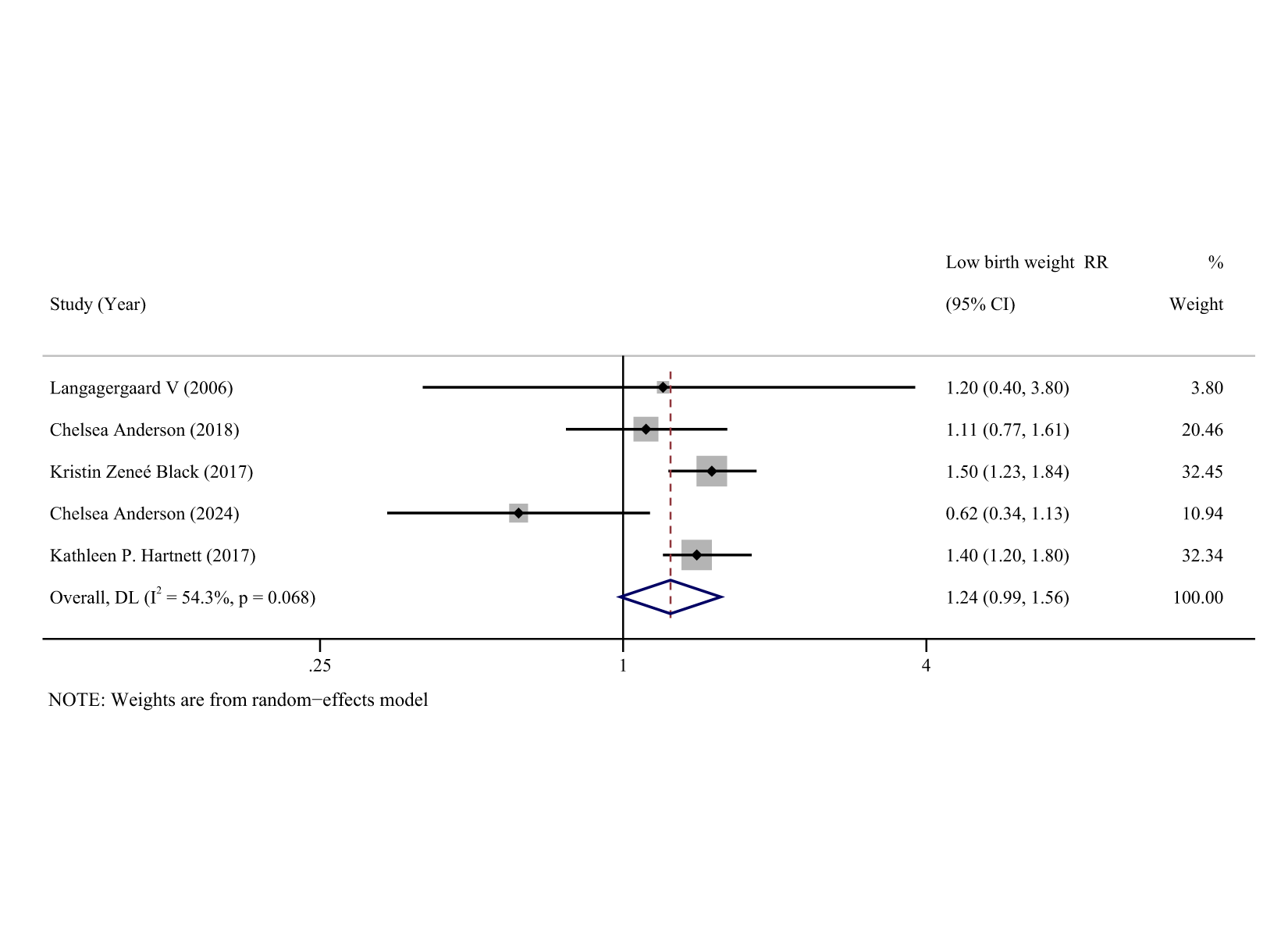


Random effect: p=0.065.

Egger’s test: p=0.167


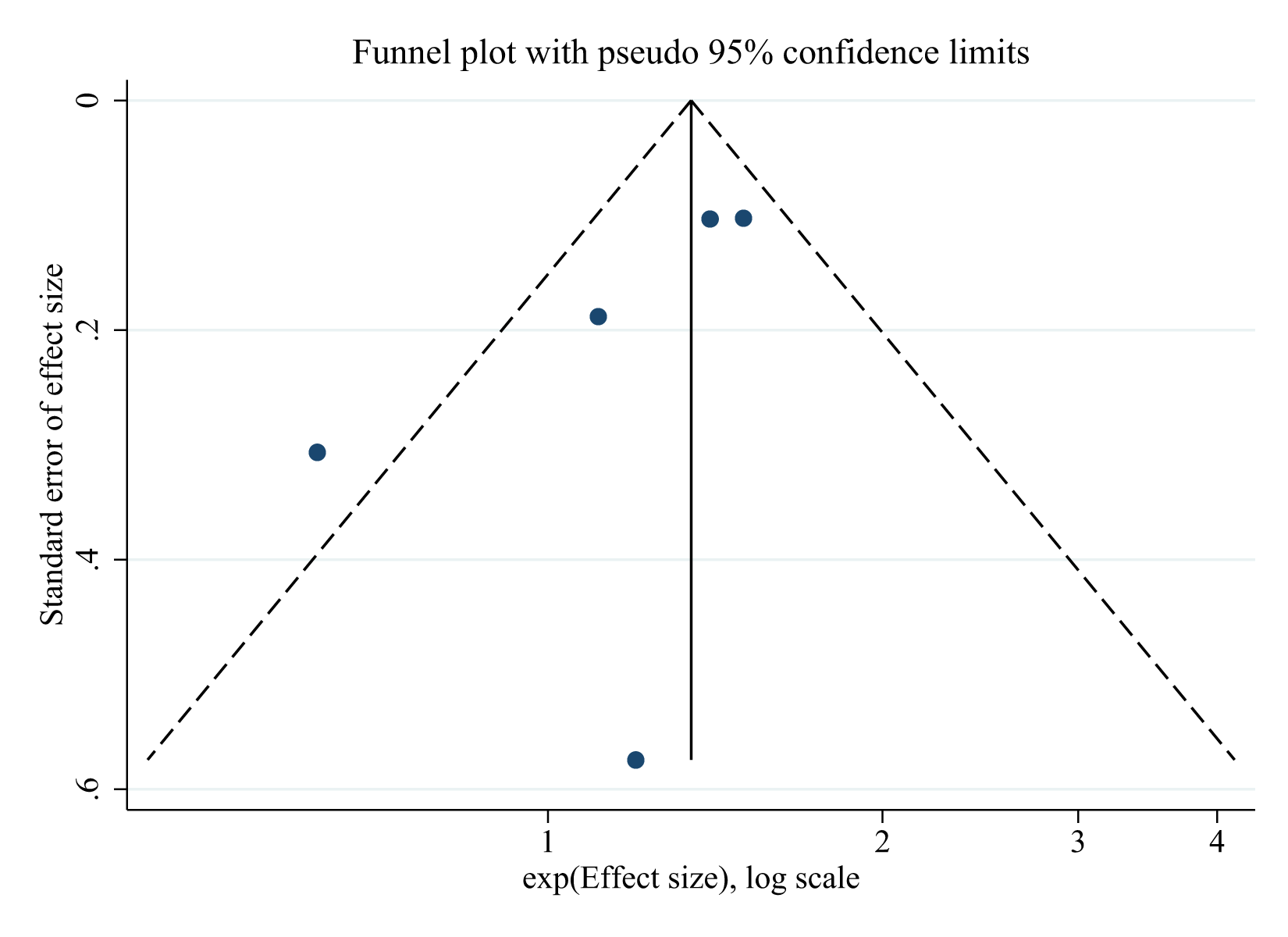

1. Binary variable


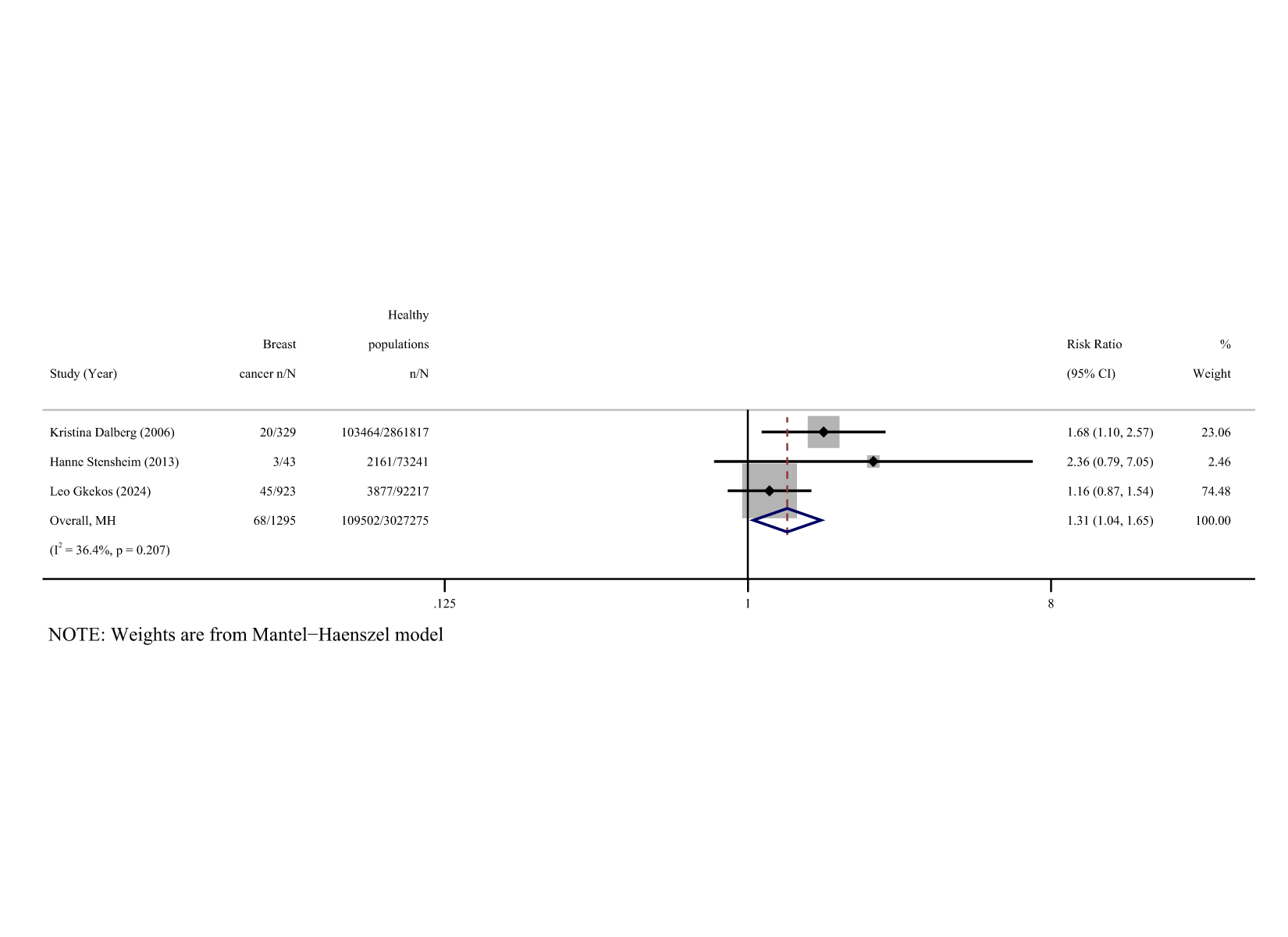


Random effect: p=0.023.

Egger’s test: p=0.357


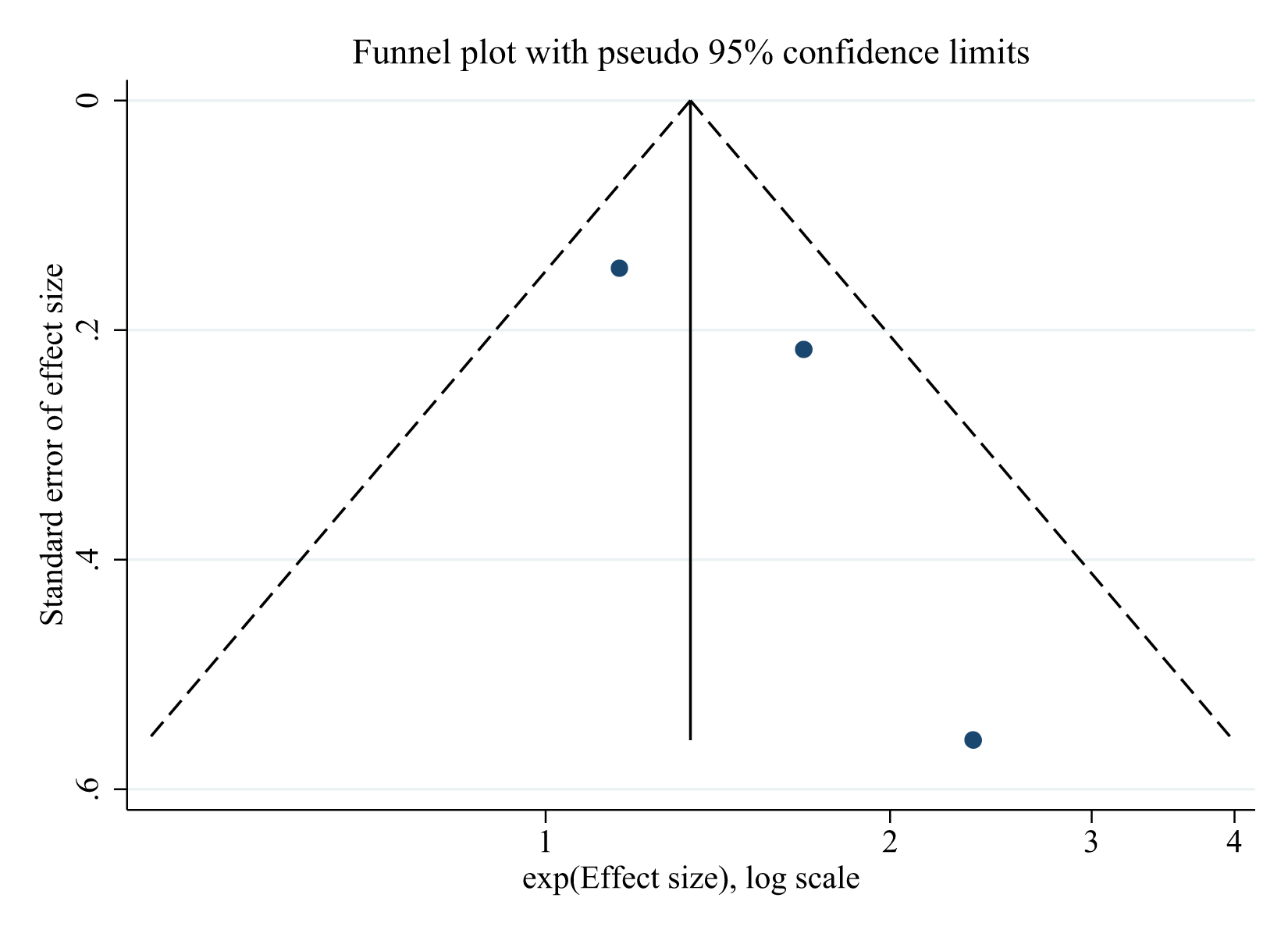

1. Low birth weight at term (＜2500 g)


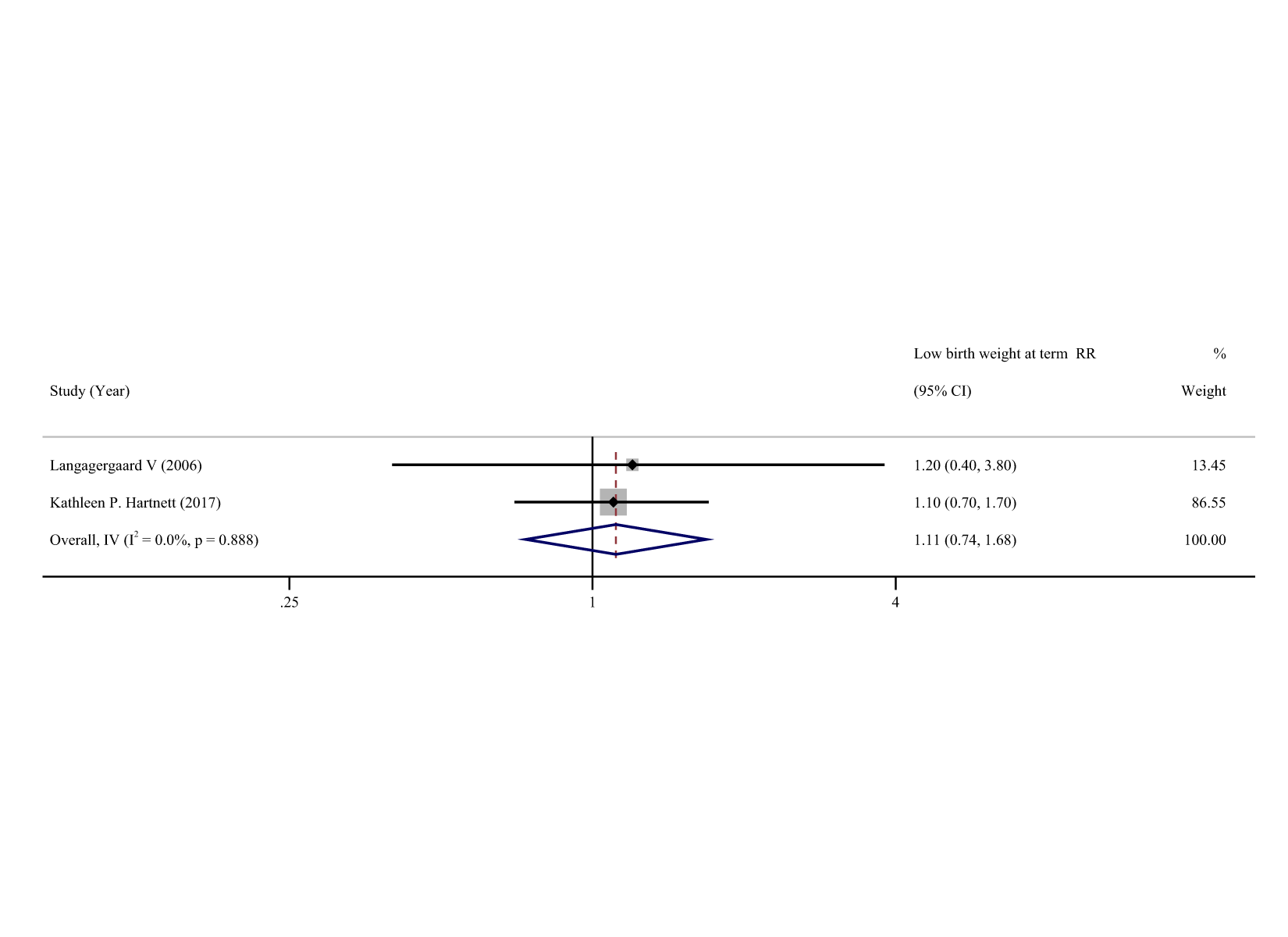


Random effect: p=0.611.

Egger’s test: not calculable


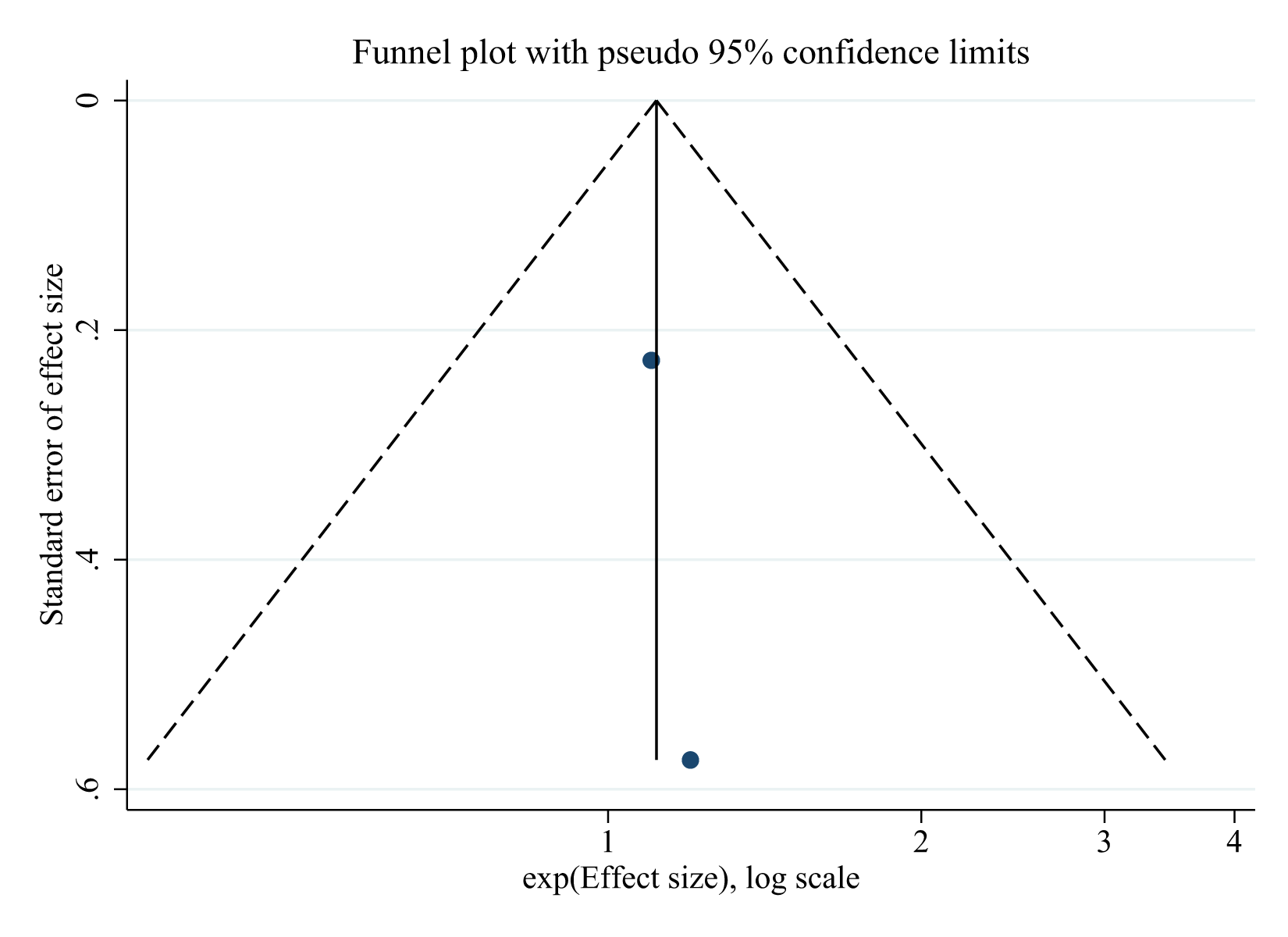

1. Small for gestational age (SGA)

① RR


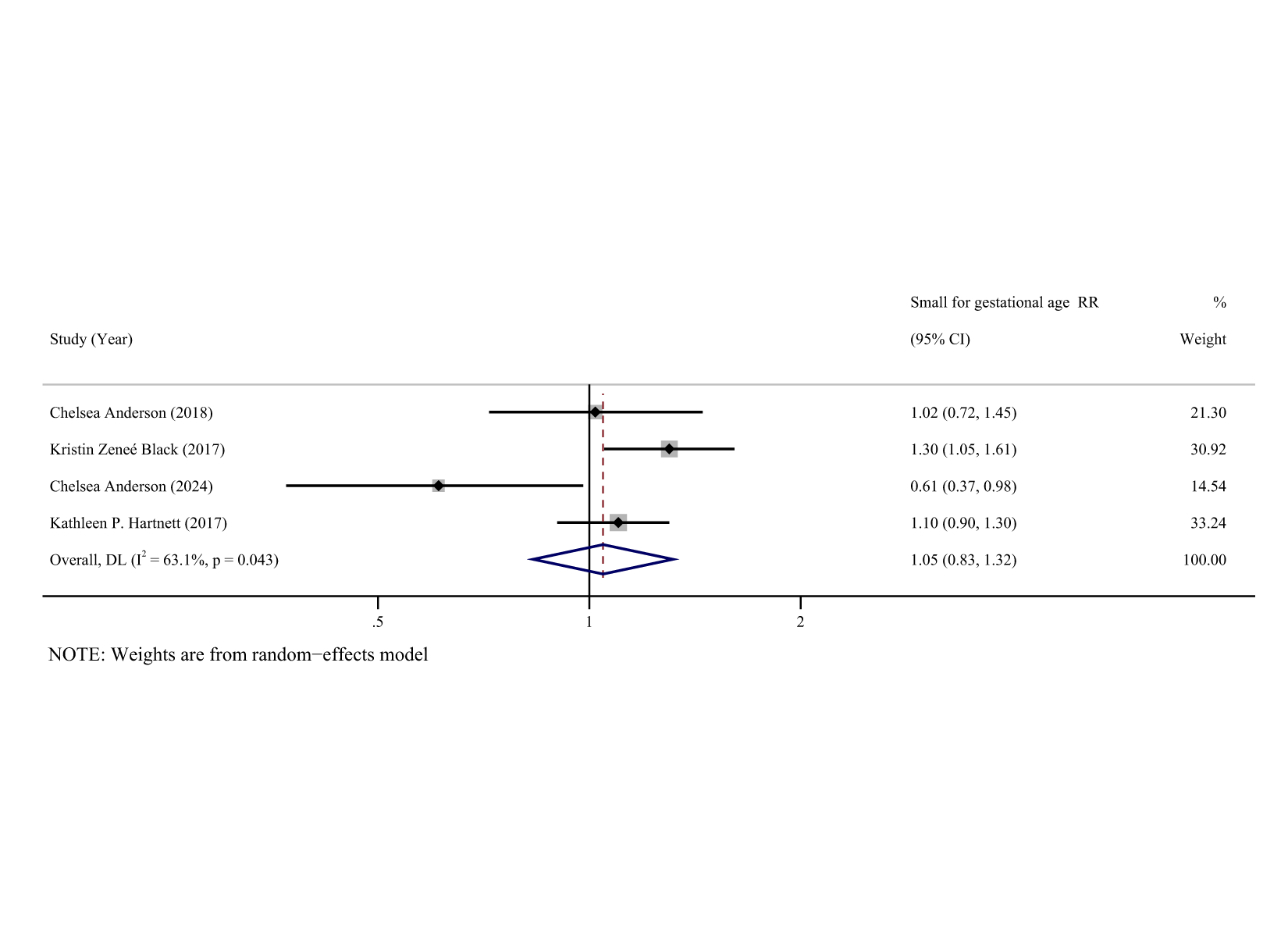


Random effect: p=0.700.

Egger’s test: p=0.217


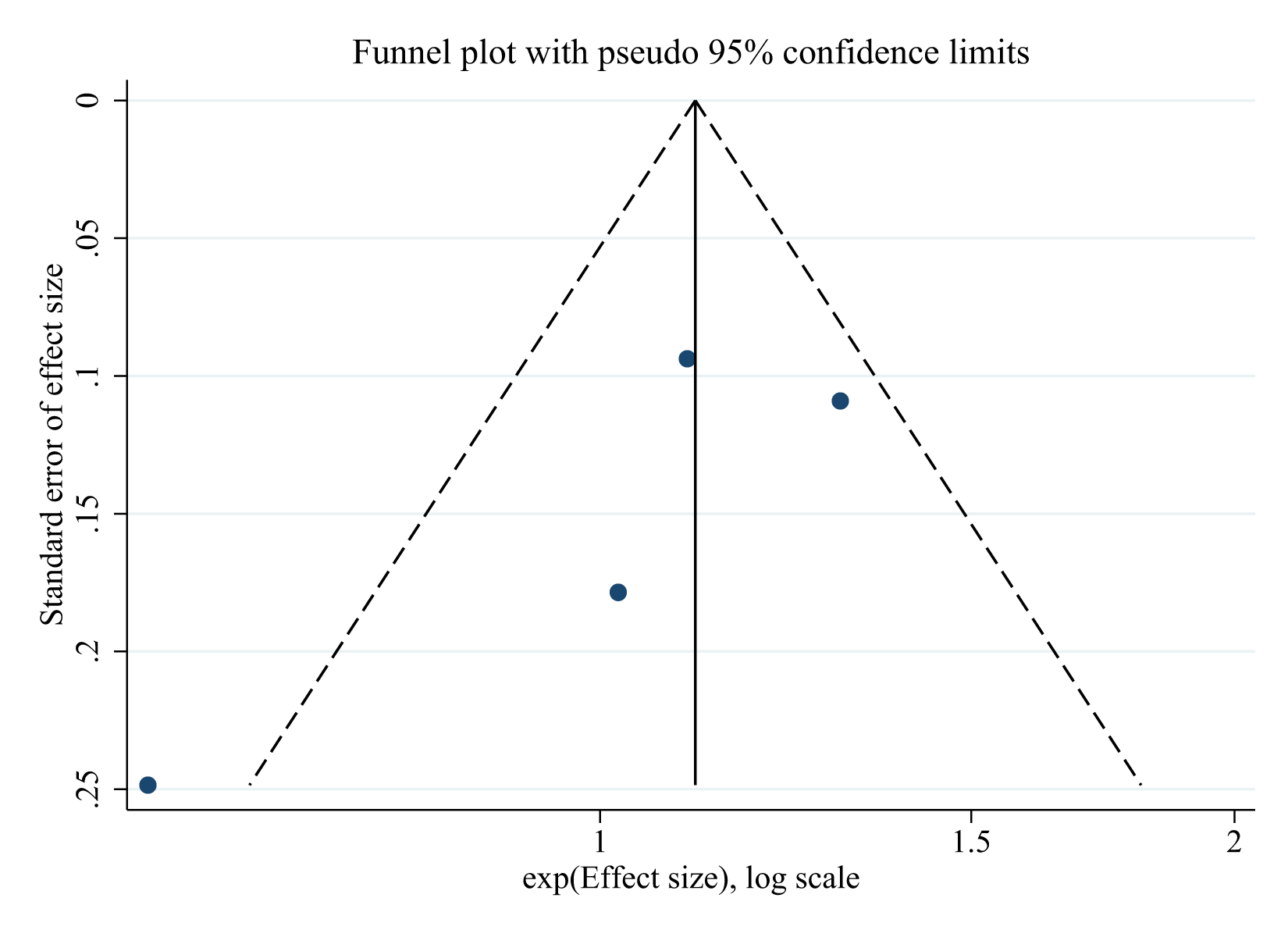

② OR


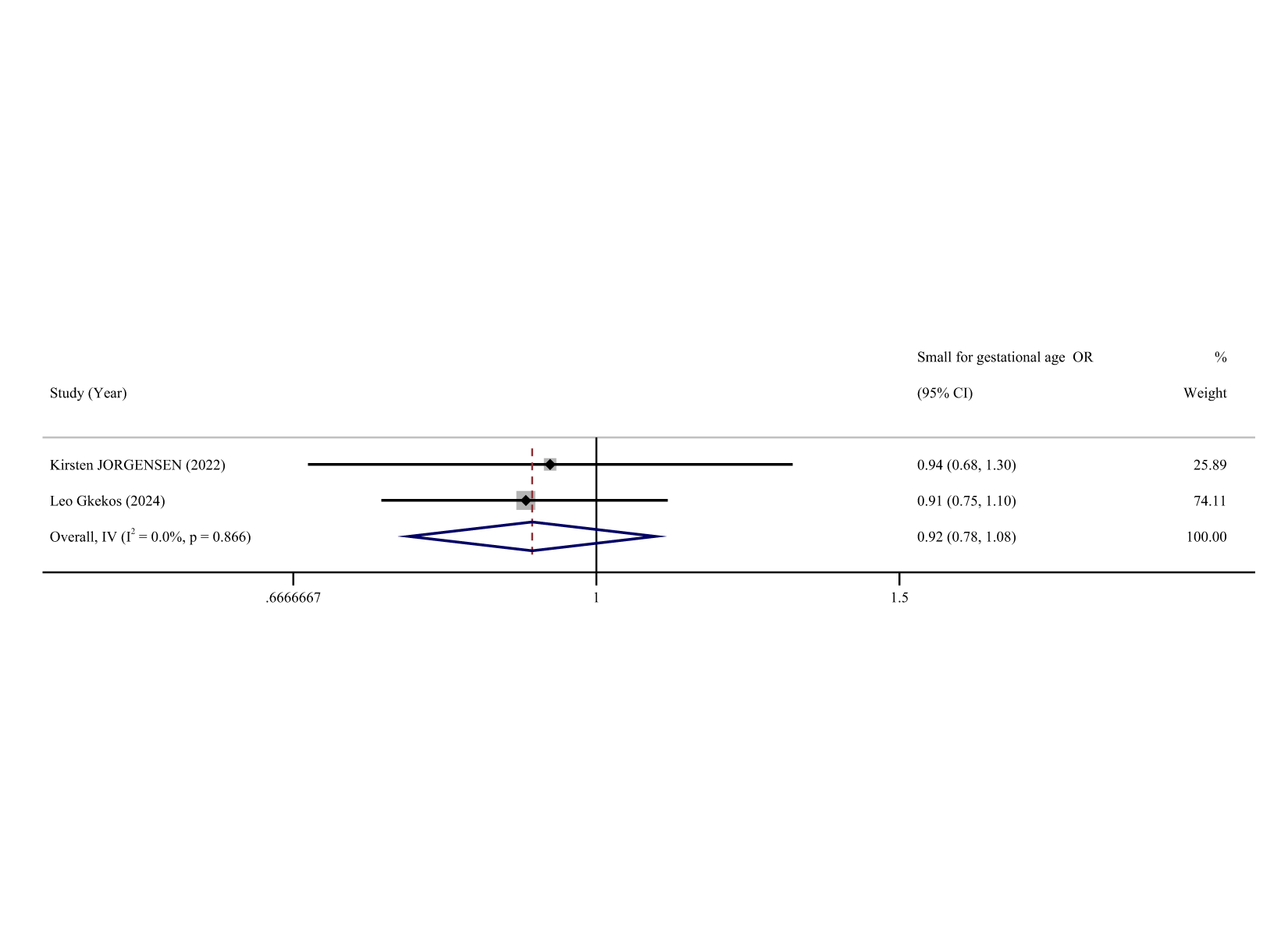


Random effect: p=0.307.

Egger’s test: not calculable


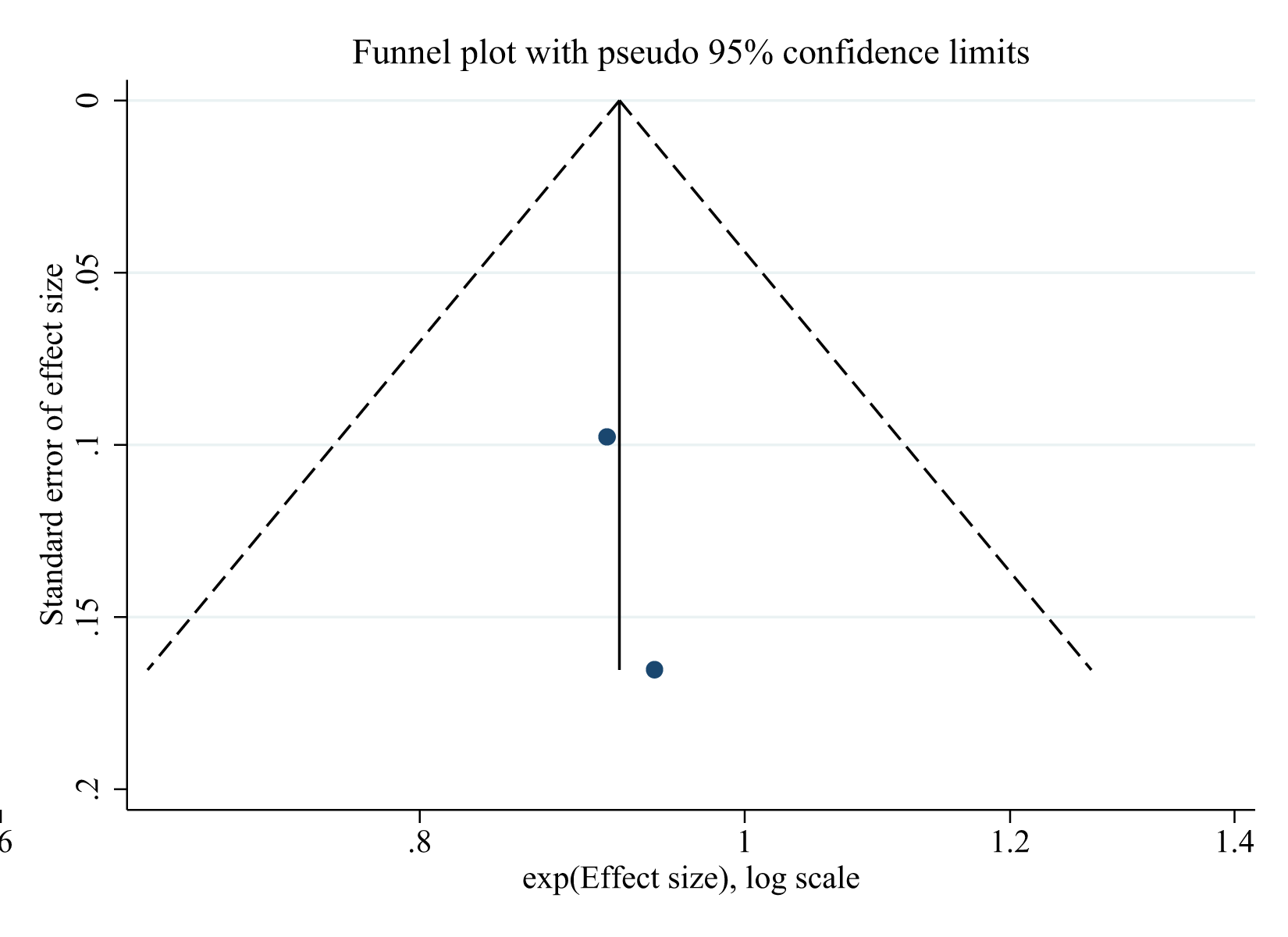

1. Stillbirth


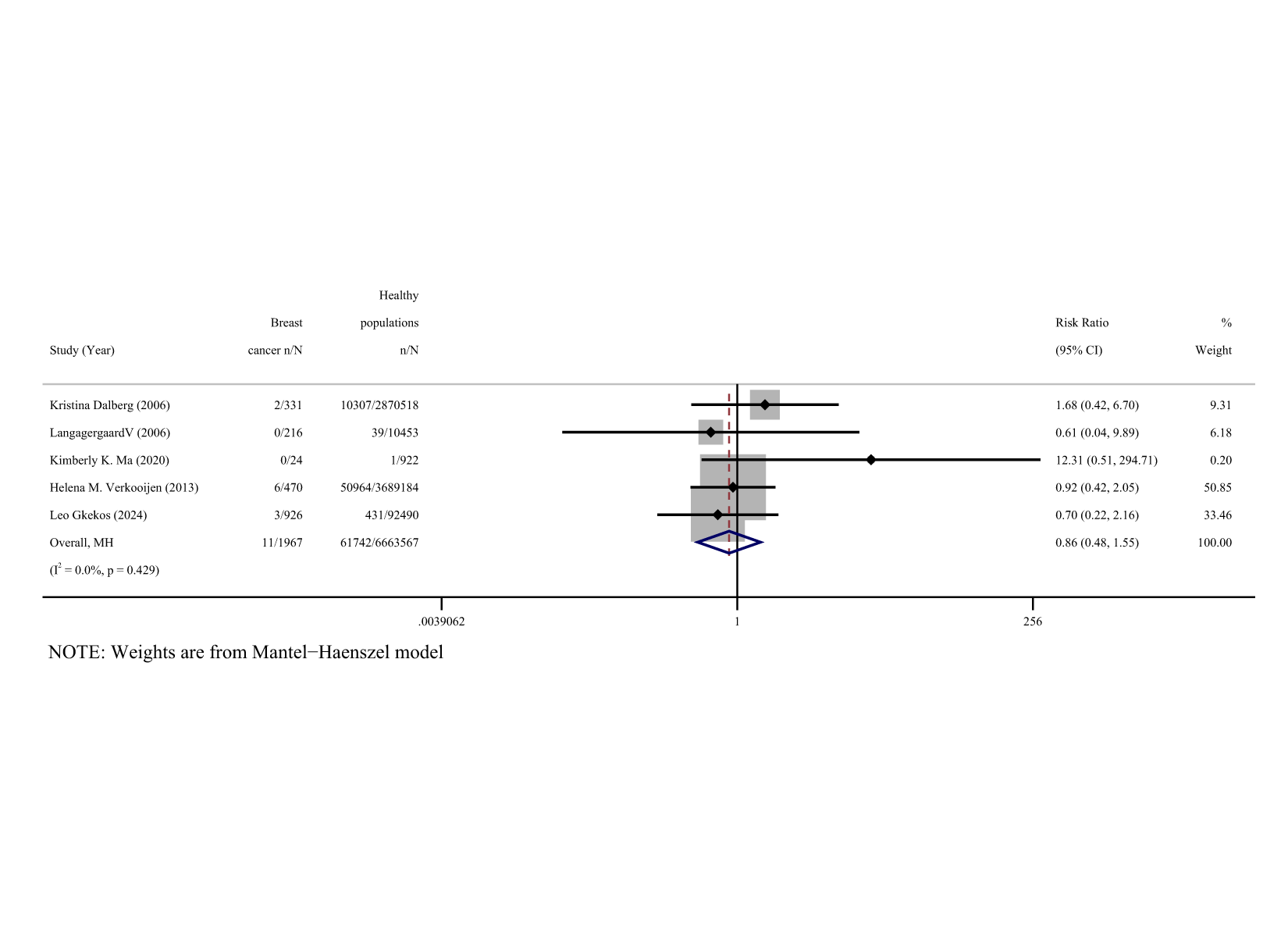


Random effect: p=0.613.

Egger’s test: p=0.344


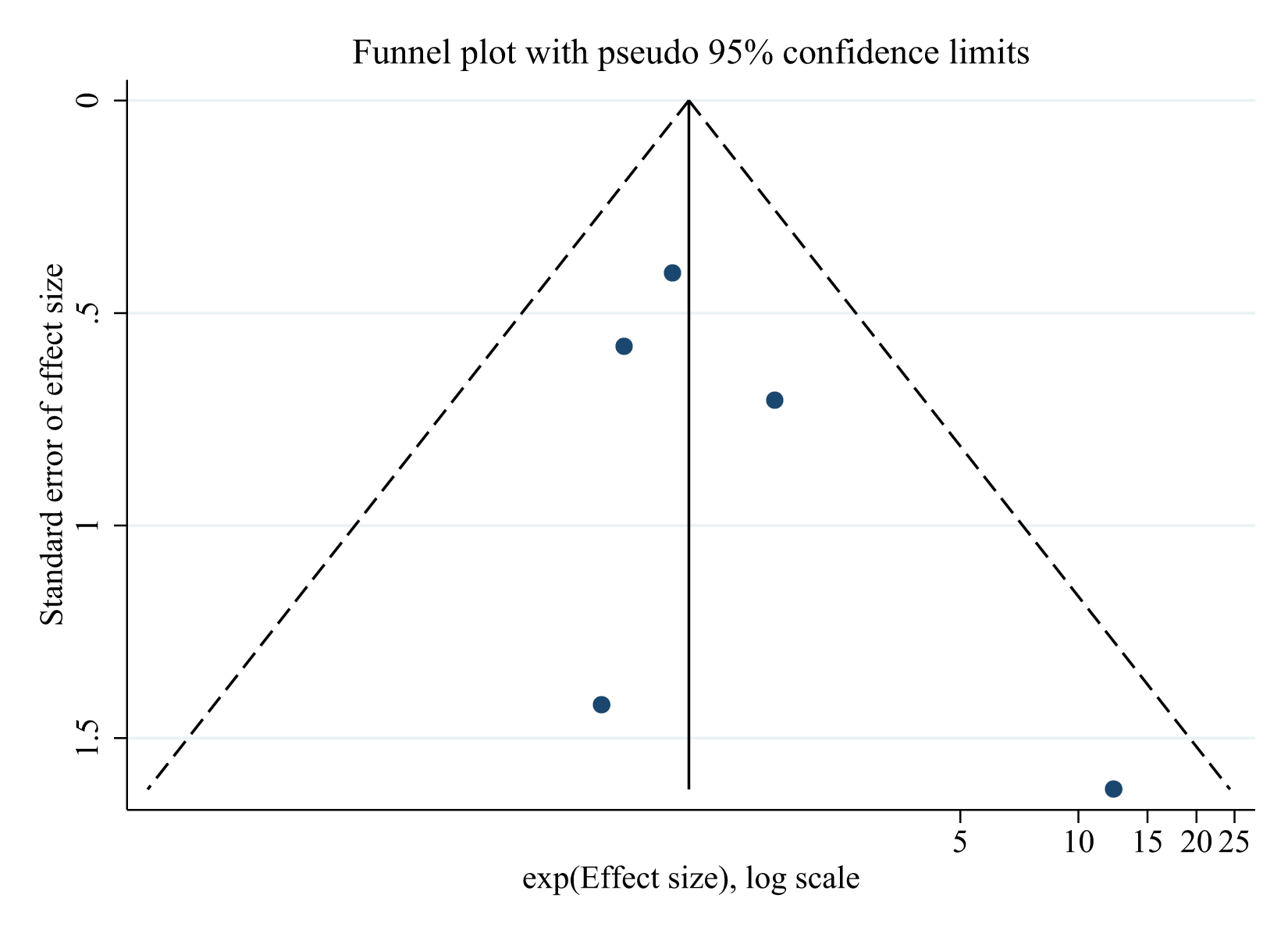

1. Live birth

① OR


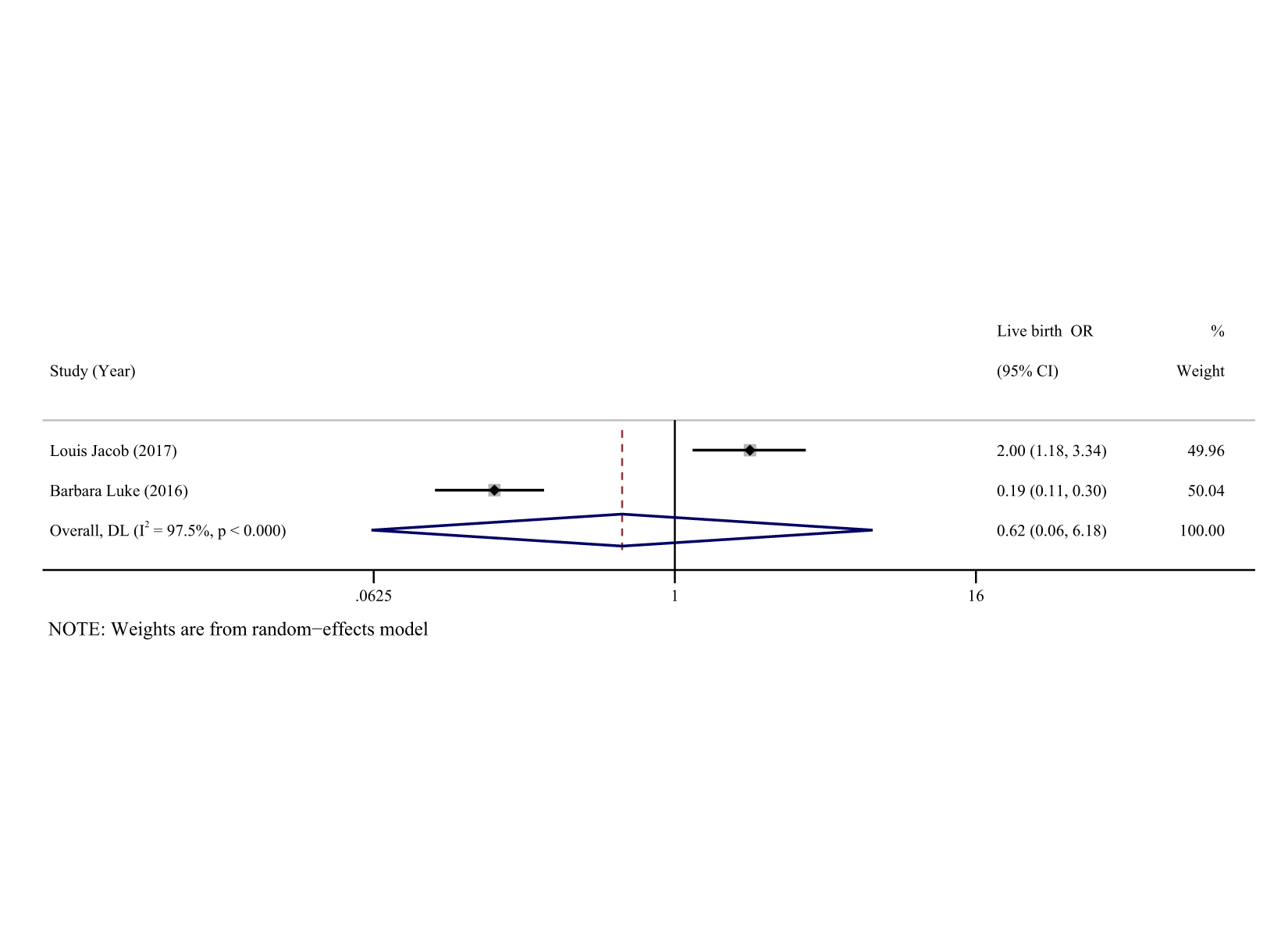


Random effect: p=0.680.

Egger’s test: not calculable


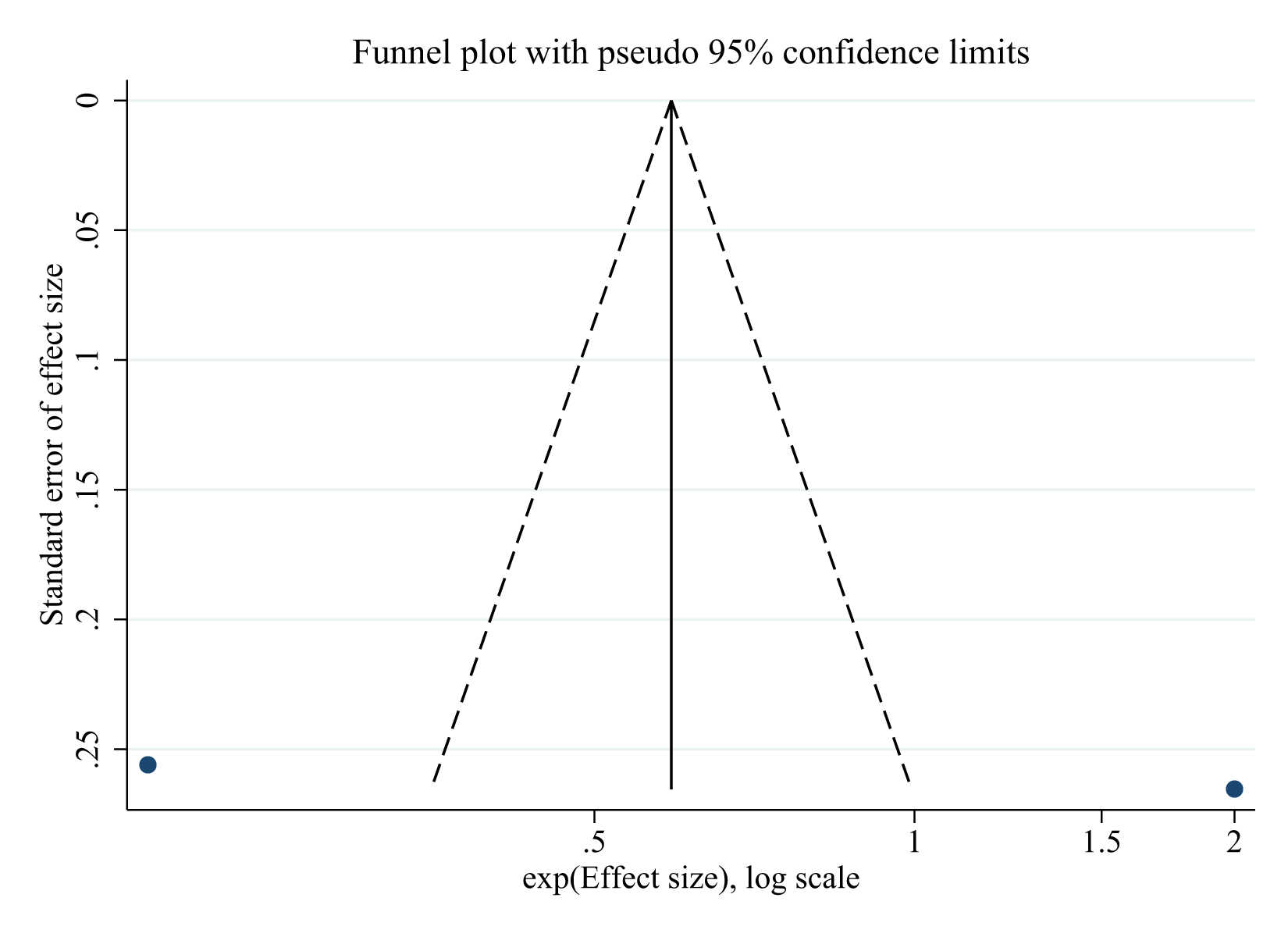

1. RR


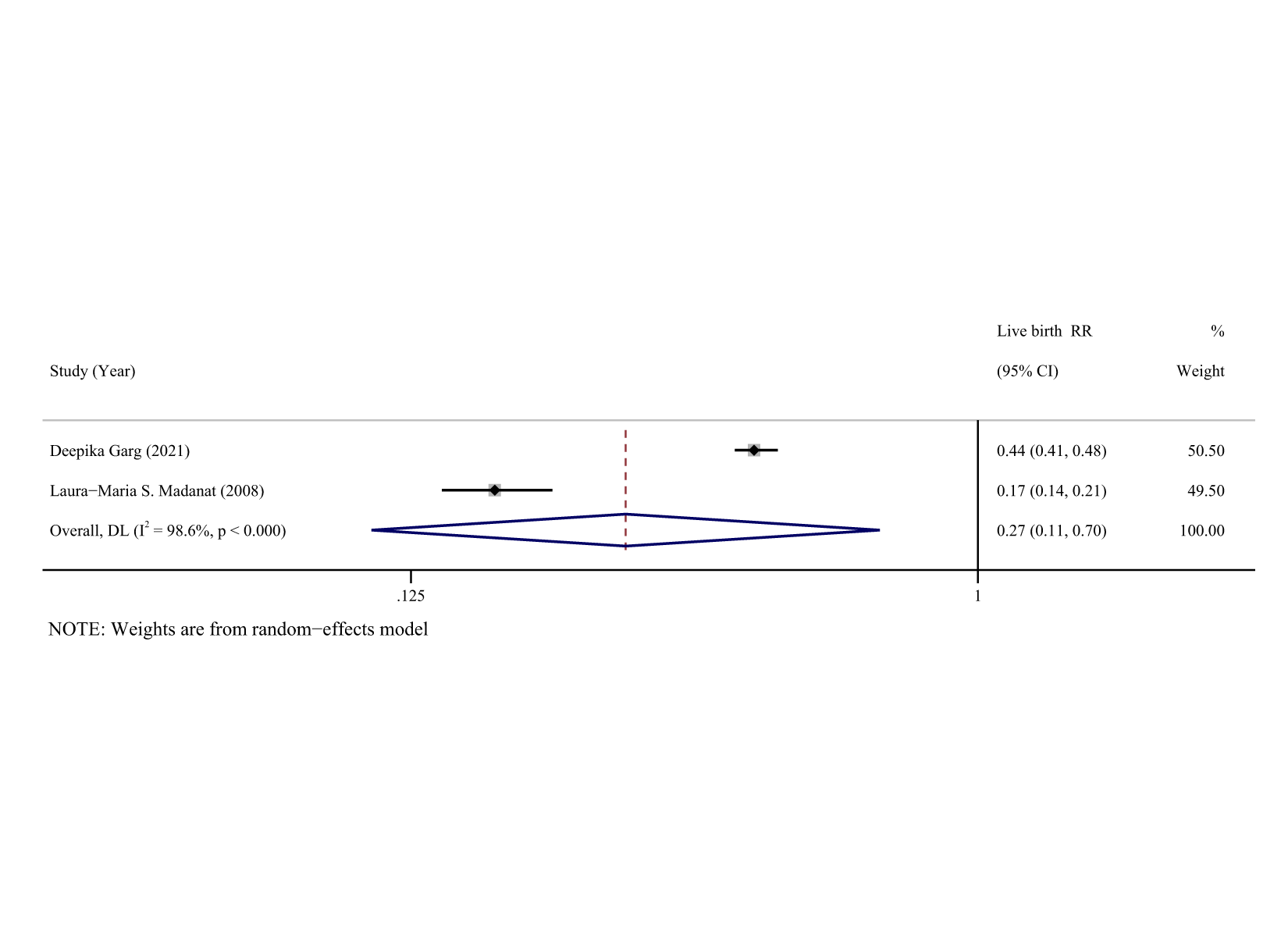


Random effect: p=0.007.

Egger’s test: not calculable


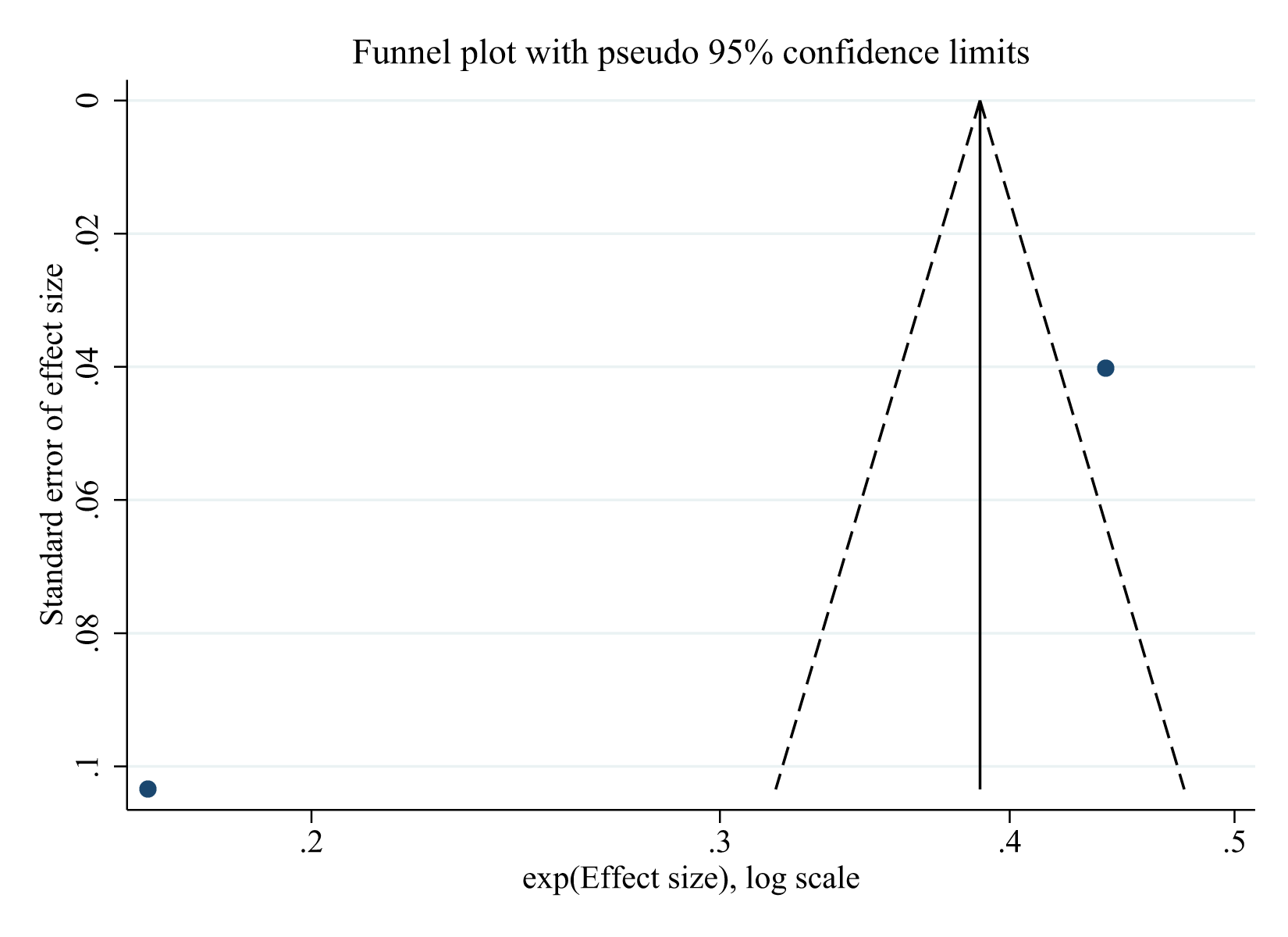

1. Binary variable


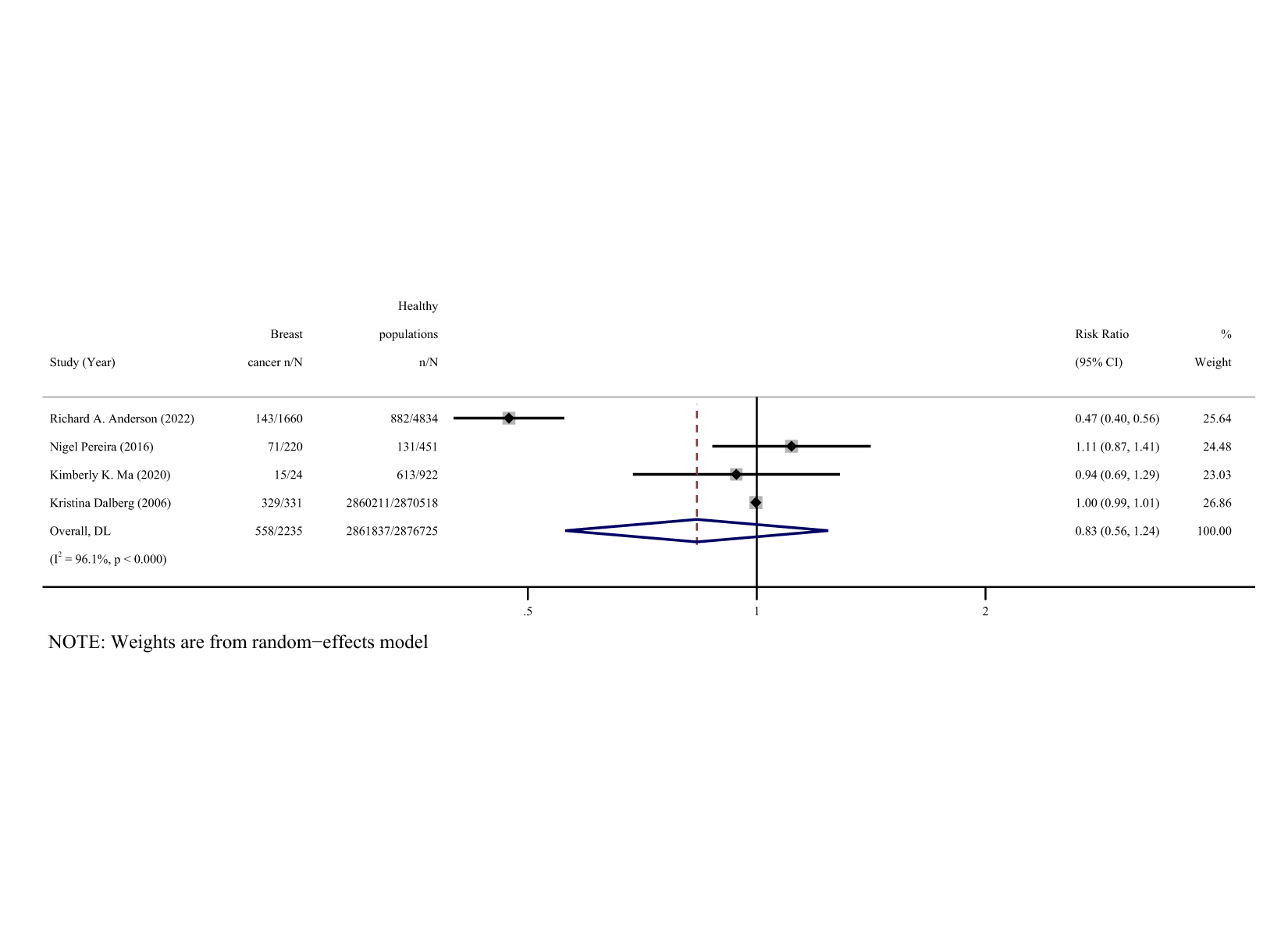


Random effect: p=0.371.

Egger’s test: p=0.468


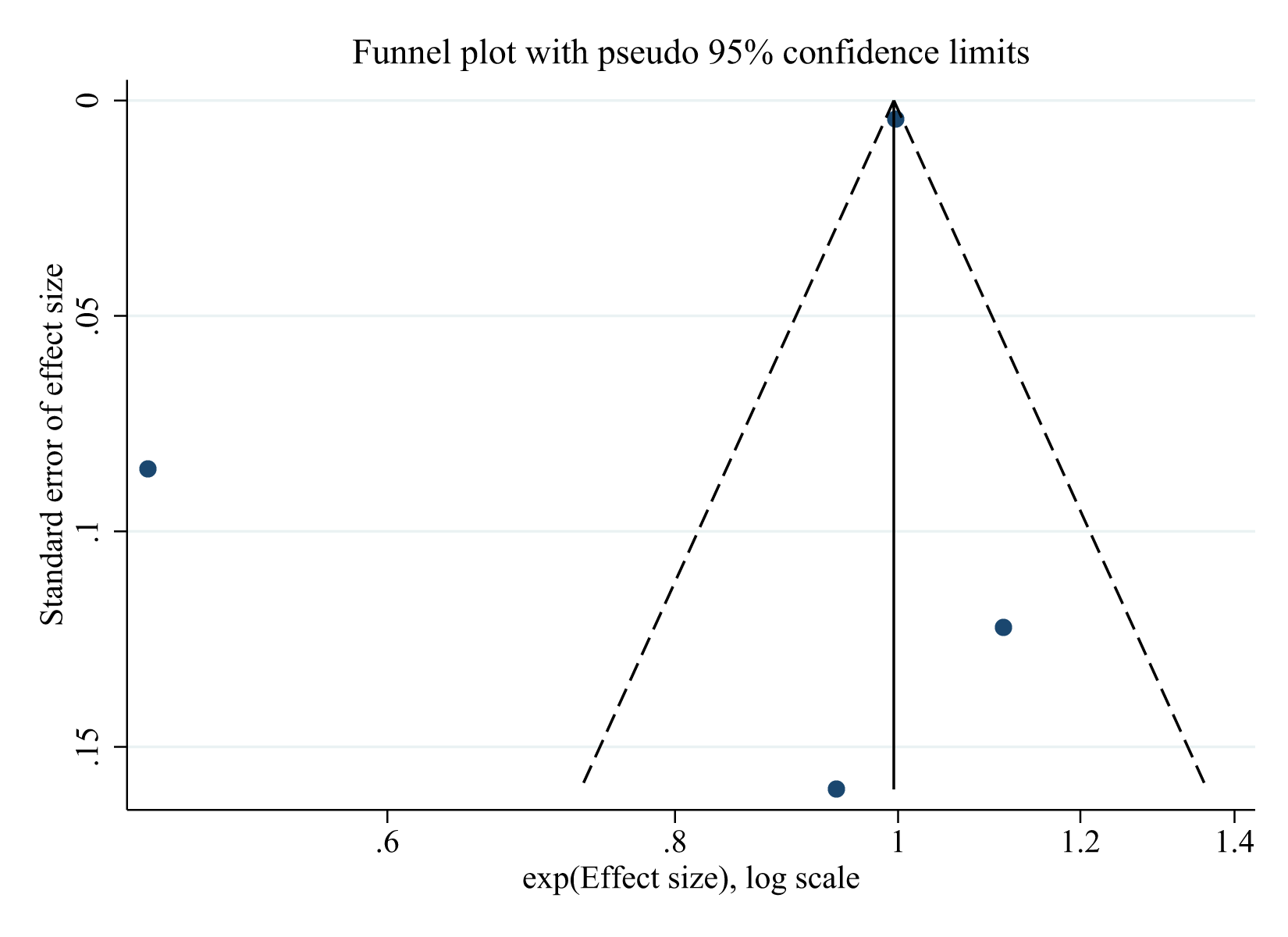

1. Apgar score (<7)


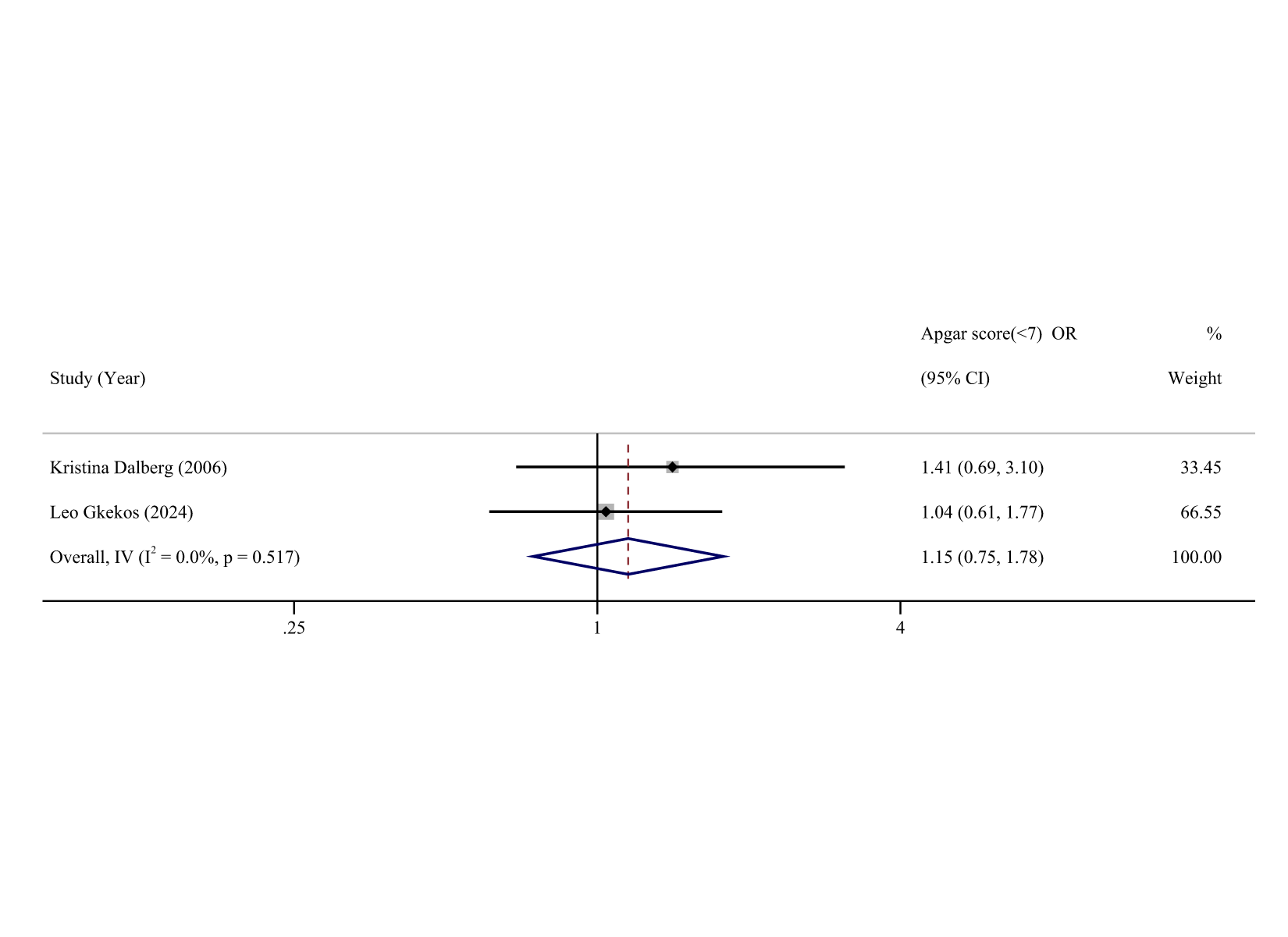


Random effect: p=0.525.

Egger’s test: not calculable


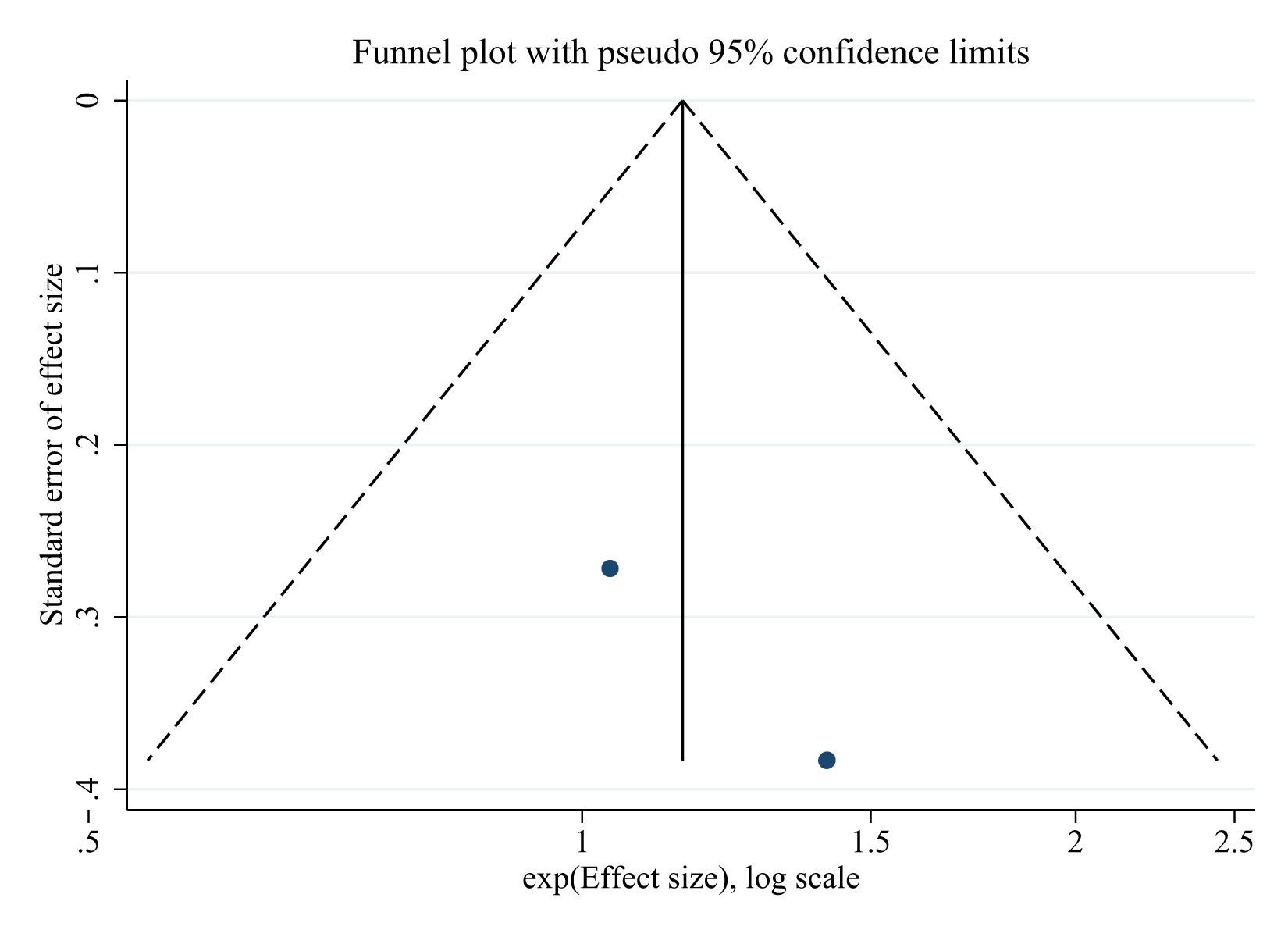

1. Fetal abnormalities

① OR


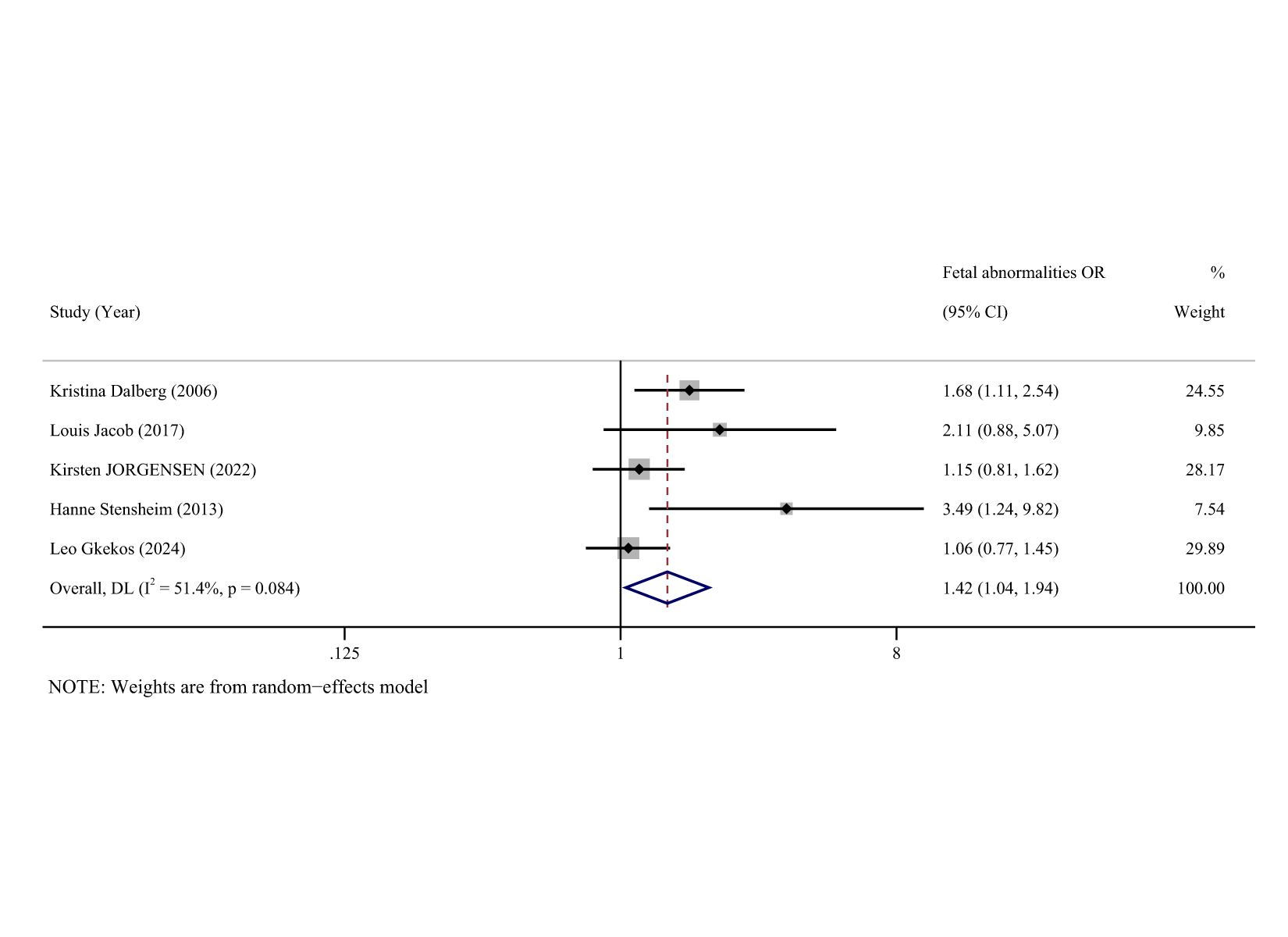


Random effect: p=0.027.

Egger’s test: p=0.047


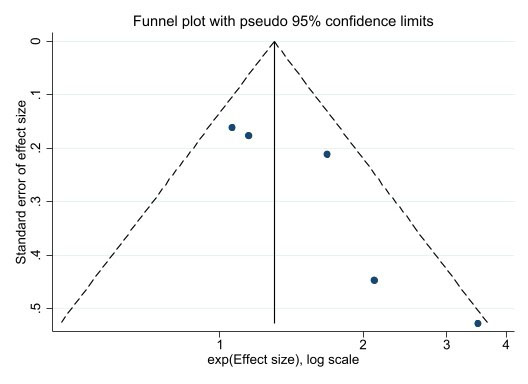

- 1. RR


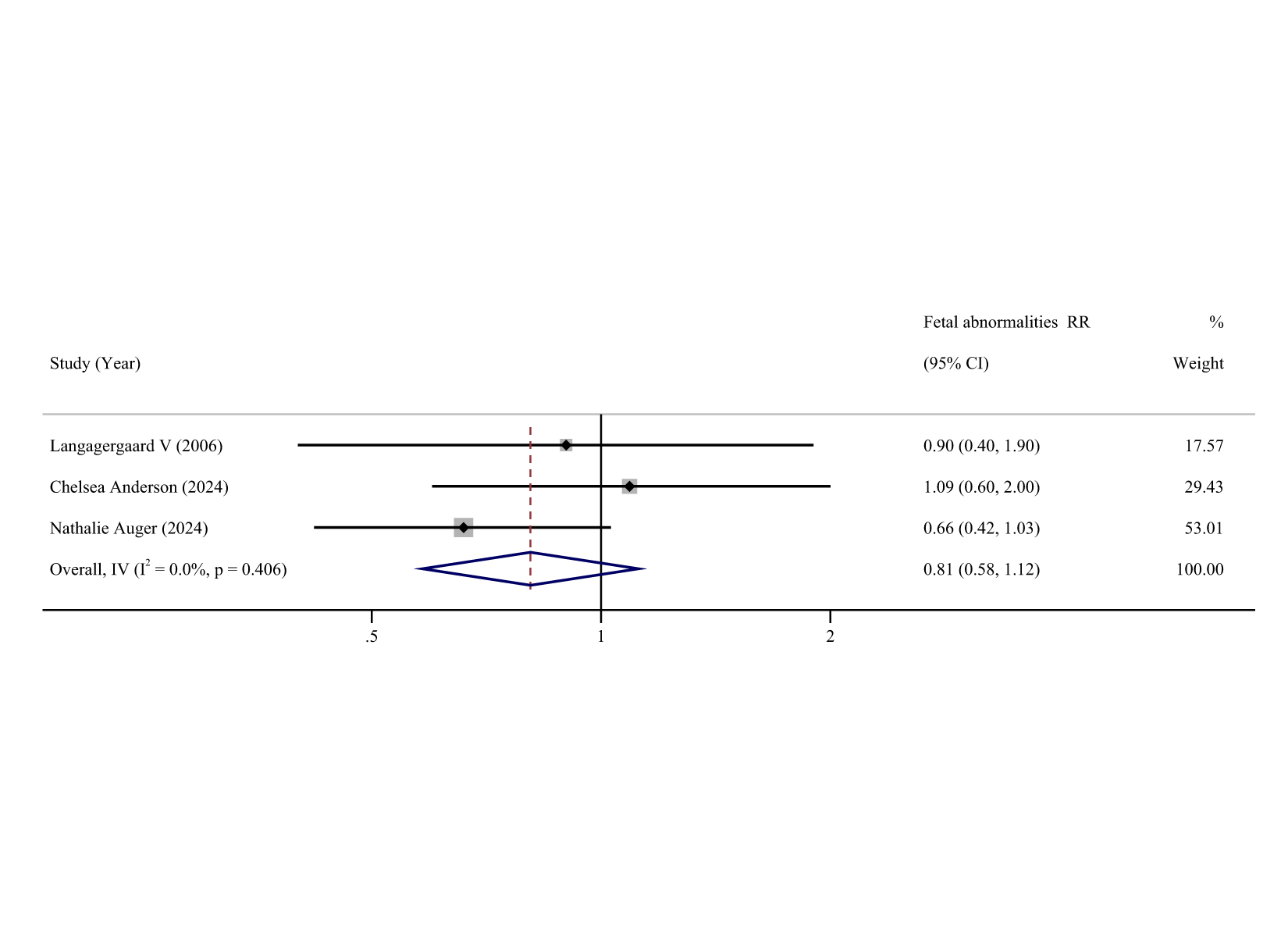


Random effect: p=0.200.

Egger’s test: p=0.496


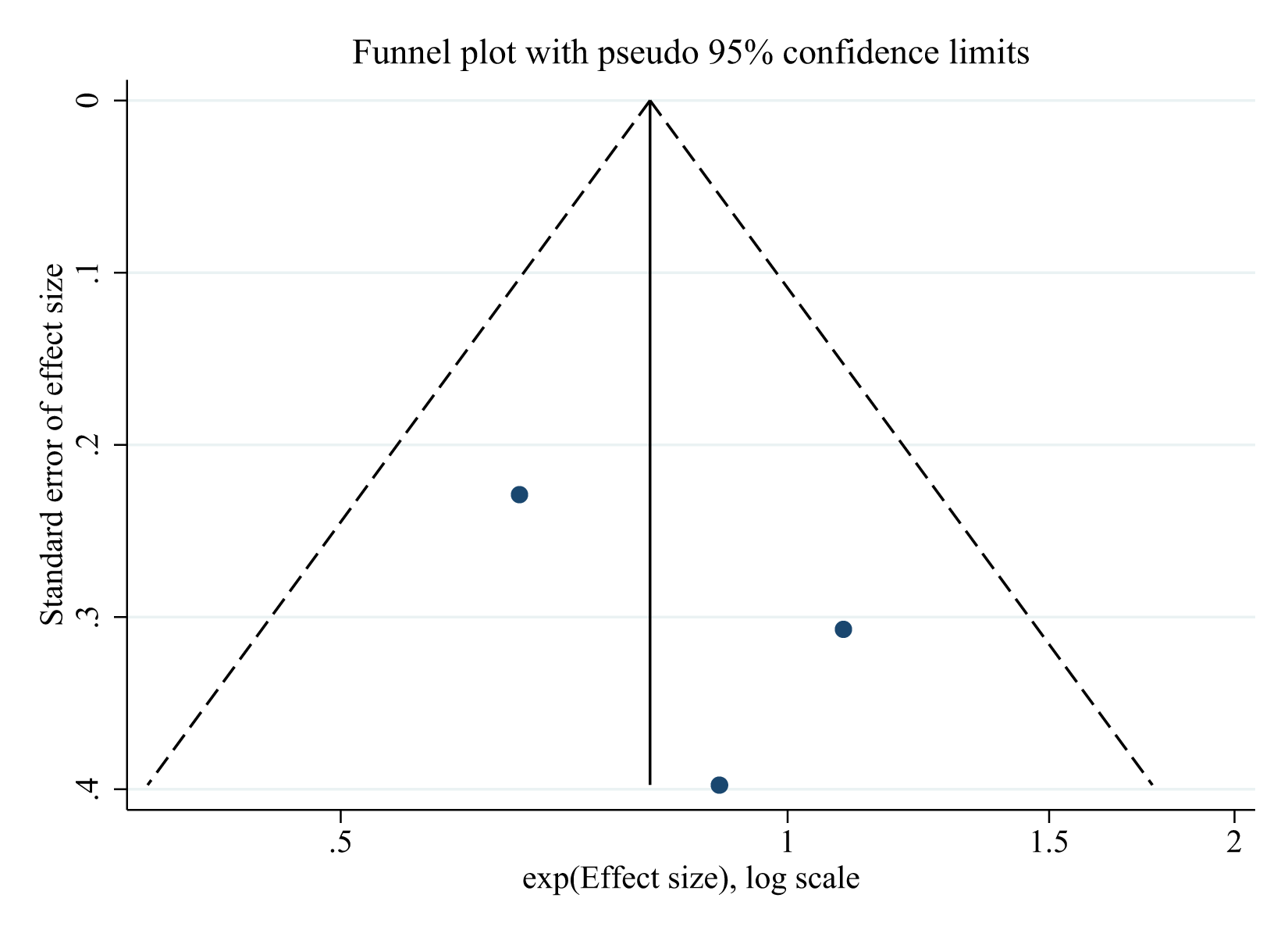

*Abbreviations: OR, odds ratio; RR, relative risk; HR, hazard ratio; CI, confidence intervals

**eFigure 4** **Pregnancy complications comparing between breast cancer patients and healthy women from the general population.**

1. Pre-eclampsia


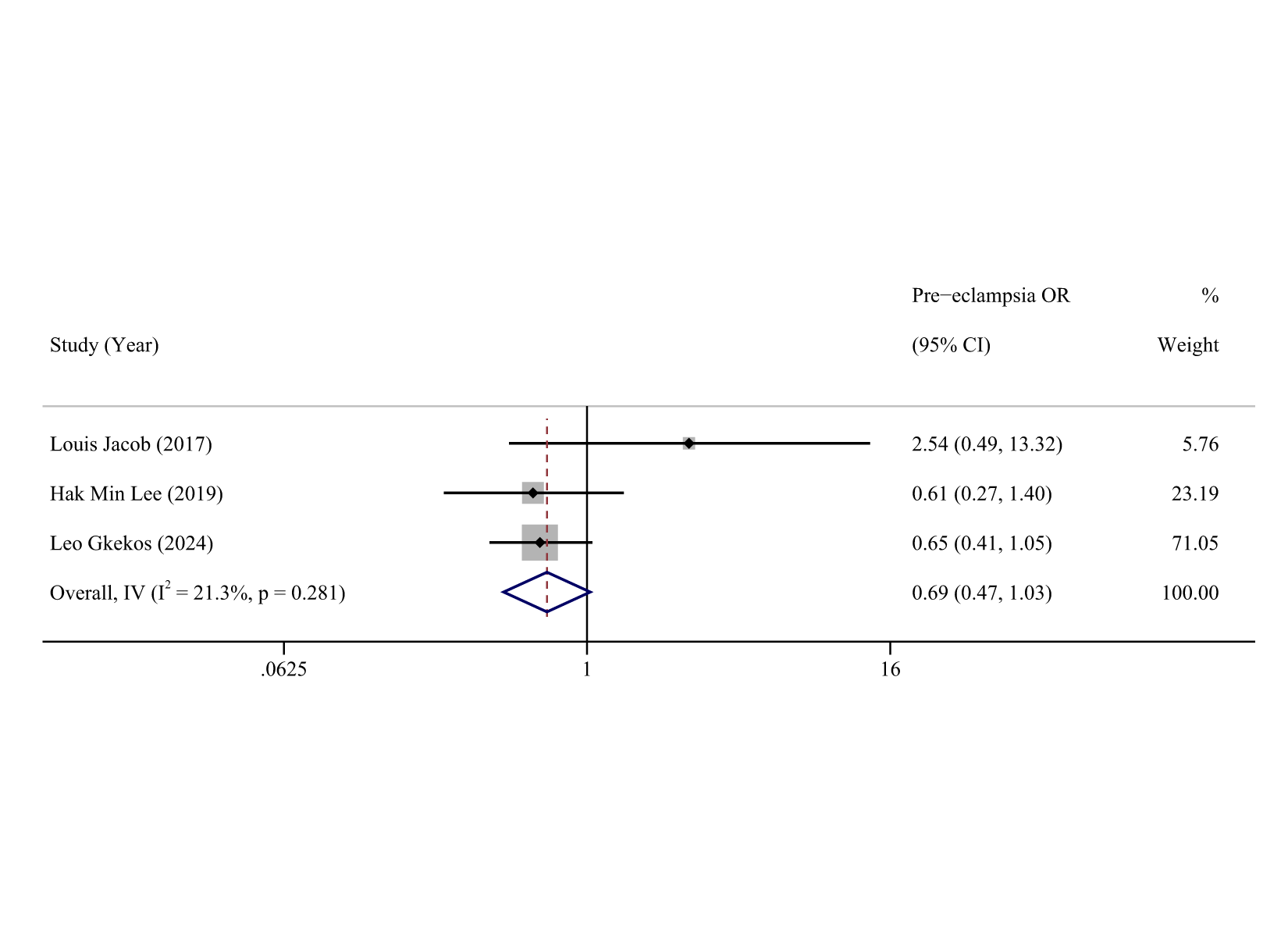


Random effect: p=0.070.

Egger’s test: p=0.386


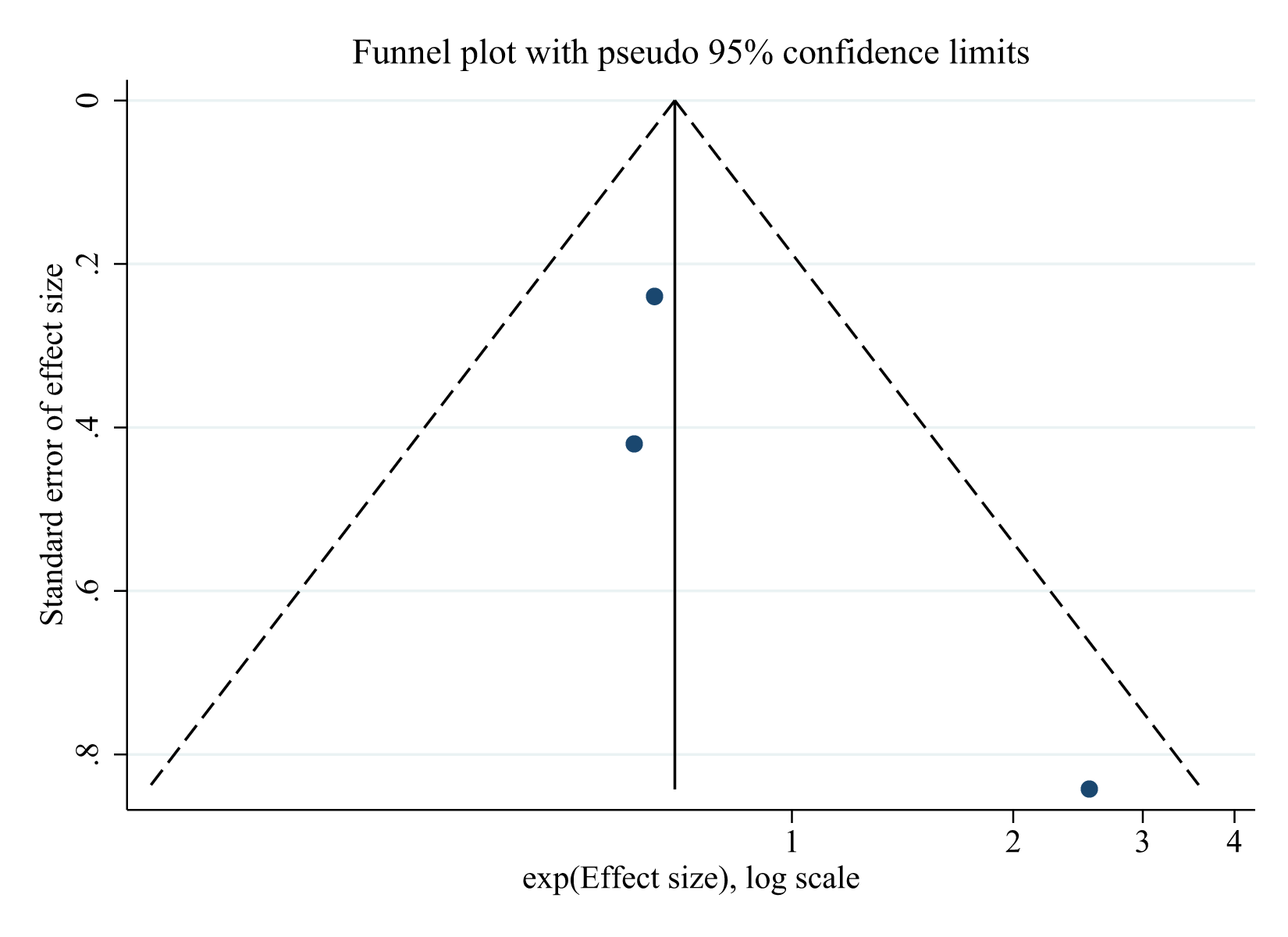

1. Pregnancy bleeding


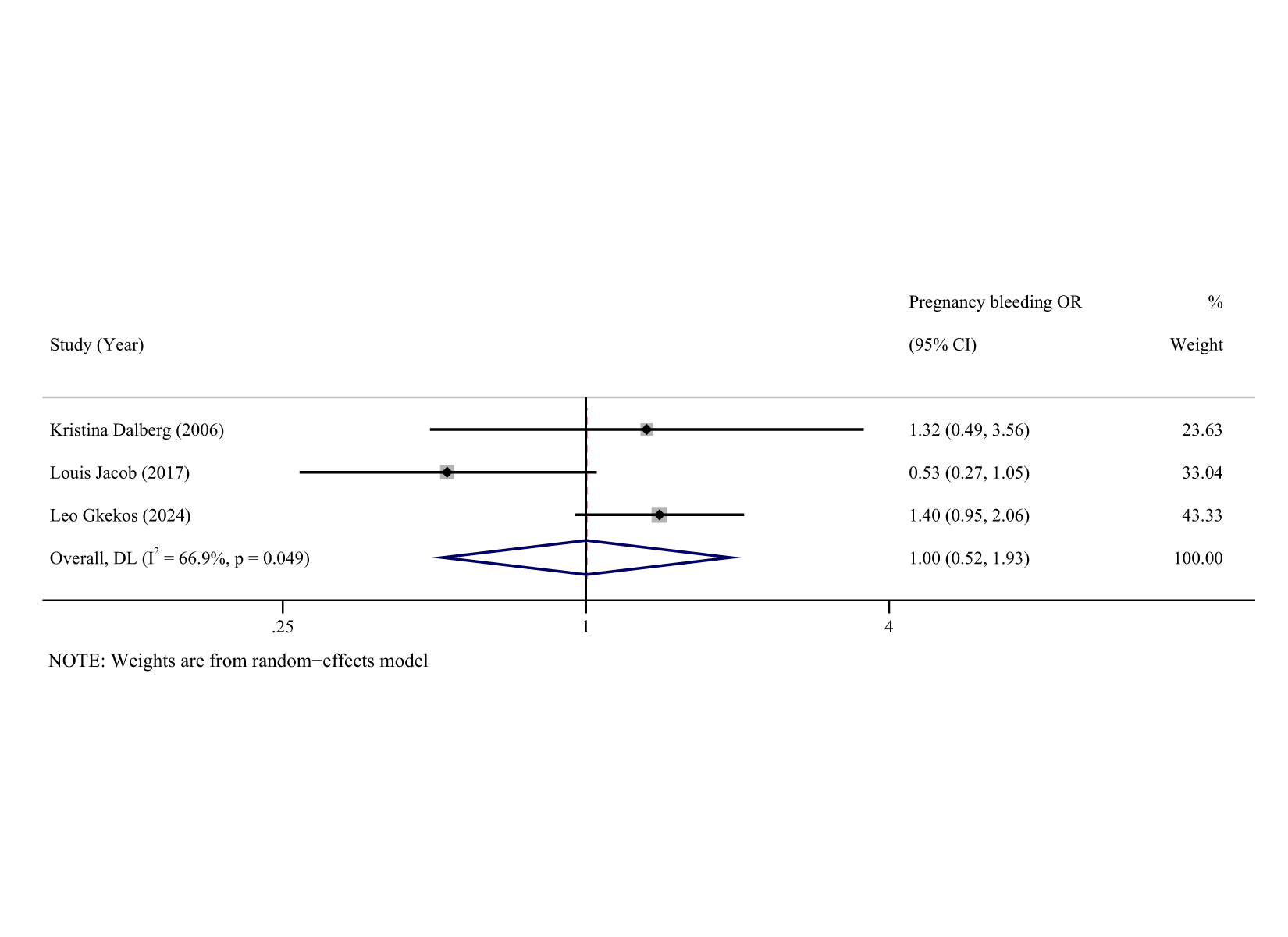


Random effect: p=0.996.

Egger’s test: p=0.684


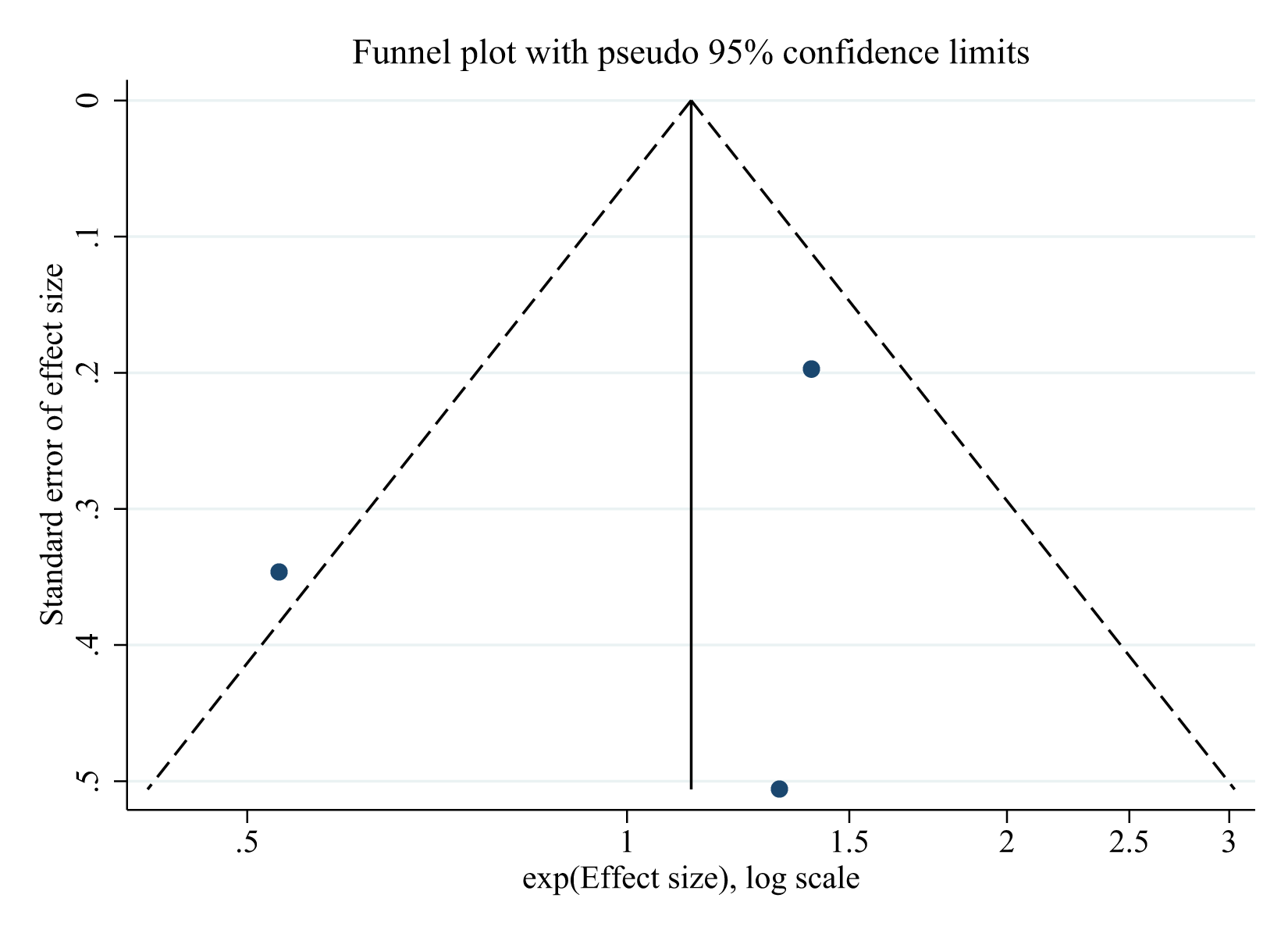

1. Gestational diabetes


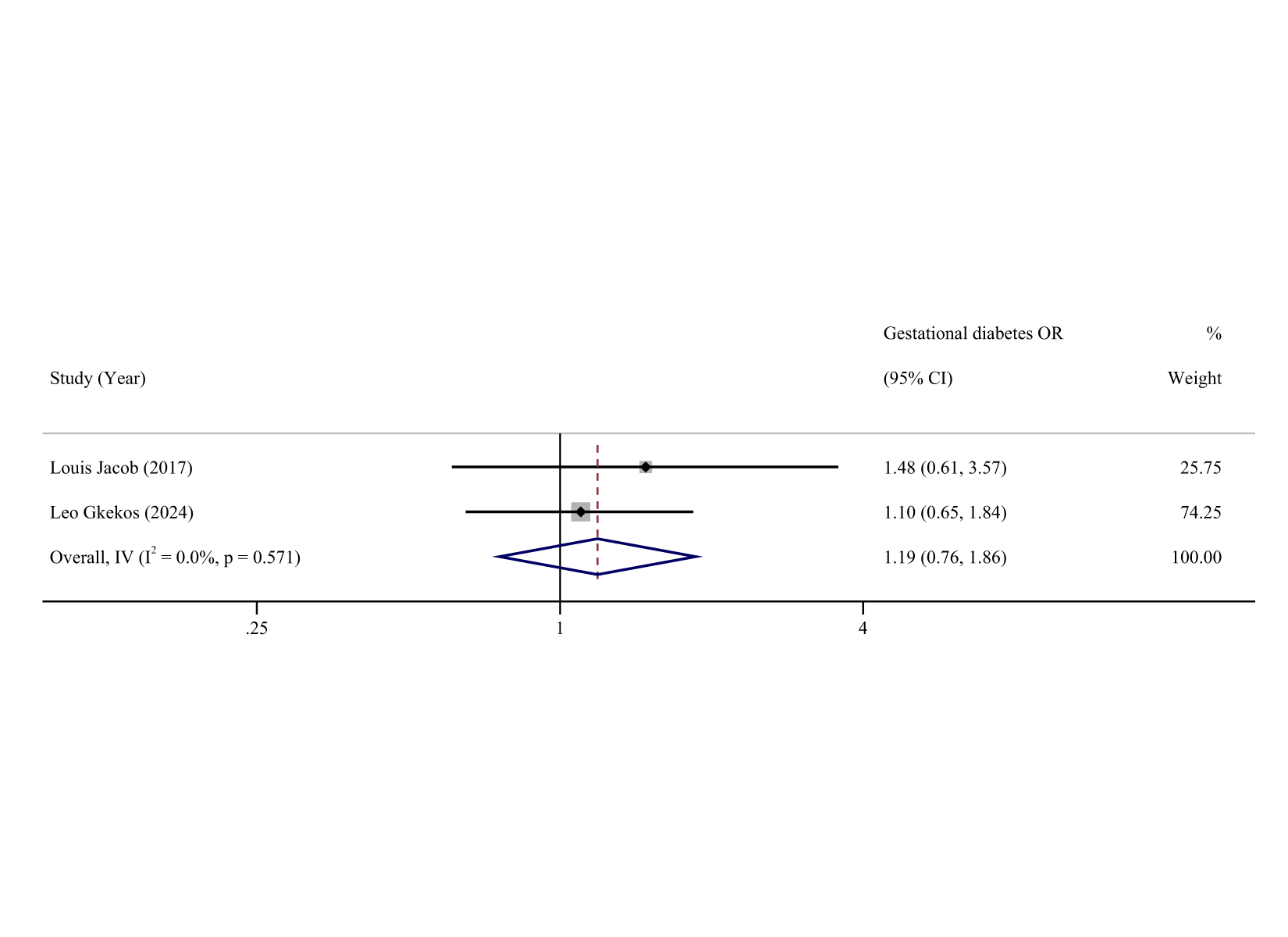


Random effect: p=0.453.

Egger’s test: not calculable


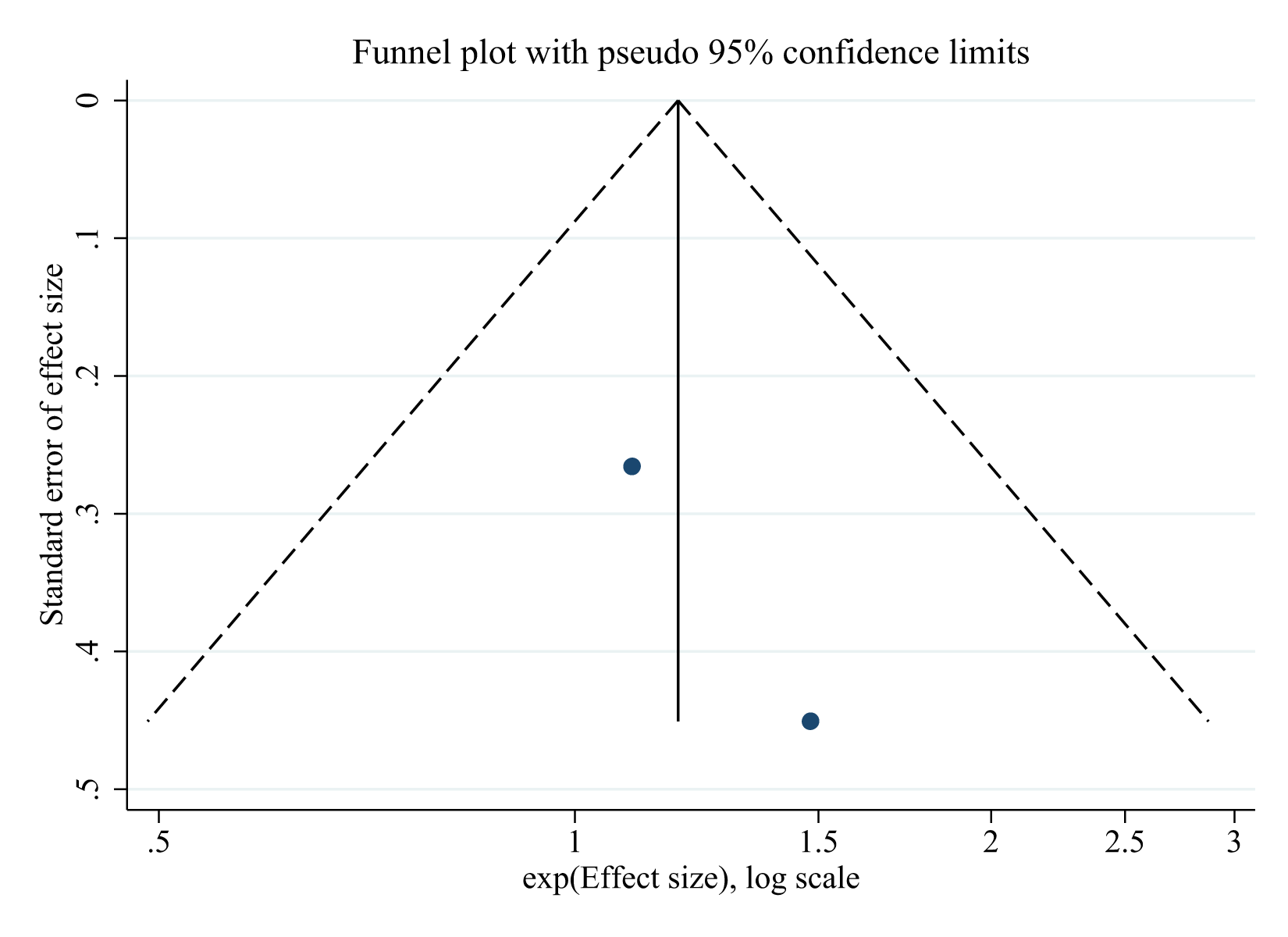

Supplement: Supplementary file 3 [file SupplementaryFile3.docx]
