# Supplementary figures and images for: Reproductive outcomes of pregnancy after breast cancer: an updated systematic review and meta-analysis
Source: Front Oncol. 2025 Sep 26;15:1569109. doi: 10.3389/fonc.2025.1569109 (PMC12510861; doi:10.3389/fonc.2025.1569109)

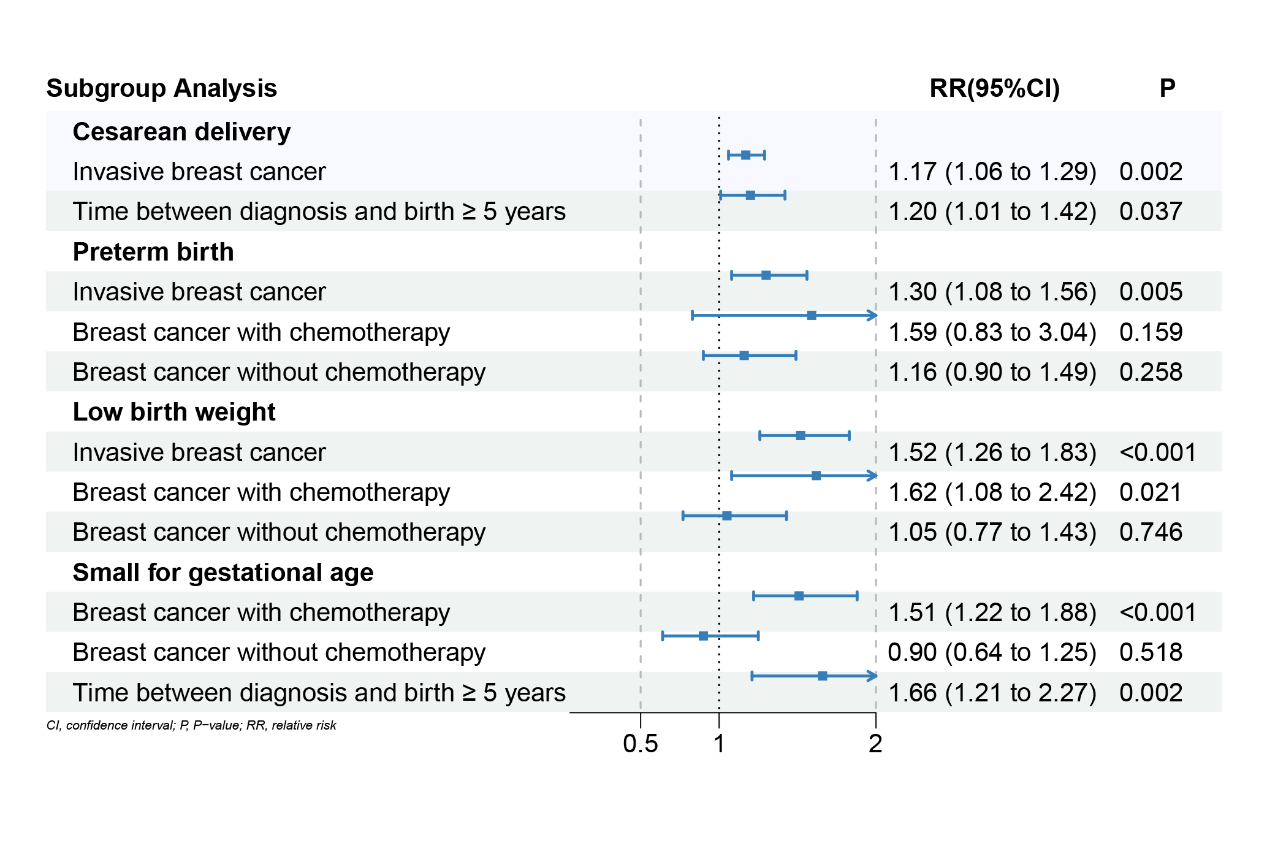


eFigure 5 Subgroup analysis

Supplement: Supplementary file 4 [file SupplementaryFile4.docx]
